# Supplementary material for: Whose sample is it anyway? Widespread misannotation of samples in transcriptomics studies
Source: F1000Res. 2016 Sep 30;5:2103. Originally published 2016 Aug 30. [Version 2] doi: 10.12688/f1000research.9471.2 (PMC5034794; doi:10.12688/f1000research.9471.2)
Supplement: Supplementary file 3 [file f1000research-5-10437-s0000.tgz › 26fed2e8-ed2e-4810-8124-a1d85920dbef.pdf]

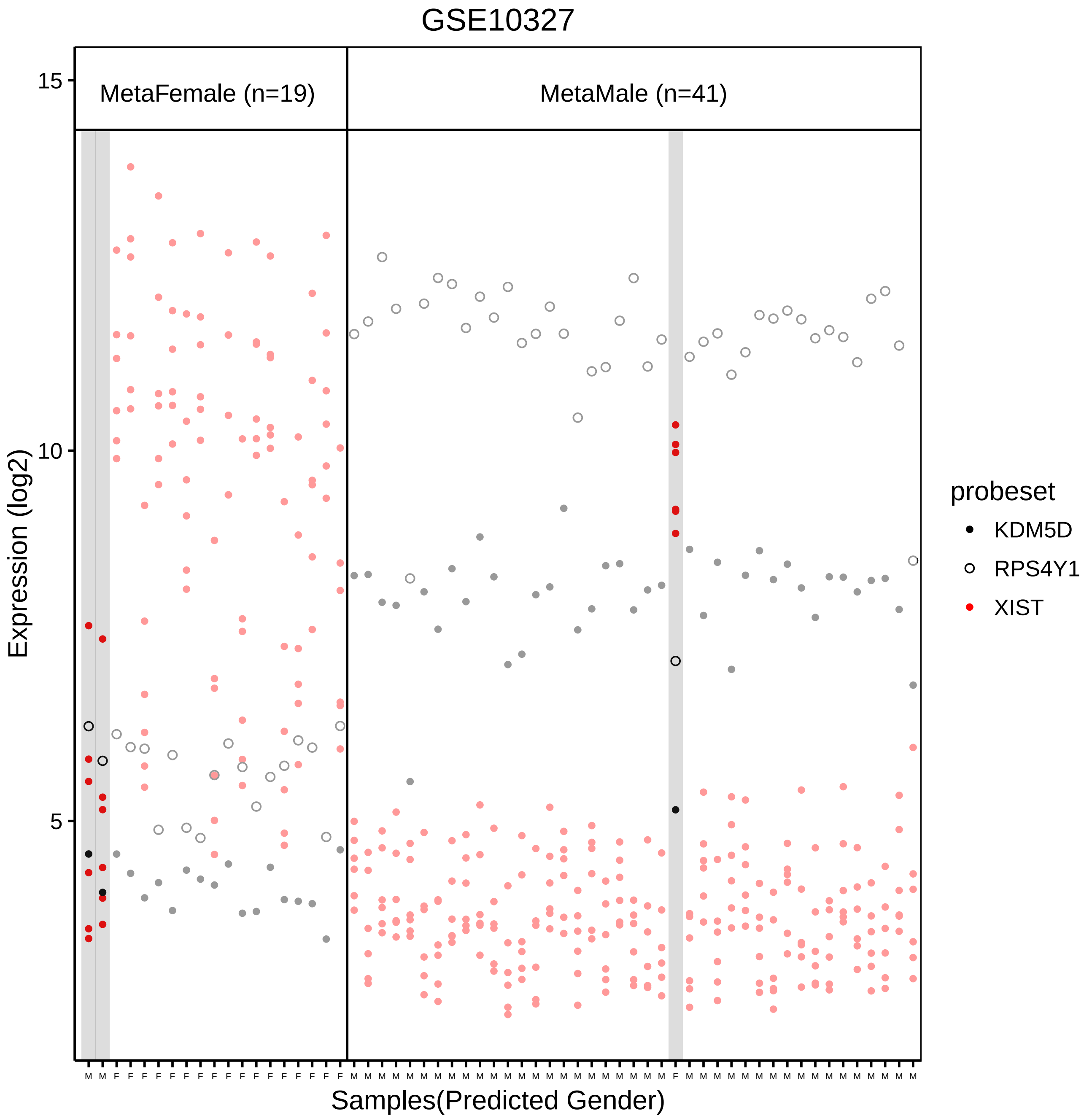

GSE10327
MetaFemale (n=19)
MetaMale (n=41)
Expression (log2)
15
10
5
Samples(Predicted Gender)
probeset
KDM5D
RPS4Y1
XIST

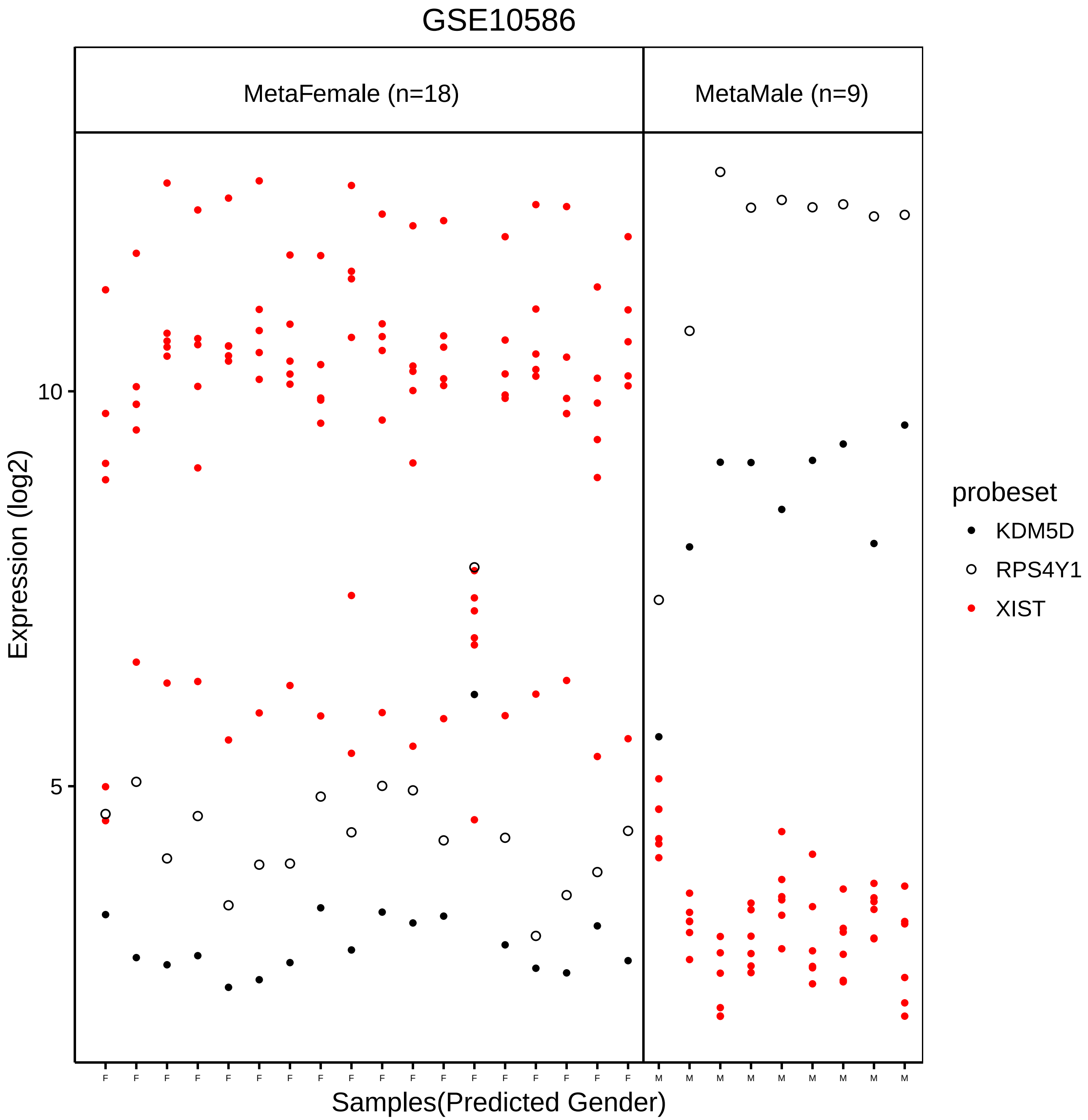
GSE10586
MetaFemale (n=18)
MetaMale (n=9)
Expression (log2)
10
5
F
M
Samples(Predicted Gender)
probeset
KDM5D
RPS4Y1
XIST

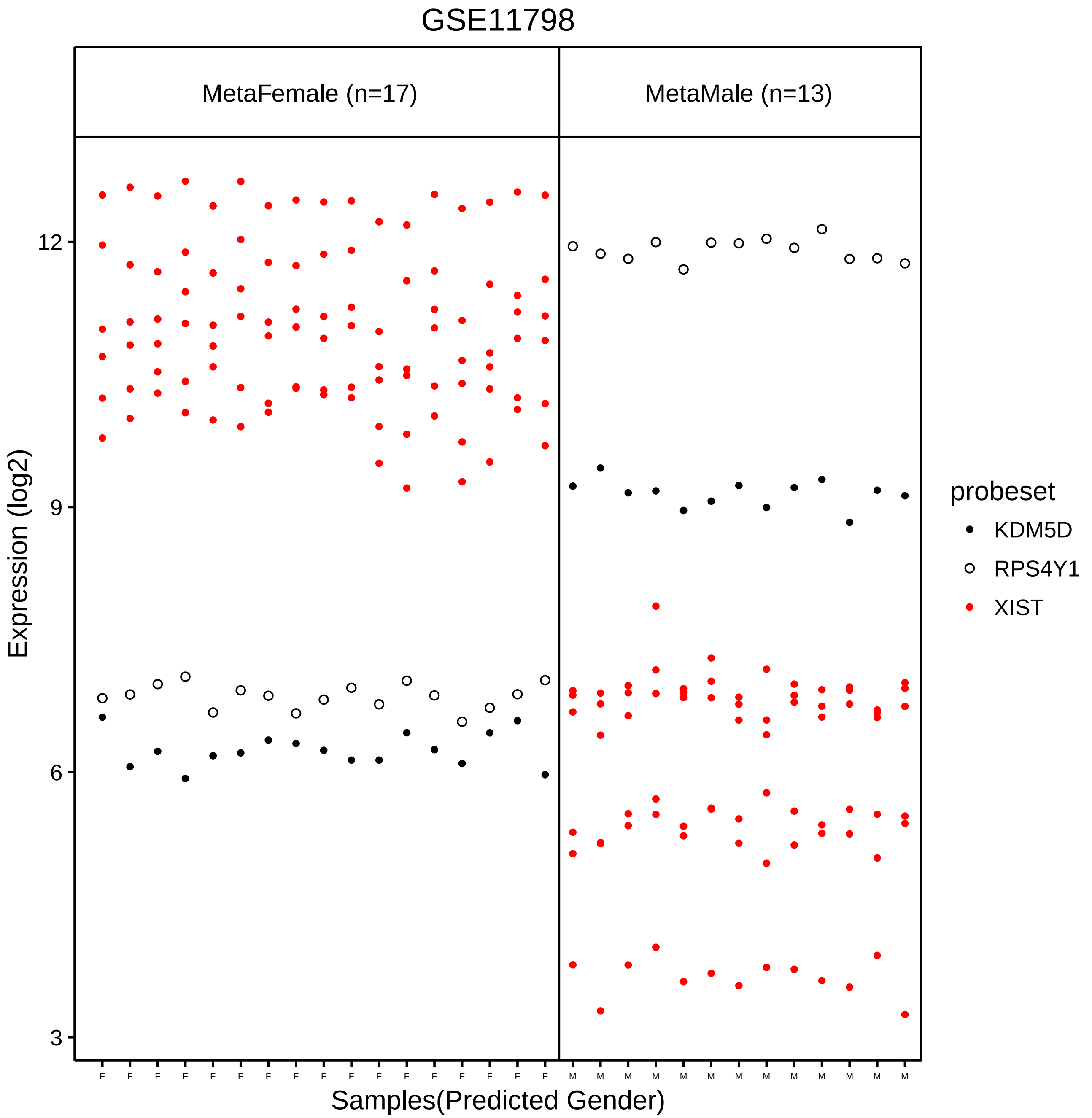

GSE11798
MetaFemale (n=17)
MetaMale (n=13)
Expression (log2)
12
9
6
3
F
M
Samples(Predicted Gender)
probeset
KDM5D
RPS4Y1
XIST

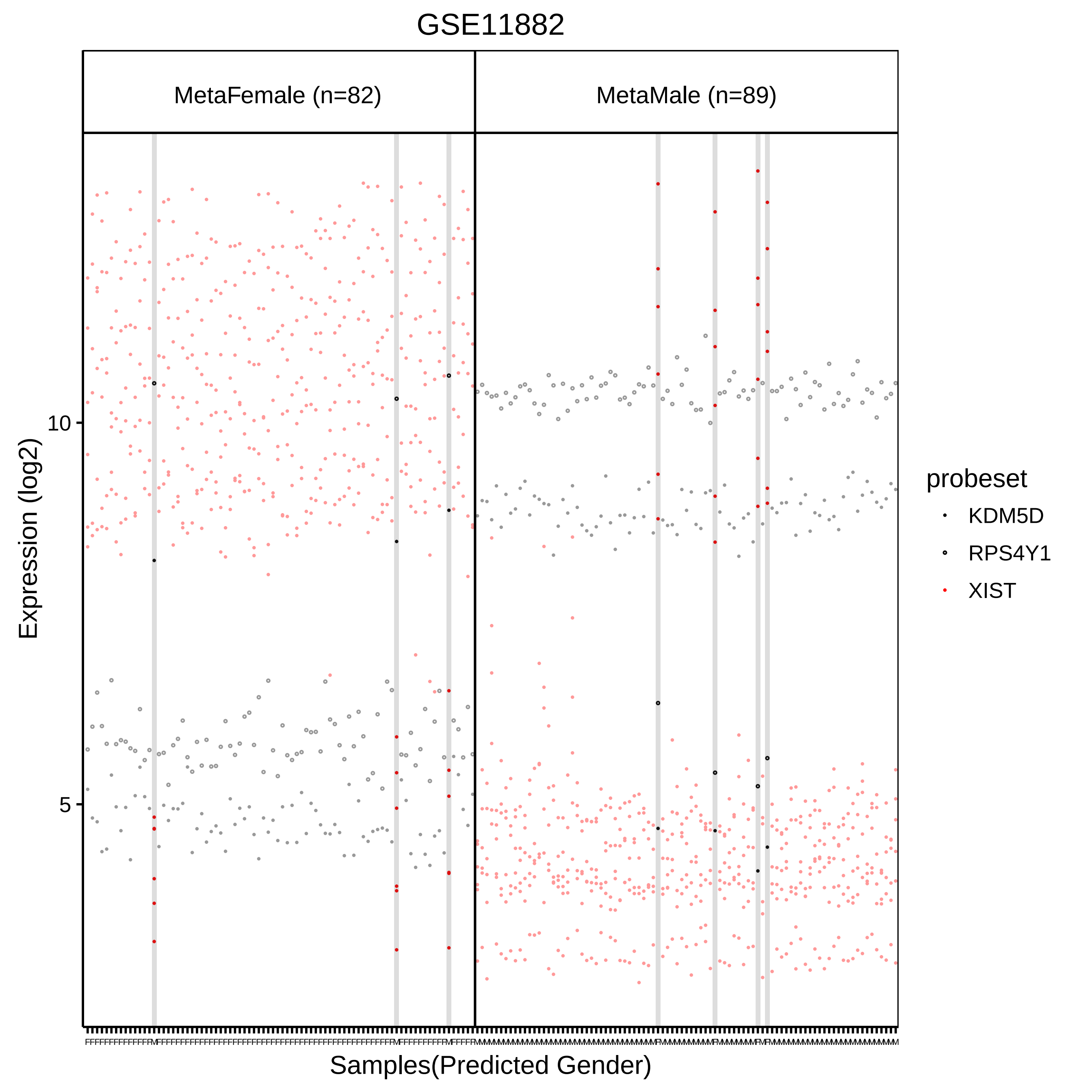

GSE11882
MetaFemale (n=82)
MetaMale (n=89)
Expression (log2)
10
5
Samples(Predicted Gender)
probeset
KDM5D
RPS4Y1
XIST

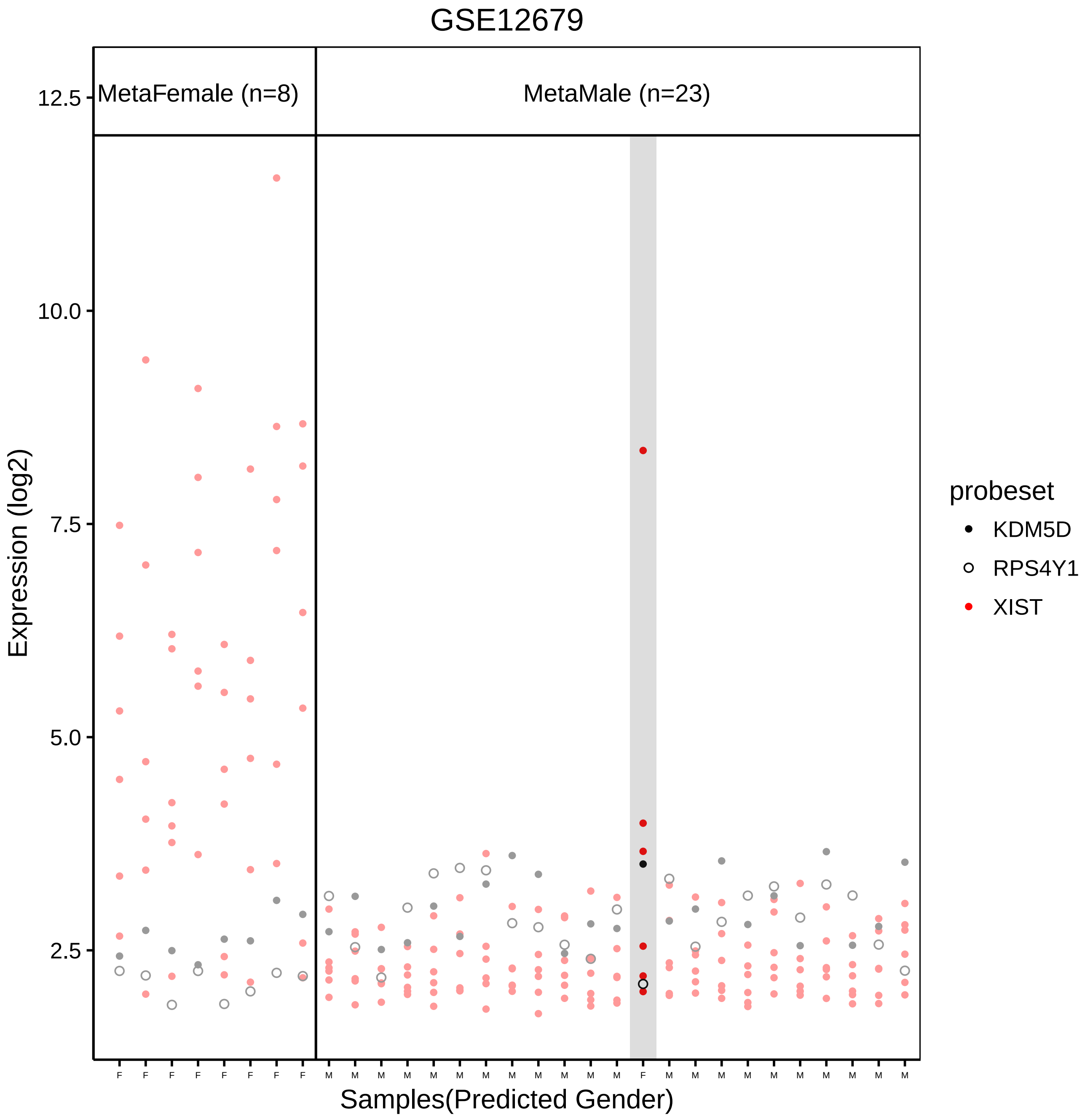

GSE12679
MetaFemale (n=8)
MetaMale (n=23)
Expression (log2)
12.5
10.0
7.5
5.0
2.5
F F F F F F F F M M M M M M M M M M M M F M M M M M M M M M M
Samples(Predicted Gender)
probeset
KDM5D
RPS4Y1
XIST

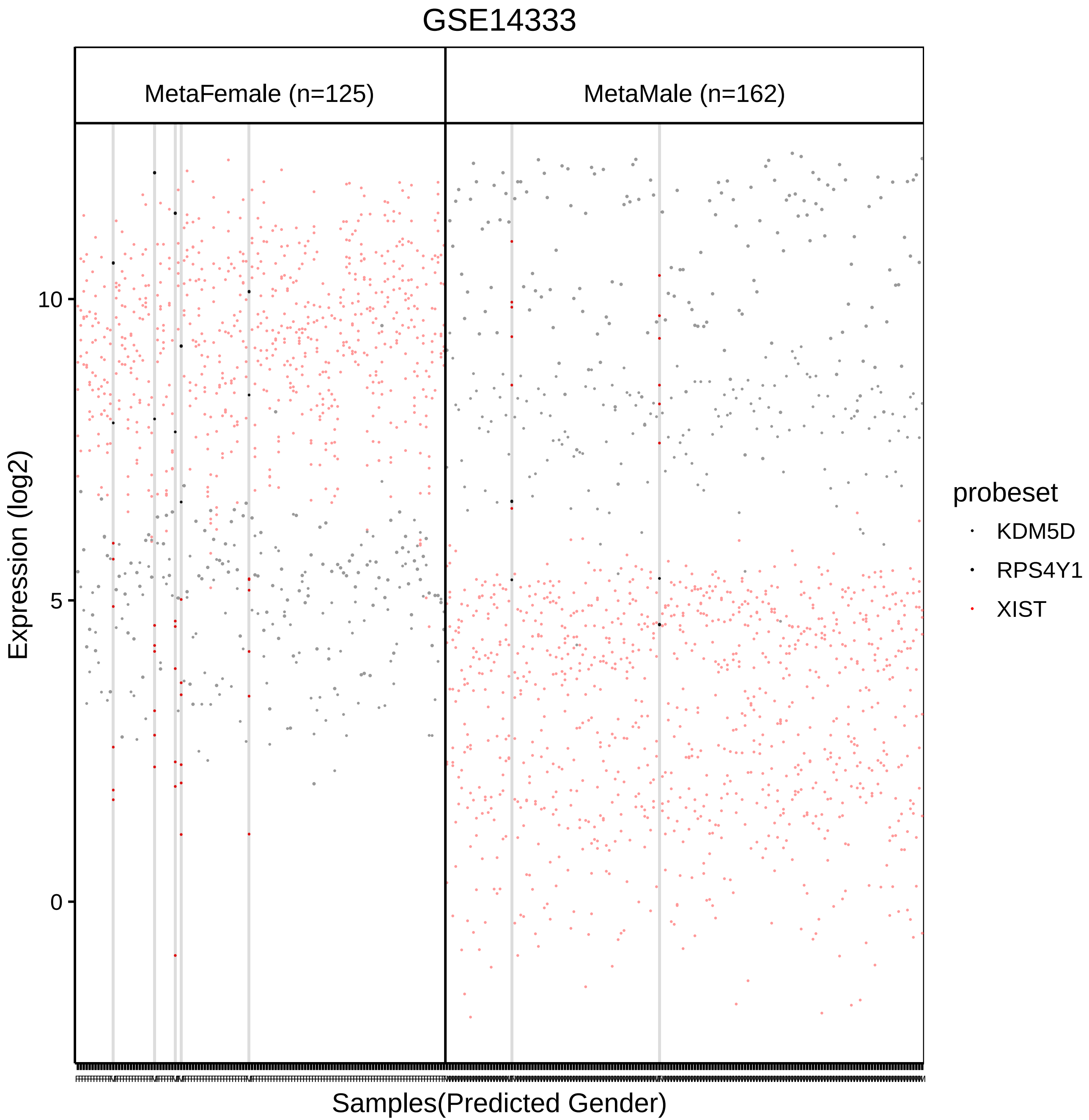
GSE14333
MetaFemale (n=125)
MetaMale (n=162)
Expression (log2)
10
5
0
Samples(Predicted Gender)
probeset
KDM5D
RPS4Y1
XIST

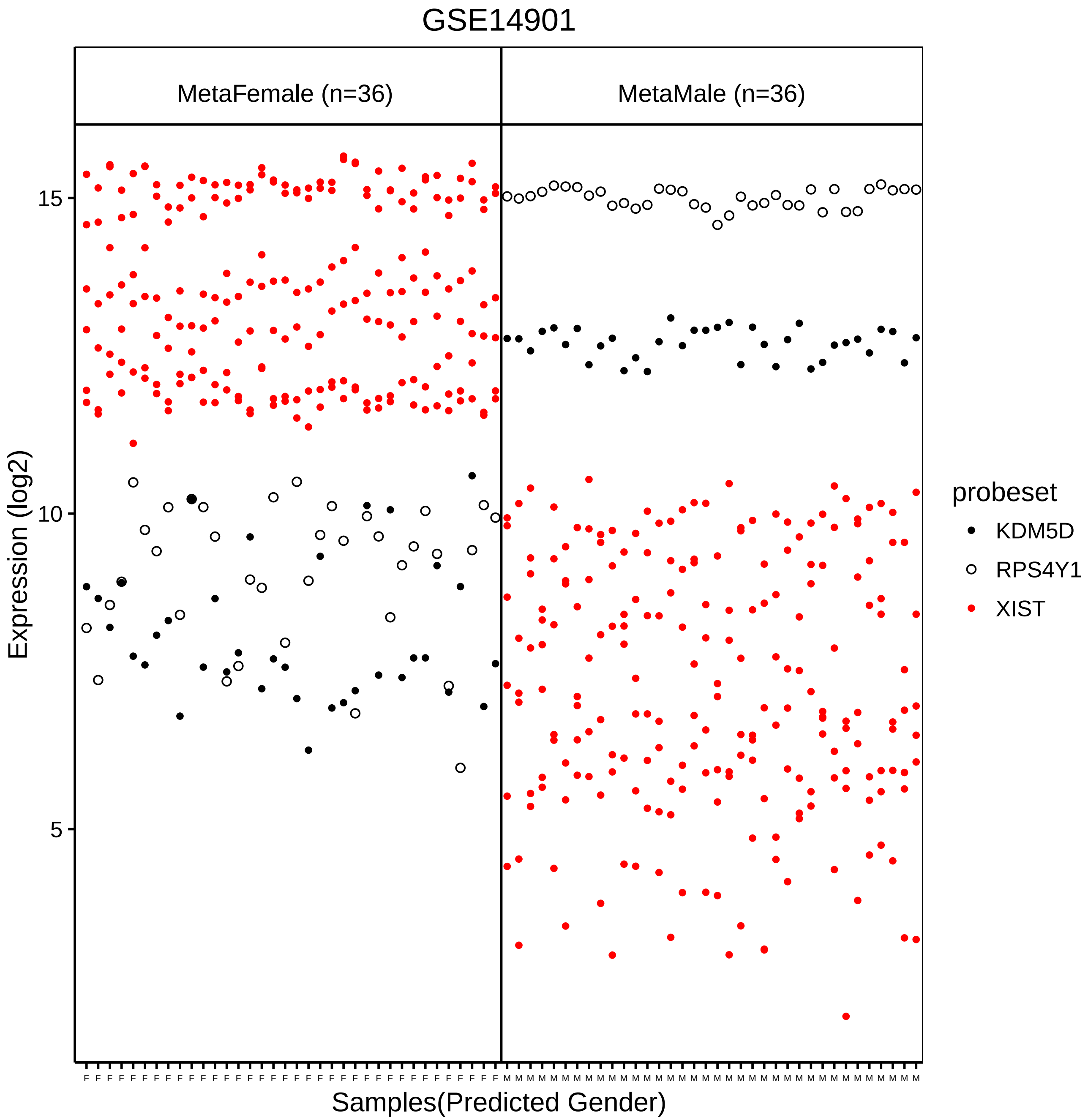
GSE14901
MetaFemale (n=36)
MetaMale (n=36)
Expression (log2)
15
10
5
Samples(Predicted Gender)
probeset
KDM5D
RPS4Y1
XIST

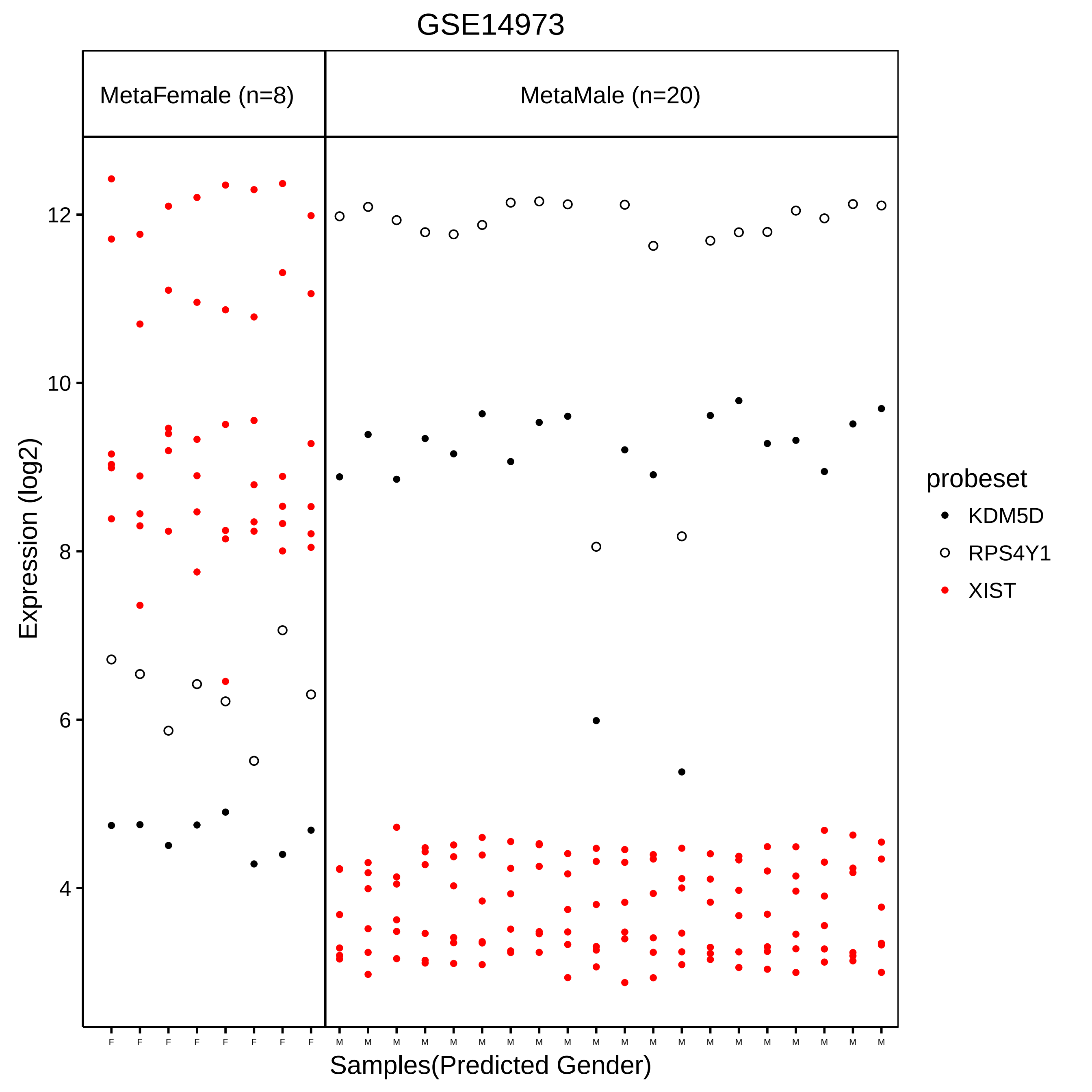
GSE14973
MetaFemale (n=8)
MetaMale (n=20)
Expression (log2)
12
10
8
6
4
F F F F F F F F
M M M M M M M M M M M M M M M M M M M M
Samples(Predicted Gender)
probeset
KDM5D
RPS4Y1
XIST

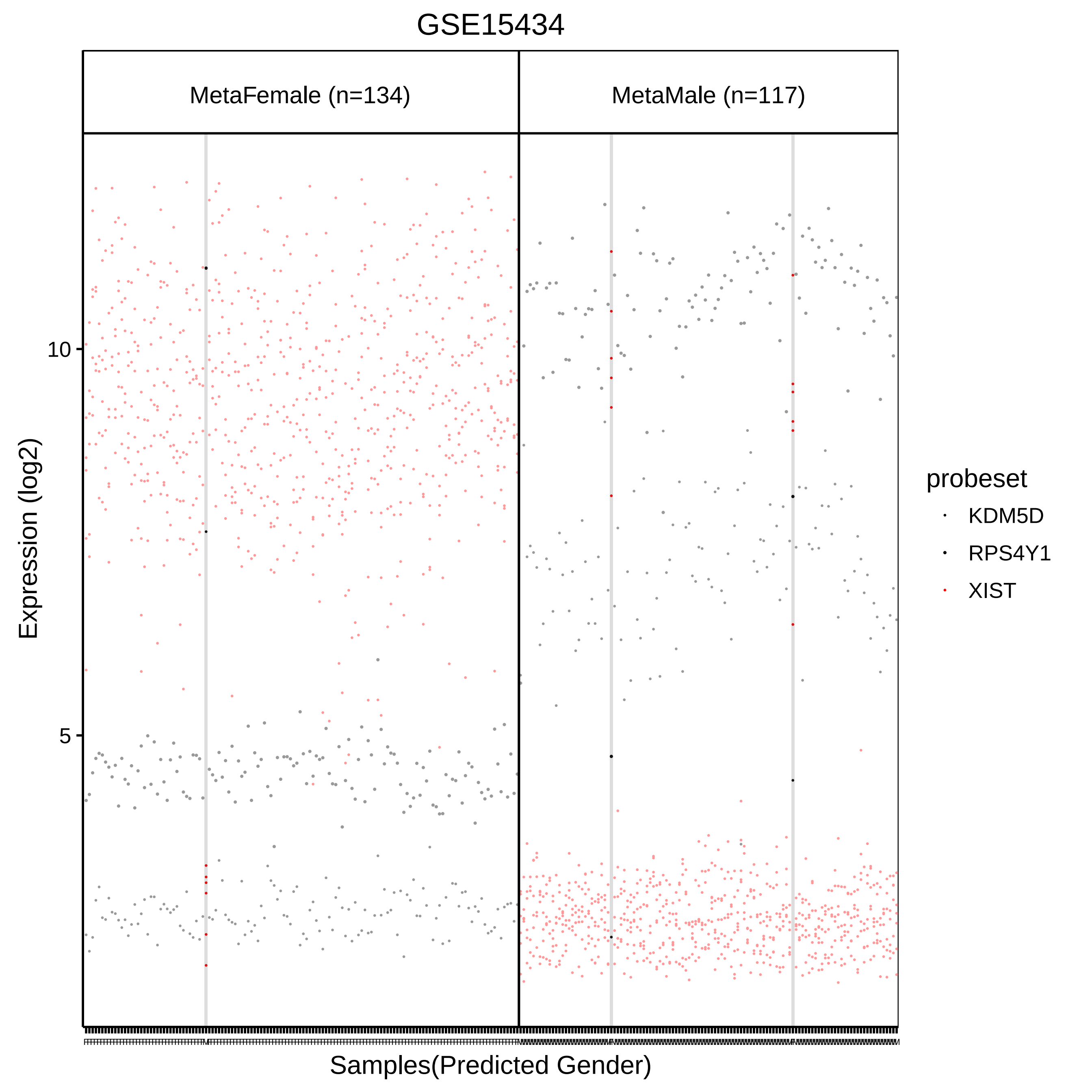

GSE15434
MetaFemale (n=134)
MetaMale (n=117)
10
5
Expression (log2)
Samples(Predicted Gender)
probeset
KDM5D
RPS4Y1
XIST

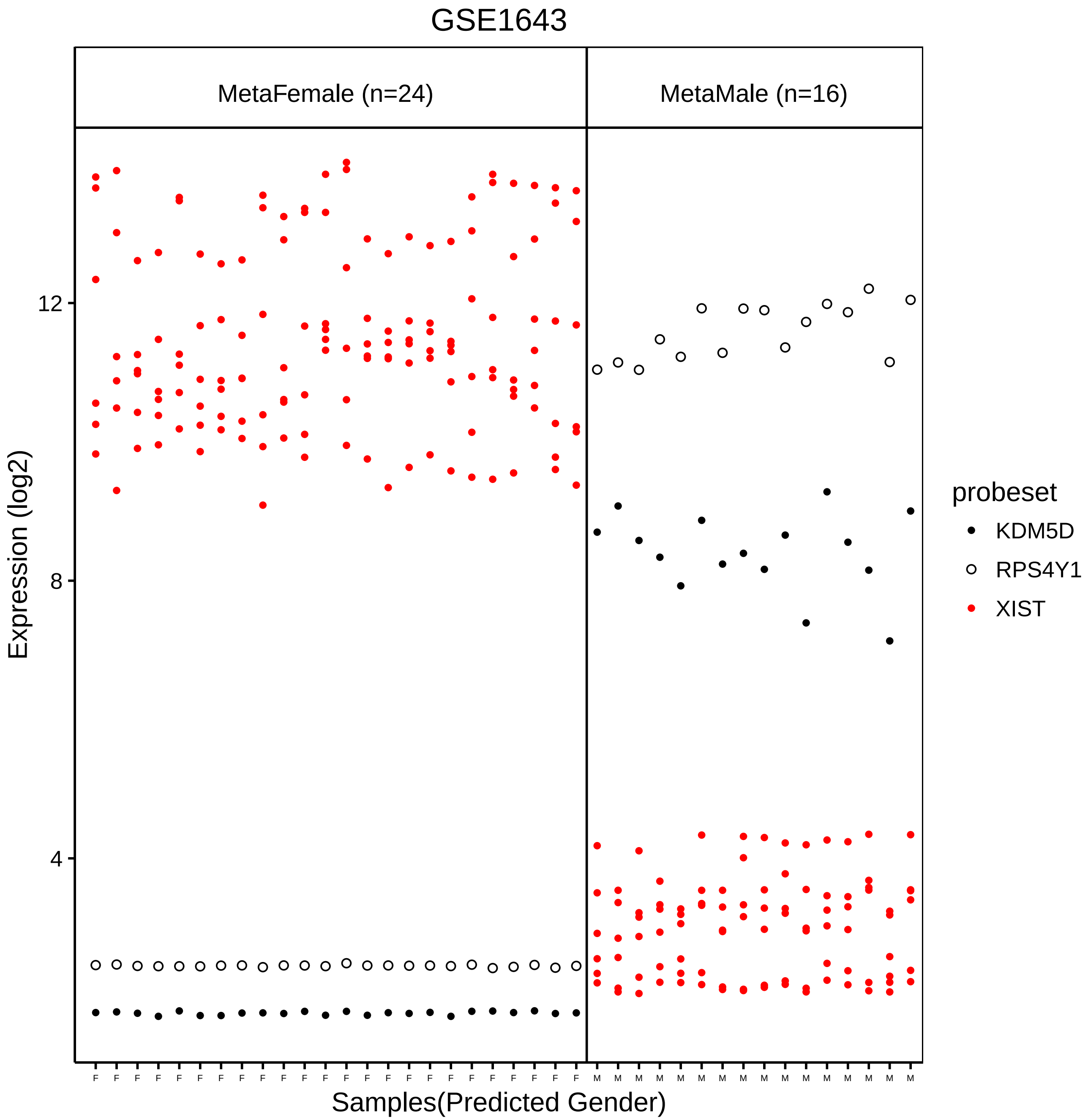
GSE1643
MetaFemale (n=24)
MetaMale (n=16)
Expression (log2)
12
8
4
Samples(Predicted Gender)
F F F F F F F F F F F F F F F F F F F F F F F F
M M M M M M M M M M M M M M M M
probeset
KDM5D
RPS4Y1
XIST

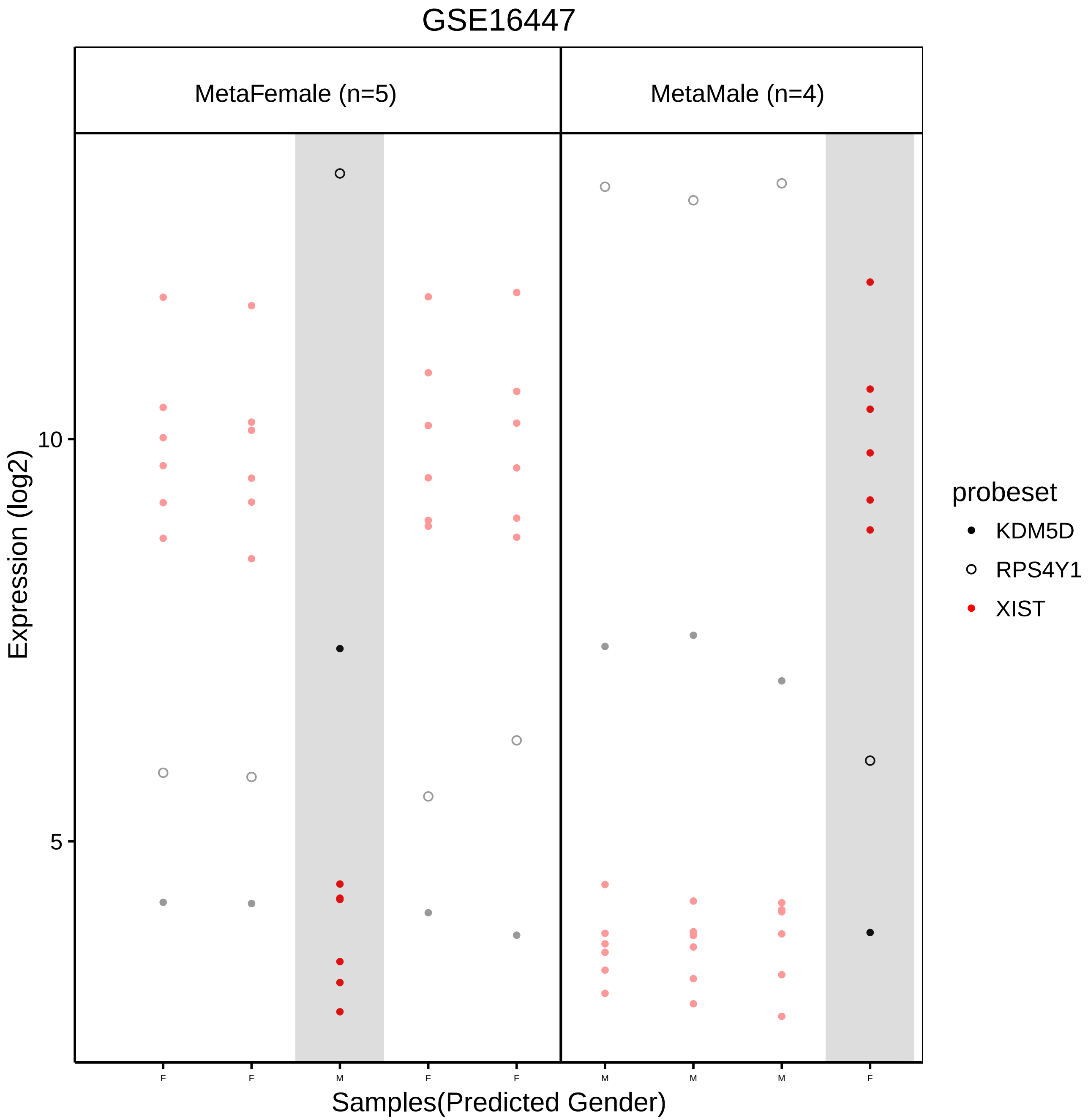
GSE16447
MetaFemale (n=5)
MetaMale (n=4)
Expression (log2)
10
5
F
F
M
F
F
M
M
M
F
Samples(Predicted Gender)
probeset
KDM5D
RPS4Y1
XIST

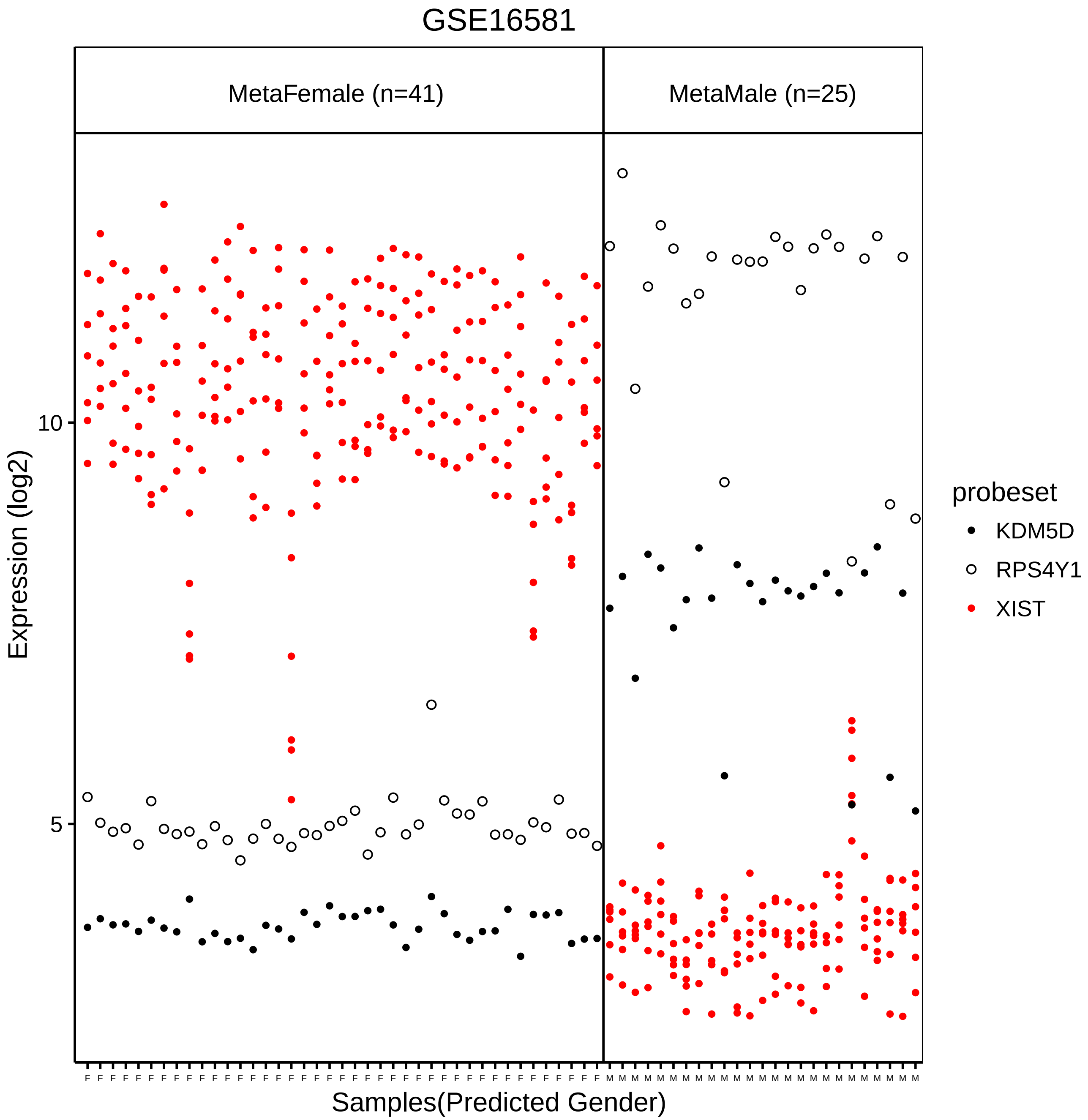

GSE16581
MetaFemale (n=41)
MetaMale (n=25)
Expression (log2)
10
5
Samples(Predicted Gender)
probeset
KDM5D
RPS4Y1
XIST

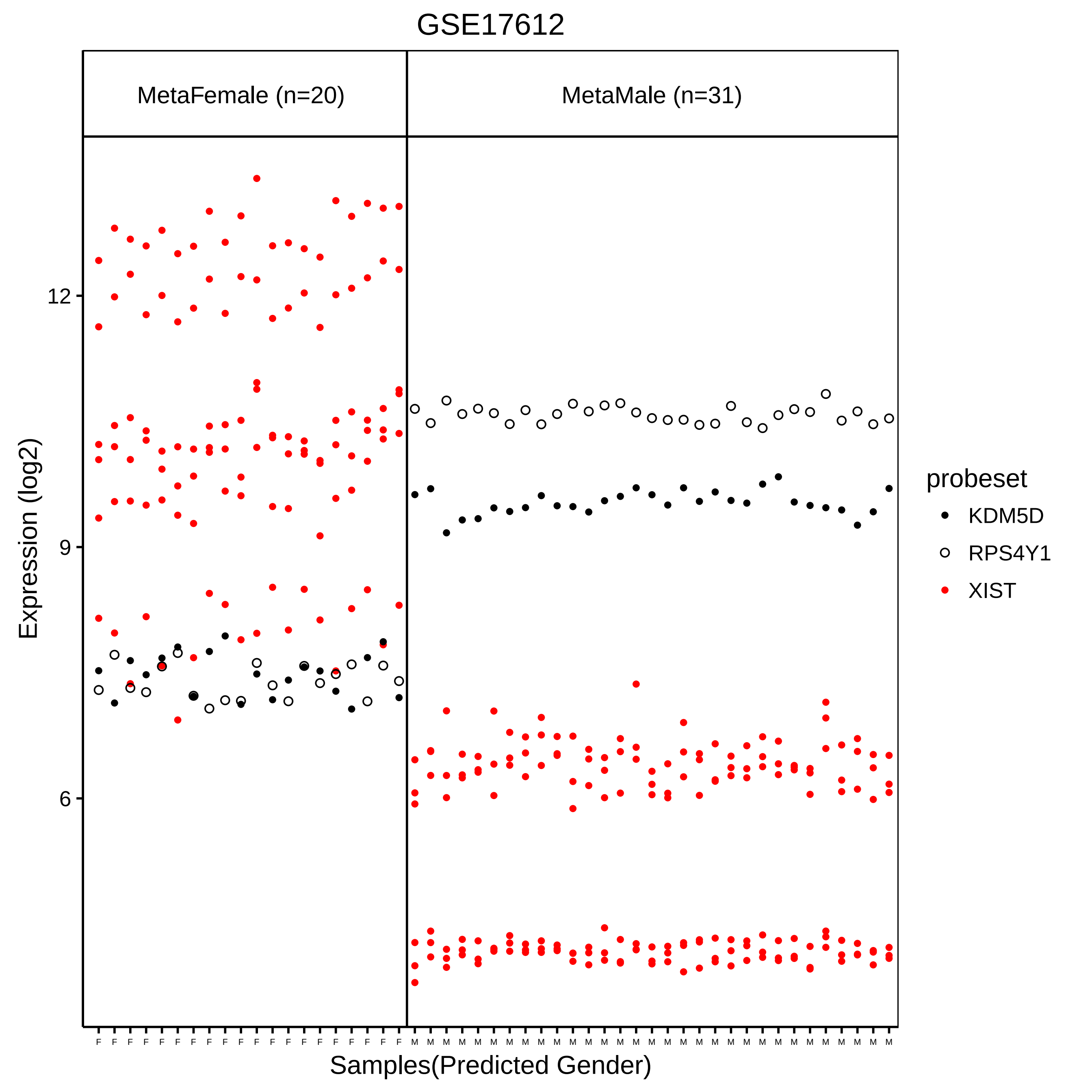
GSE17612
MetaFemale (n=20)
MetaMale (n=31)
Expression (log2)
12
9
6
Samples(Predicted Gender)
probeset
KDM5D
RPS4Y1
XIST

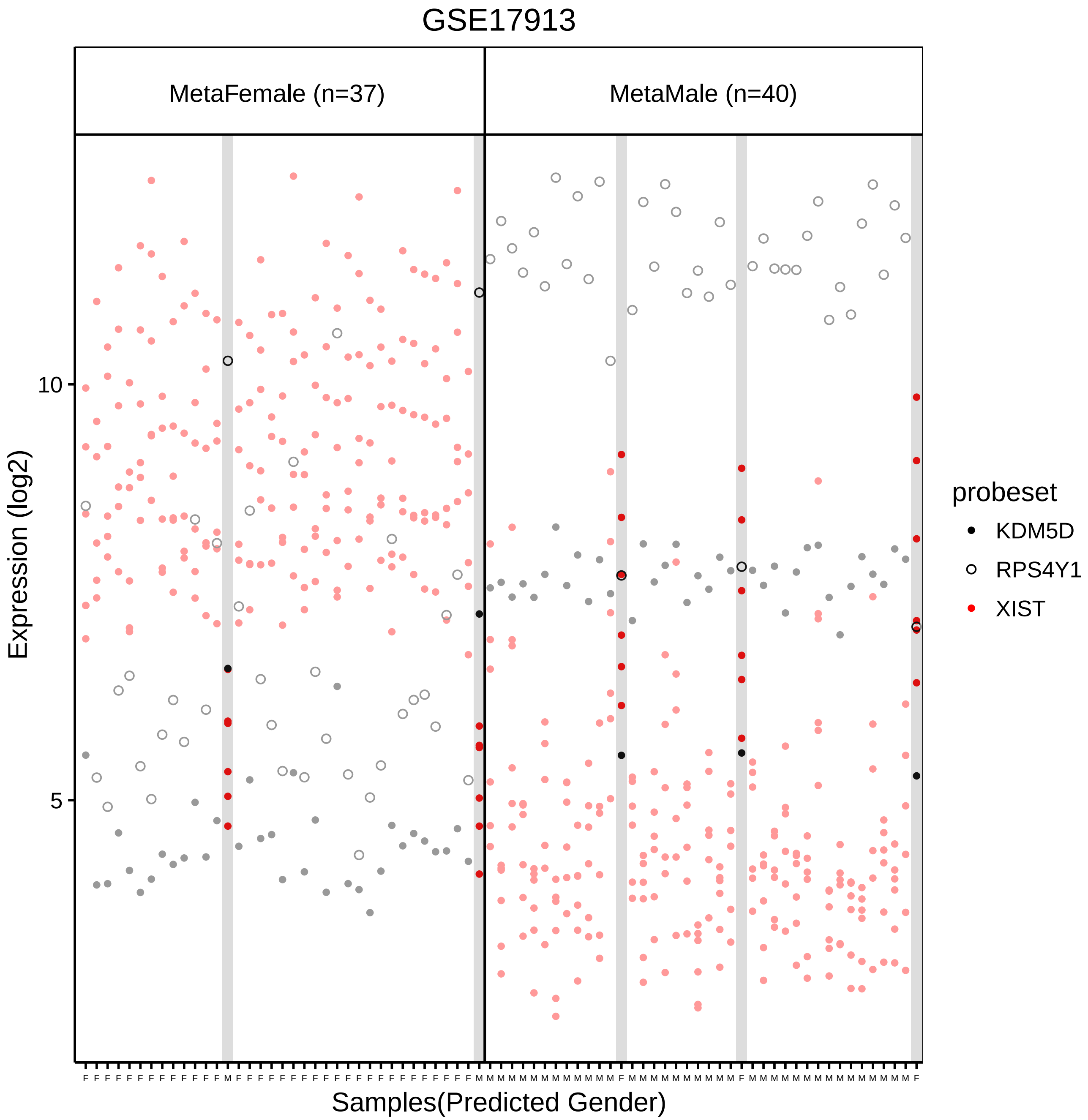
GSE17913
MetaFemale (n=37)
MetaMale (n=40)
Expression (log2)
10
5
Samples(Predicted Gender)
probeset
KDM5D
RPS4Y1
XIST

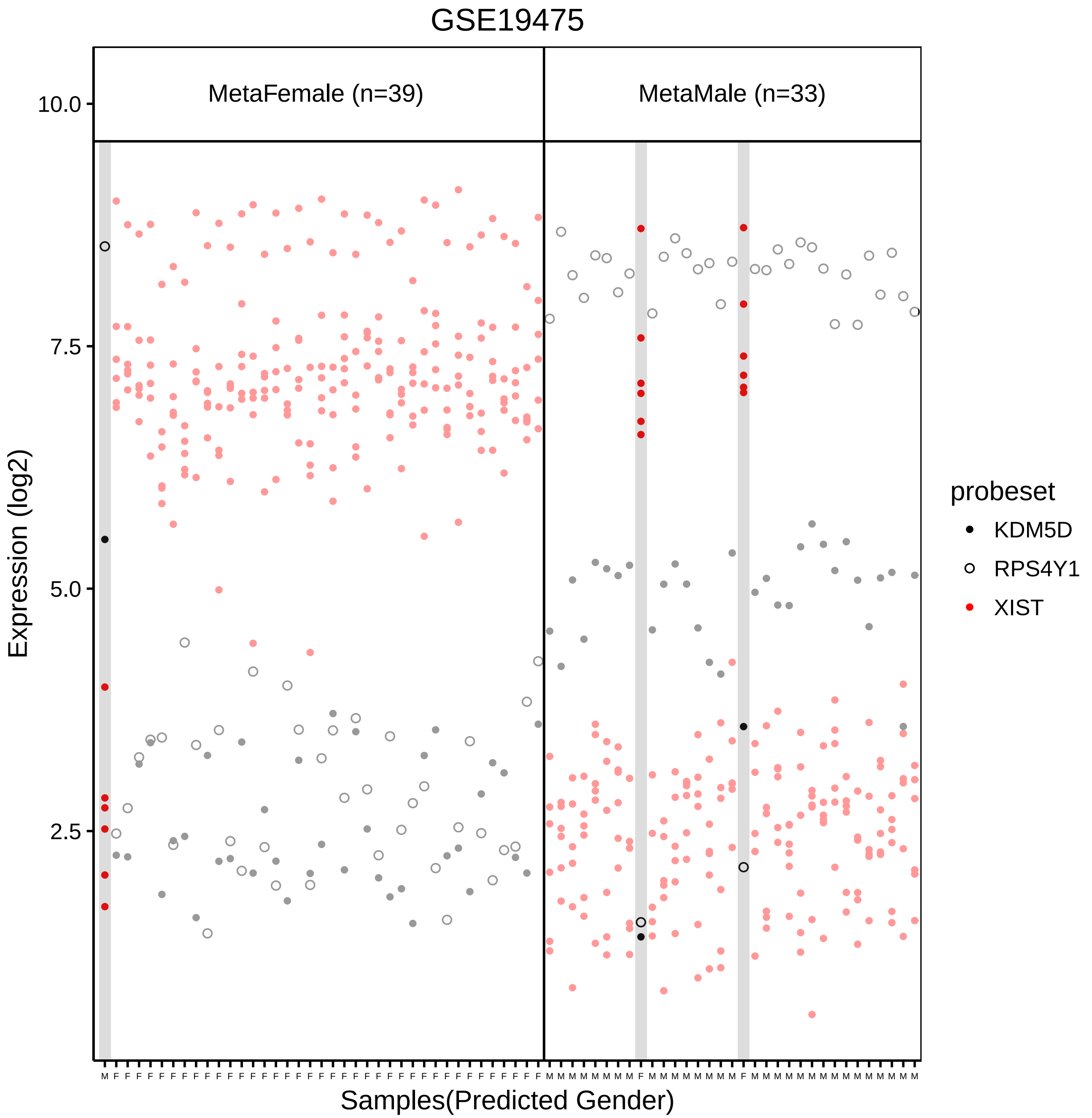

GSE19475
MetaFemale (n=39)
MetaMale (n=33)
Expression (log2)
10.0
7.5
5.0
2.5
Samples(Predicted Gender)
probeset
KDM5D
RPS4Y1
XIST

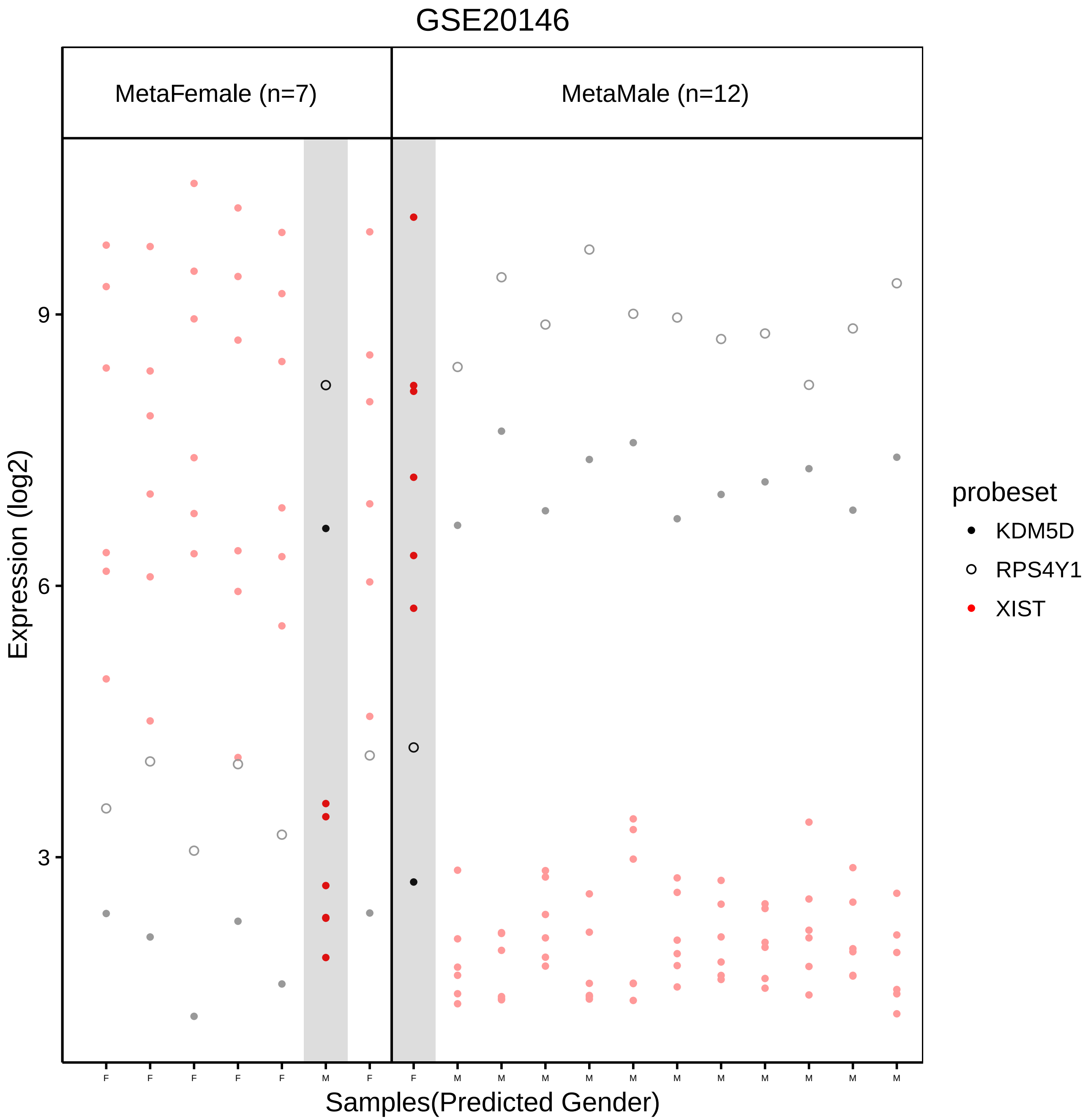
GSE20146
MetaFemale (n=7)
MetaMale (n=12)
Expression (log2)
9
6
3
F
F
F
F
F
M
F
F
M
M
M
M
M
M
M
M
M
M
M
Samples(Predicted Gender)
probeset
KDM5D
RPS4Y1
XIST

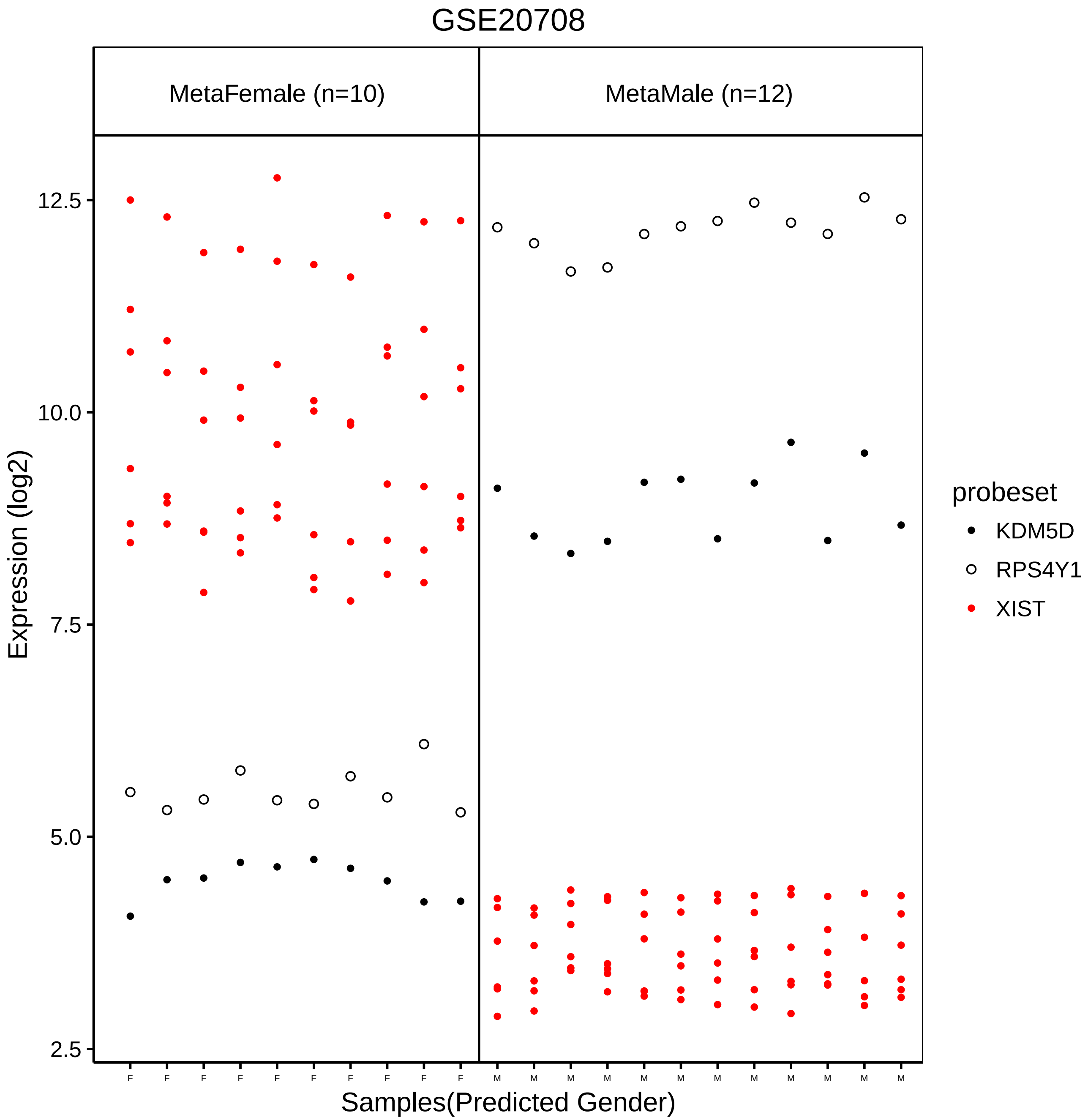
GSE20708
MetaFemale (n=10)
MetaMale (n=12)
Expression (log2)
12.5
10.0
7.5
5.0
2.5
F F F F F F F F F F
M M M M M M M M M M M M
Samples(Predicted Gender)
probeset
KDM5D
RPS4Y1
XIST

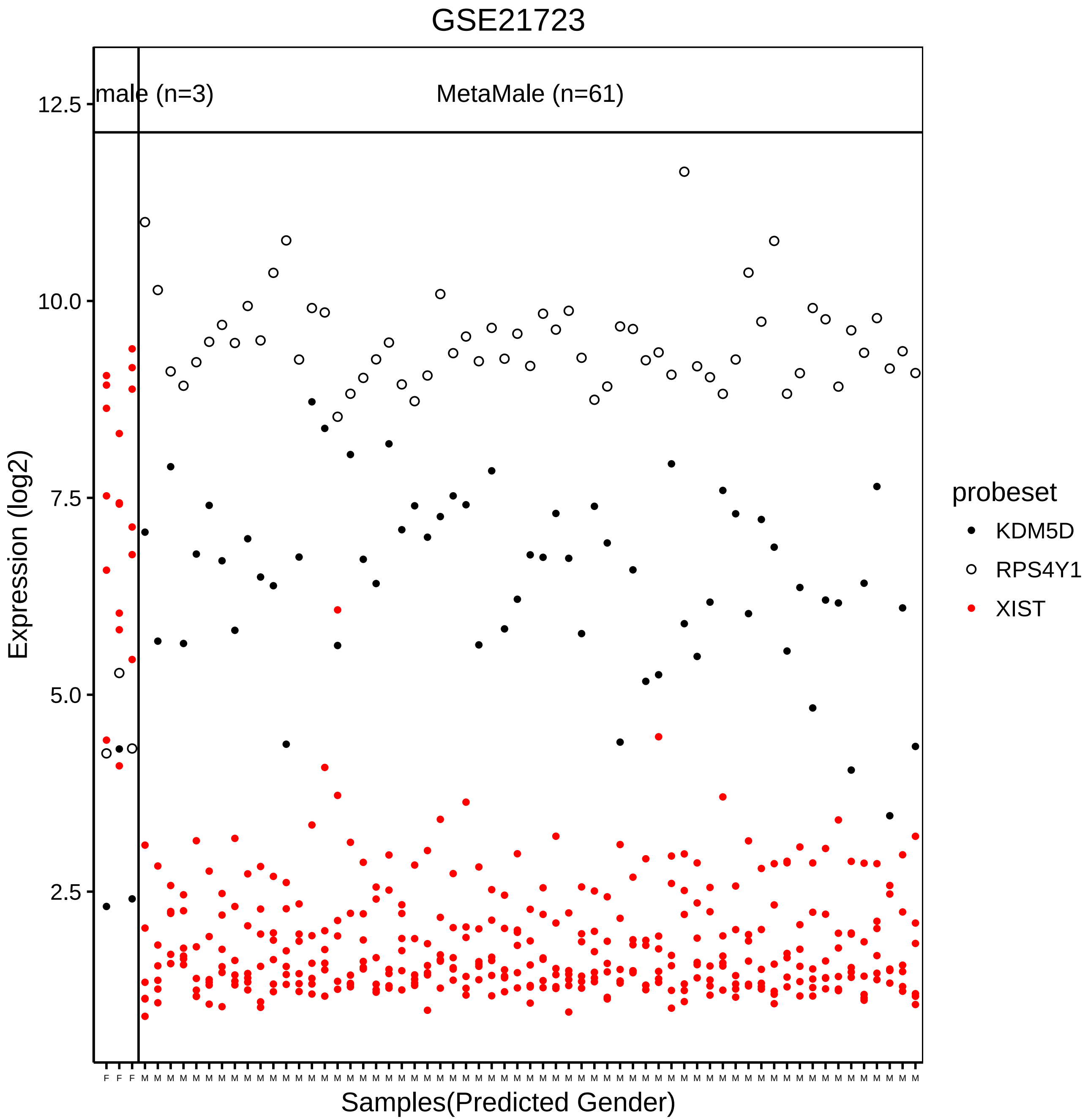

GSE21723
male (n=3)
MetaMale (n=61)
Expression (log2)
12.5
10.0
7.5
5.0
2.5
Samples(Predicted Gender)
F F F M M M M M M M M M M M M M M M M M M M M M M M M M M M M M M M M M M M M M M M M M M M M M M M M M M M M M M M M M M M M M
probeset
KDM5D
RPS4Y1
XIST

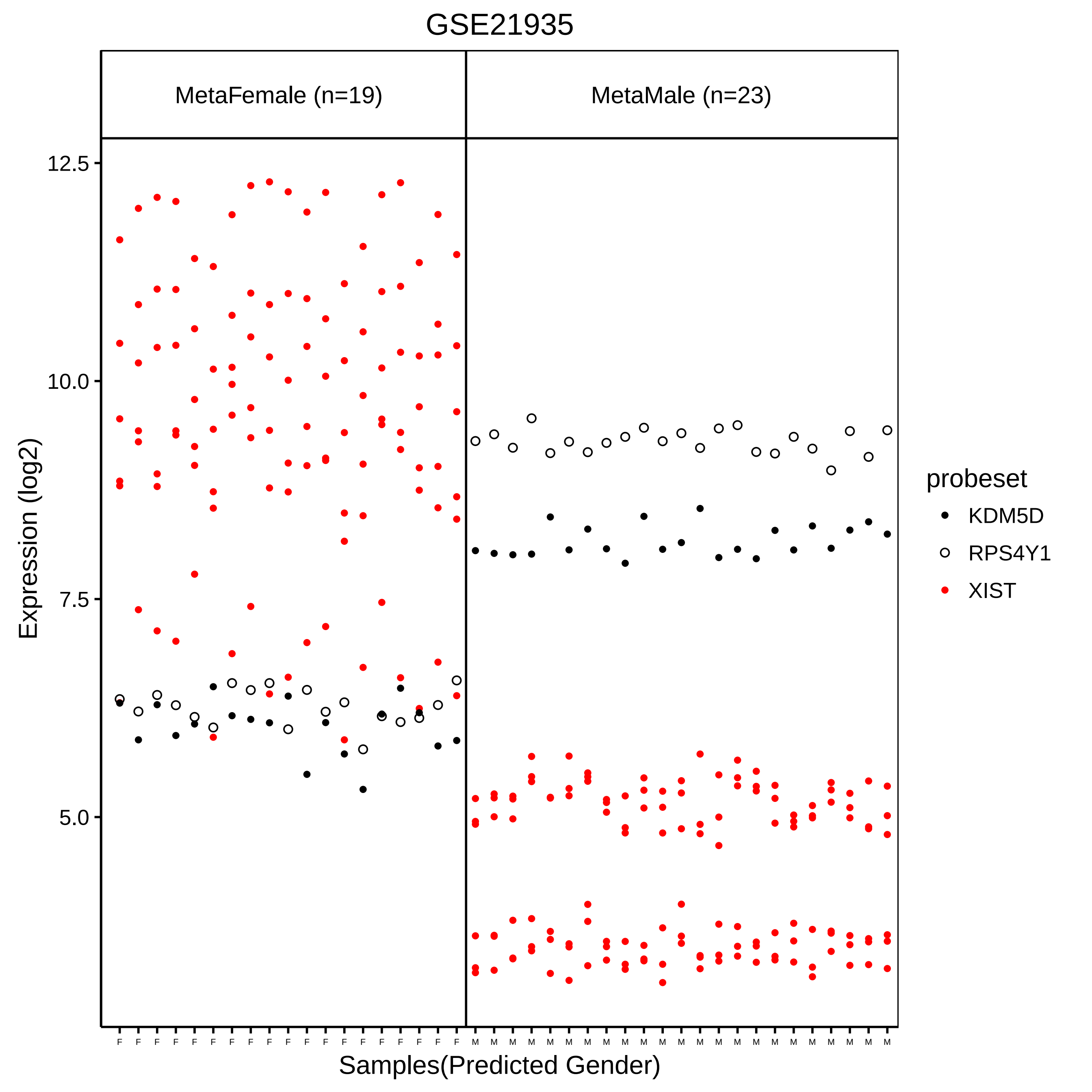
GSE21935
MetaFemale (n=19)
MetaMale (n=23)
12.5
10.0
7.5
5.0
Expression (log2)
F F F F F F F F F F F F F F F F F F F
M M M M M M M M M M M M M M M M M M M M M M M
Samples(Predicted Gender)
probeset
KDM5D
RPS4Y1
XIST

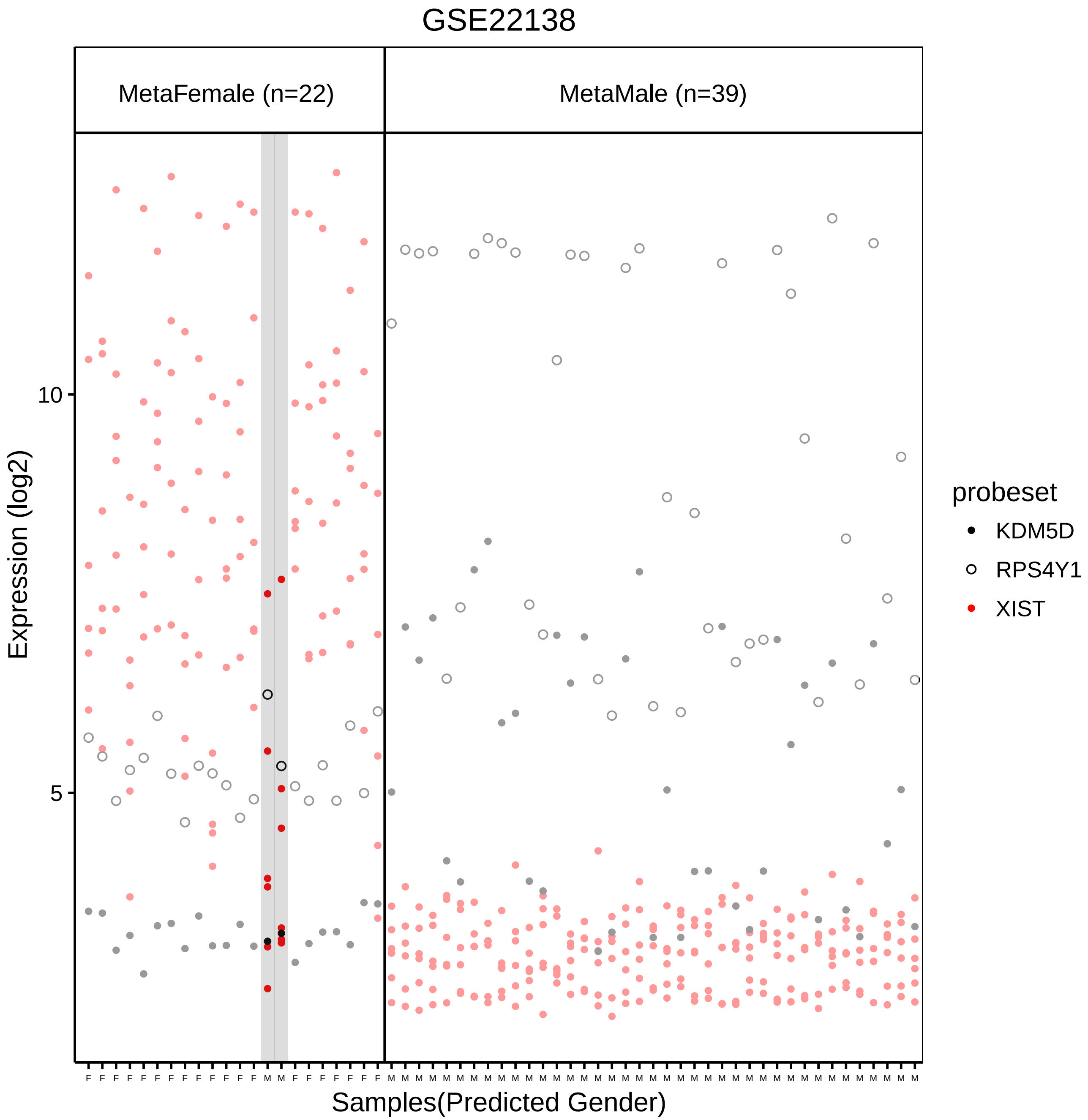

GSE22138
MetaFemale (n=22)
MetaMale (n=39)
Expression (log2)
10
5
Samples(Predicted Gender)
probeset
KDM5D
RPS4Y1
XIST

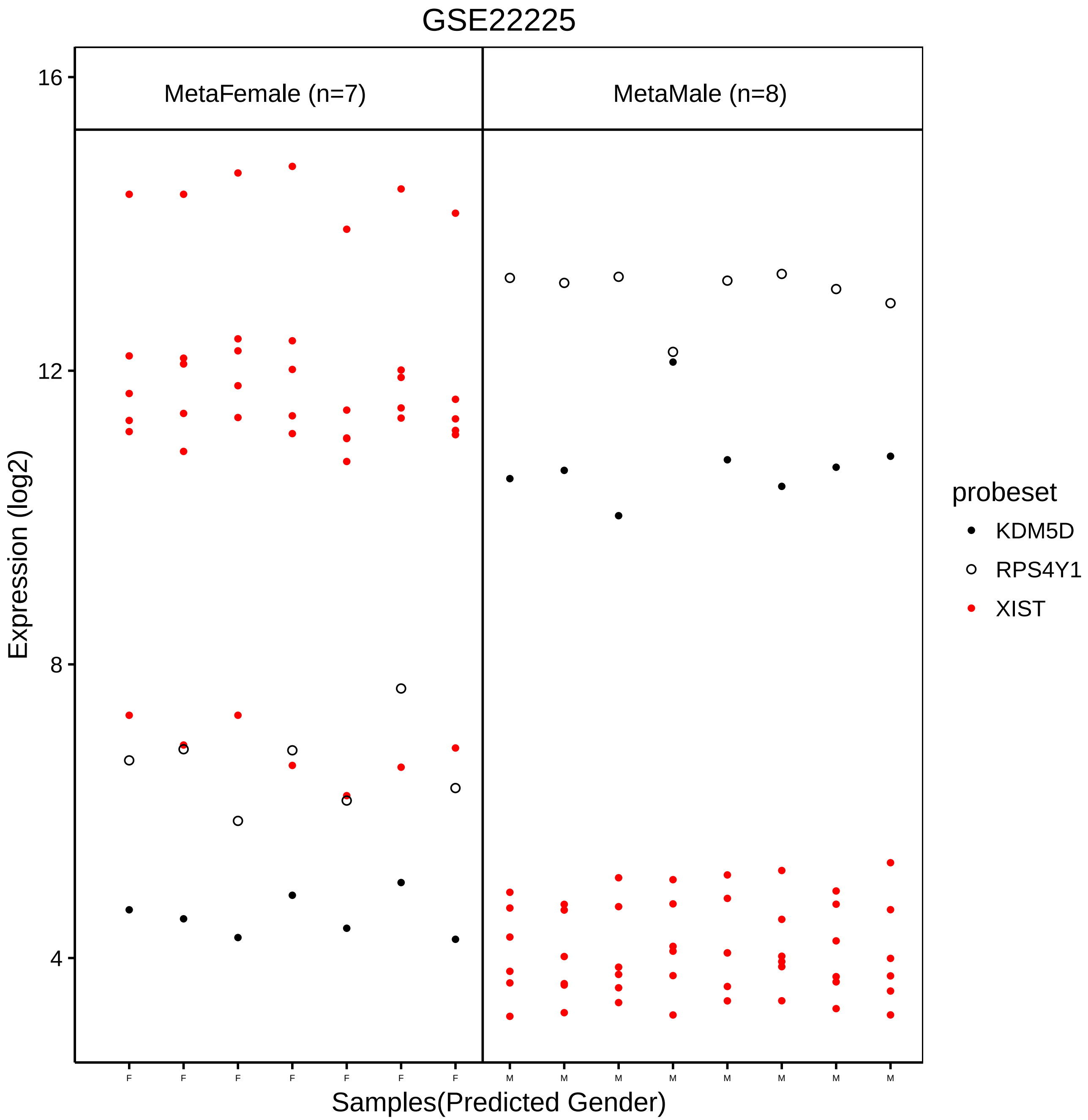

GSE22225
MetaFemale (n=7)
MetaMale (n=8)
16
12
8
4
Expression (log2)
F
M
Samples(Predicted Gender)
probeset
KDM5D
RPS4Y1
XIST

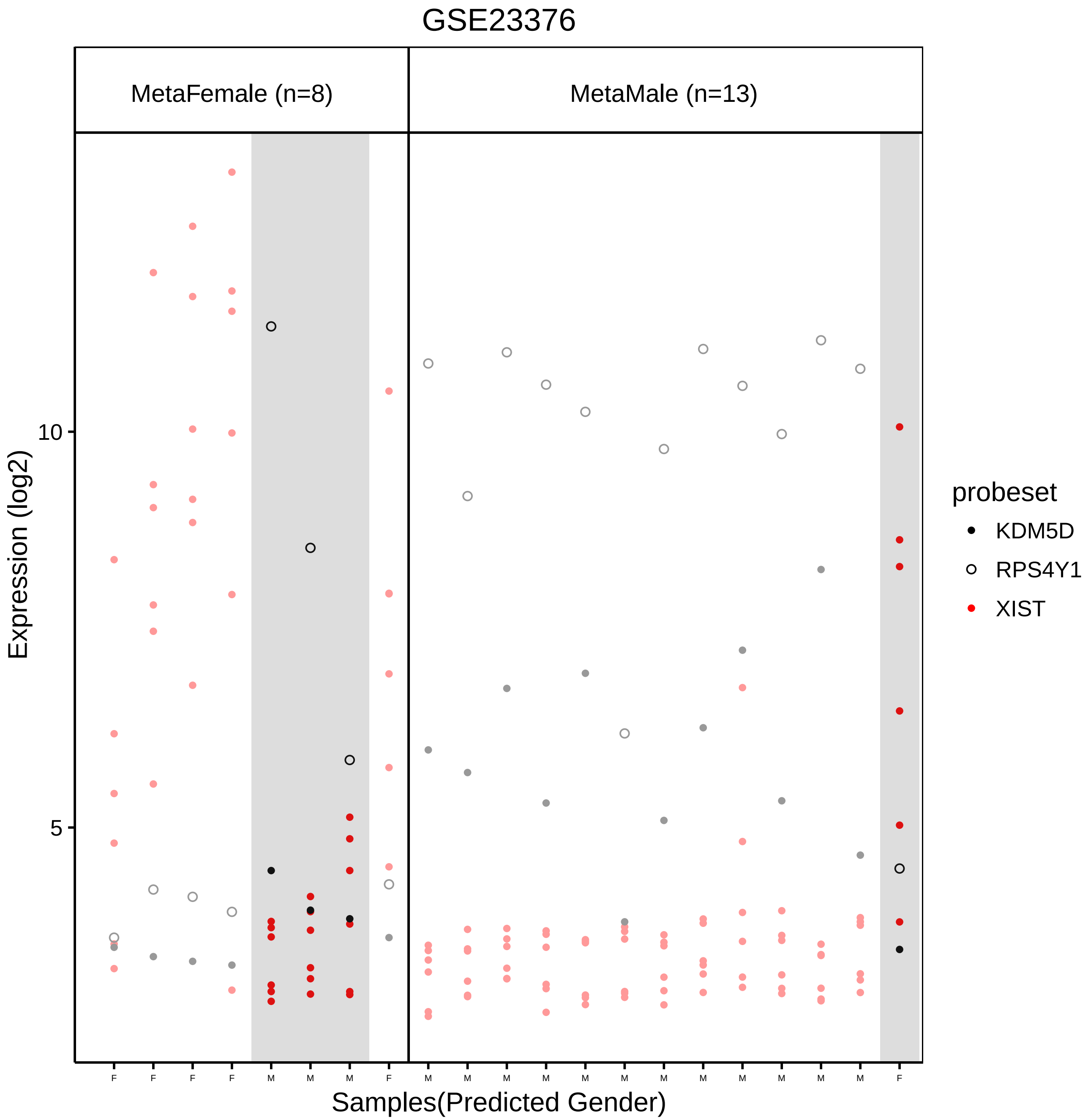

GSE23376
MetaFemale (n=8)
MetaMale (n=13)
Expression (log2)
10
5
F F F F M M M F M M M M M M M M M M M M F
Samples(Predicted Gender)
probeset
KDM5D
RPS4Y1
XIST

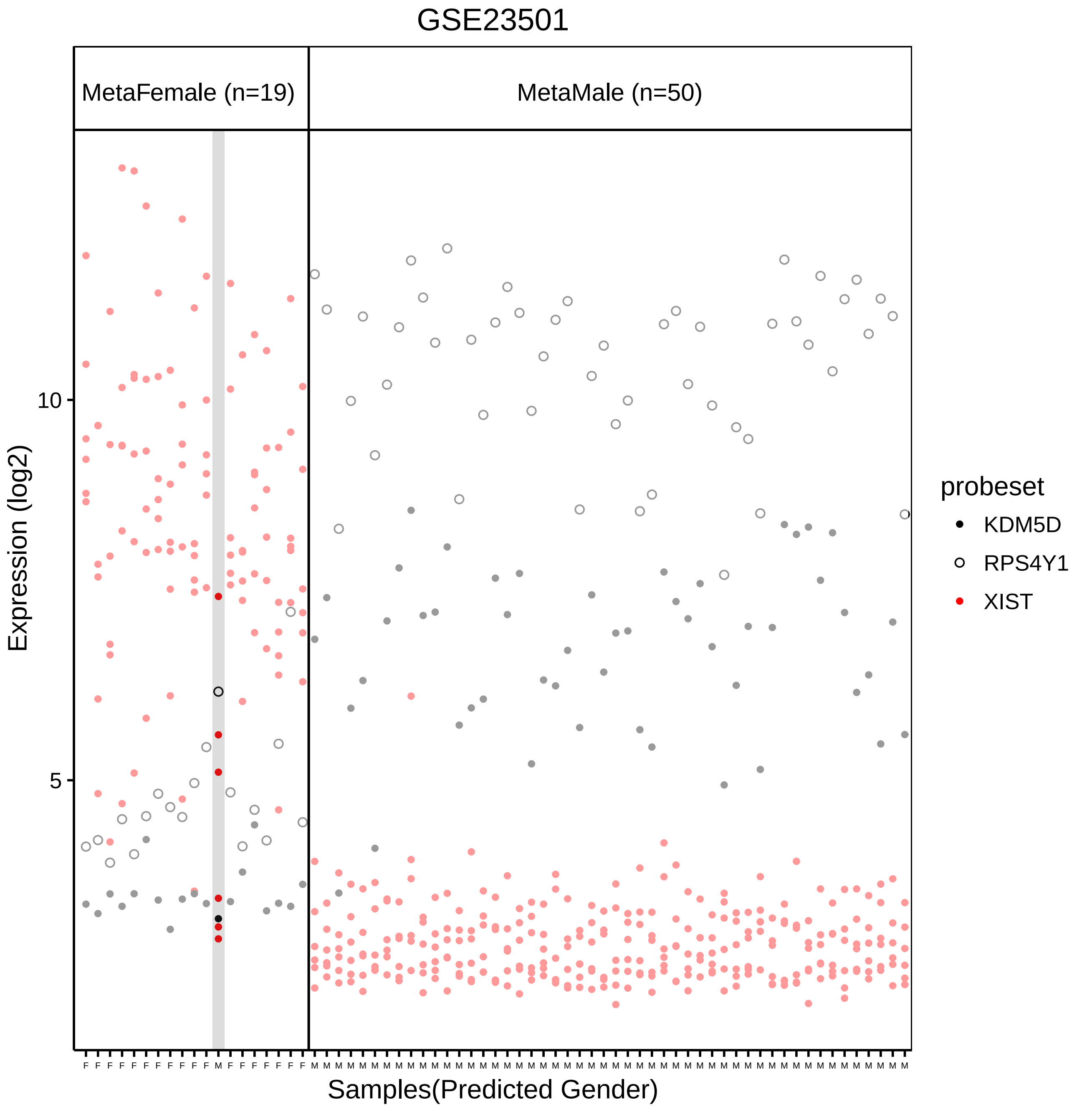

GSE23501
MetaFemale (n=19)
MetaMale (n=50)
Expression (log2)
10
5
Samples(Predicted Gender)
probeset
KDM5D
RPS4Y1
XIST

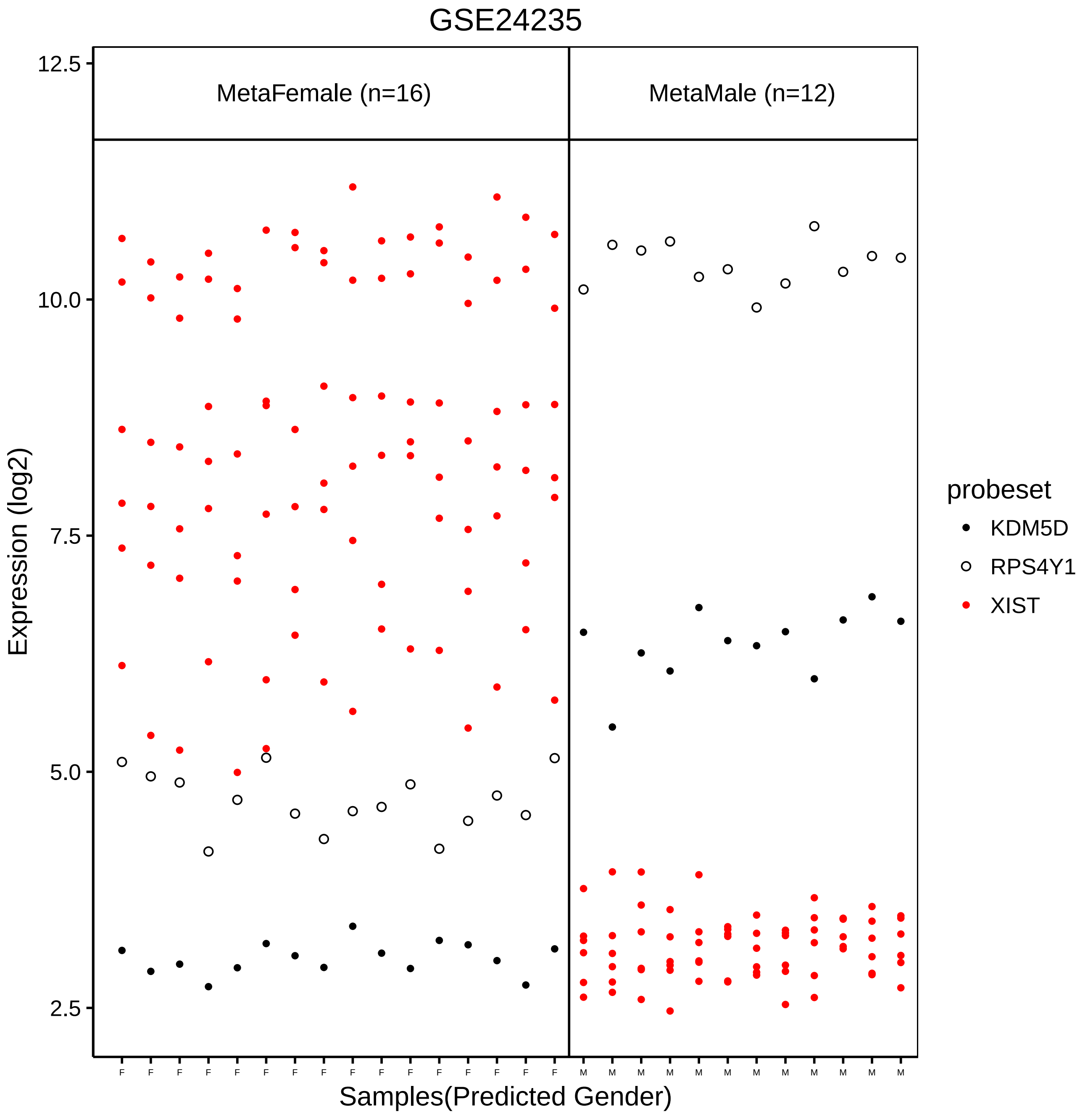

GSE24235
MetaFemale (n=16)
MetaMale (n=12)
12.5
10.0
7.5
5.0
2.5
Expression (log2)
Samples(Predicted Gender)
F F F F F F F F F F F F F F F F
M M M M M M M M M M M M
probeset
KDM5D
RPS4Y1
XIST

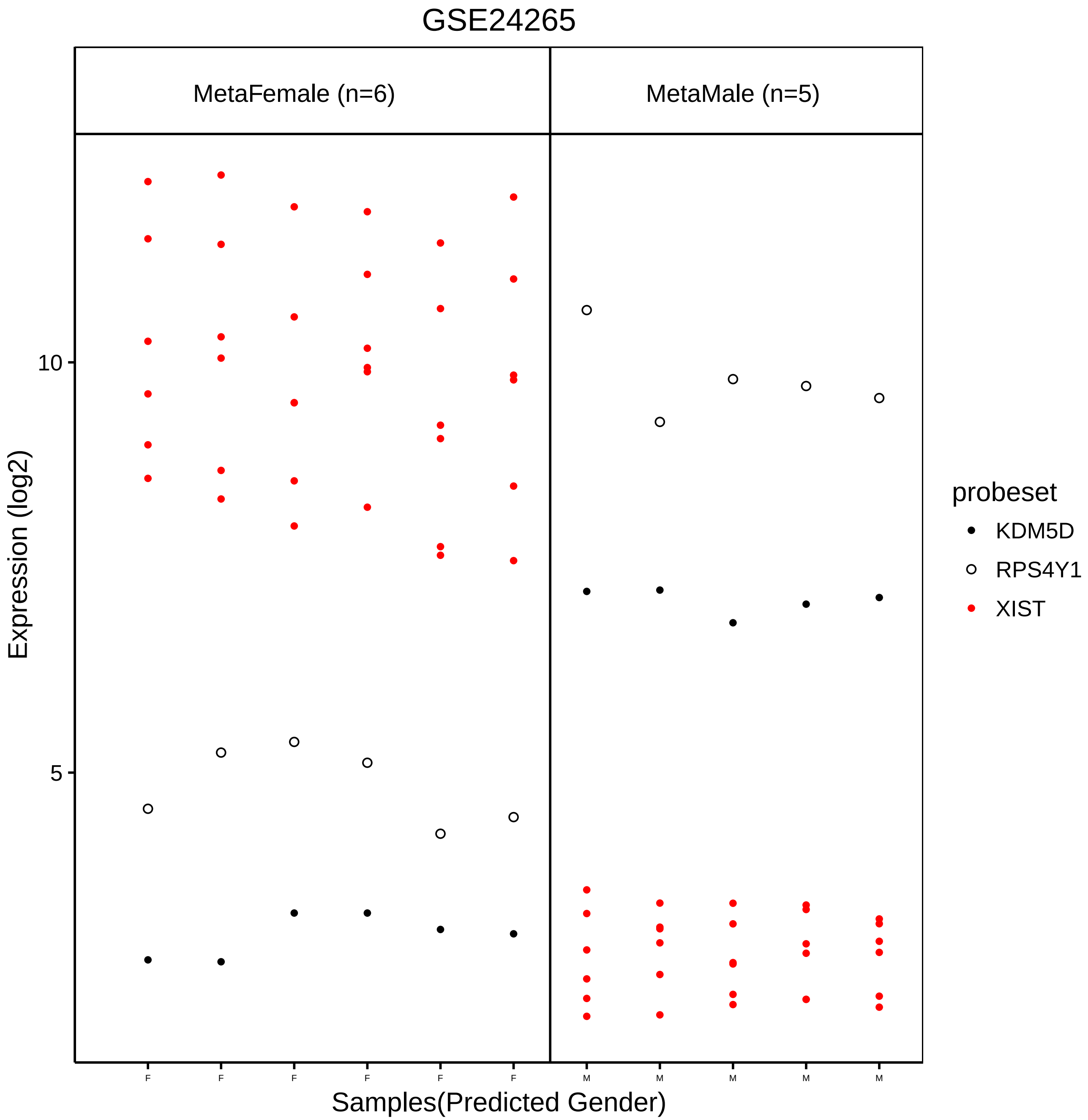

GSE24265
MetaFemale (n=6)
MetaMale (n=5)
10
5
Expression (log2)
probeset
KDM5D
RPS4Y1
XIST
F
F
F
F
F
F
M
M
M
M
M
Samples(Predicted Gender)

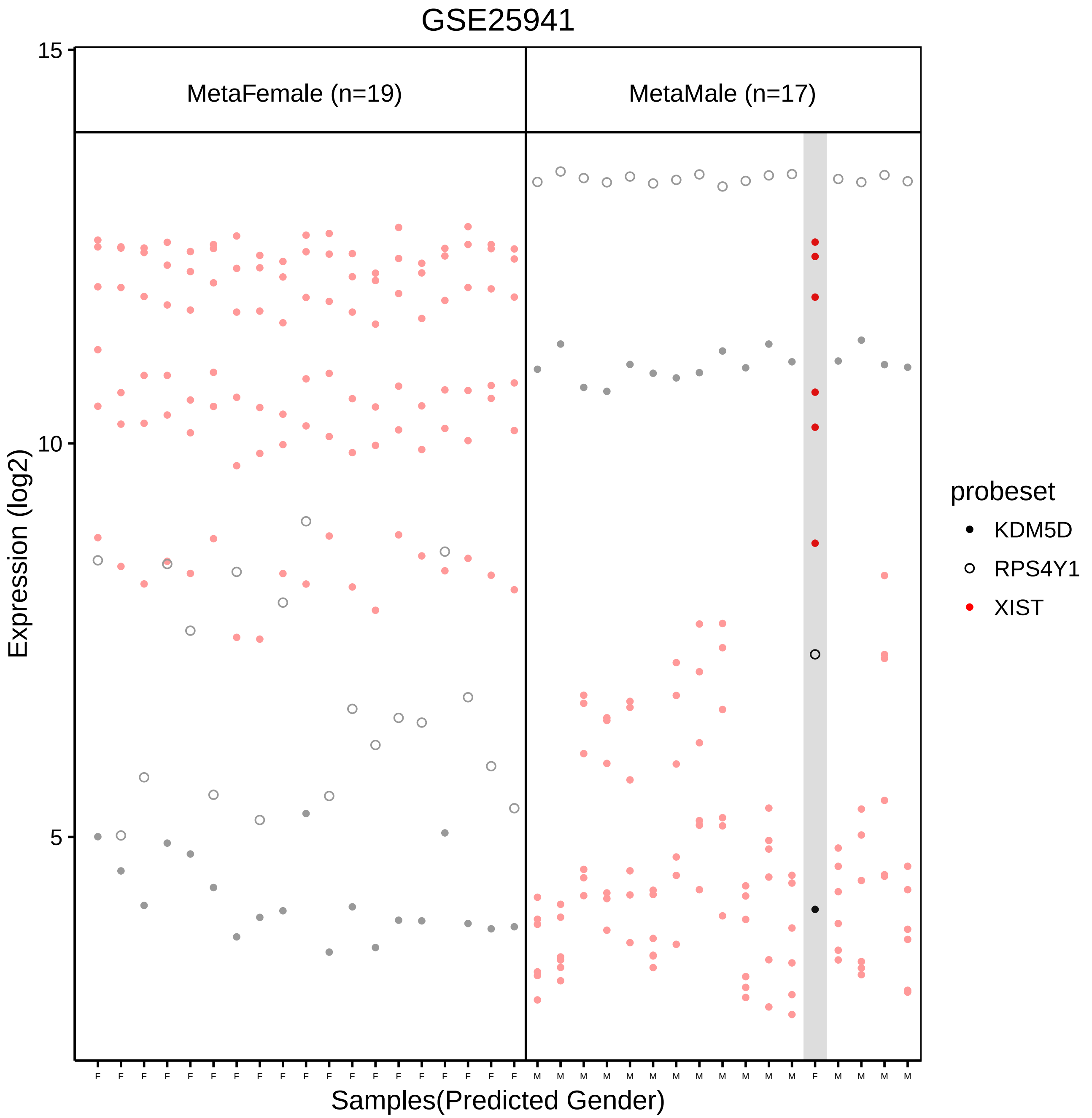

GSE25941
MetaFemale (n=19)
MetaMale (n=17)
15
10
5
Expression (log2)
Samples(Predicted Gender)
F F F F F F F F F F F F F F F F F F F
M M M M M M M M M M M F M M M M
probeset
KDM5D
RPS4Y1
XIST

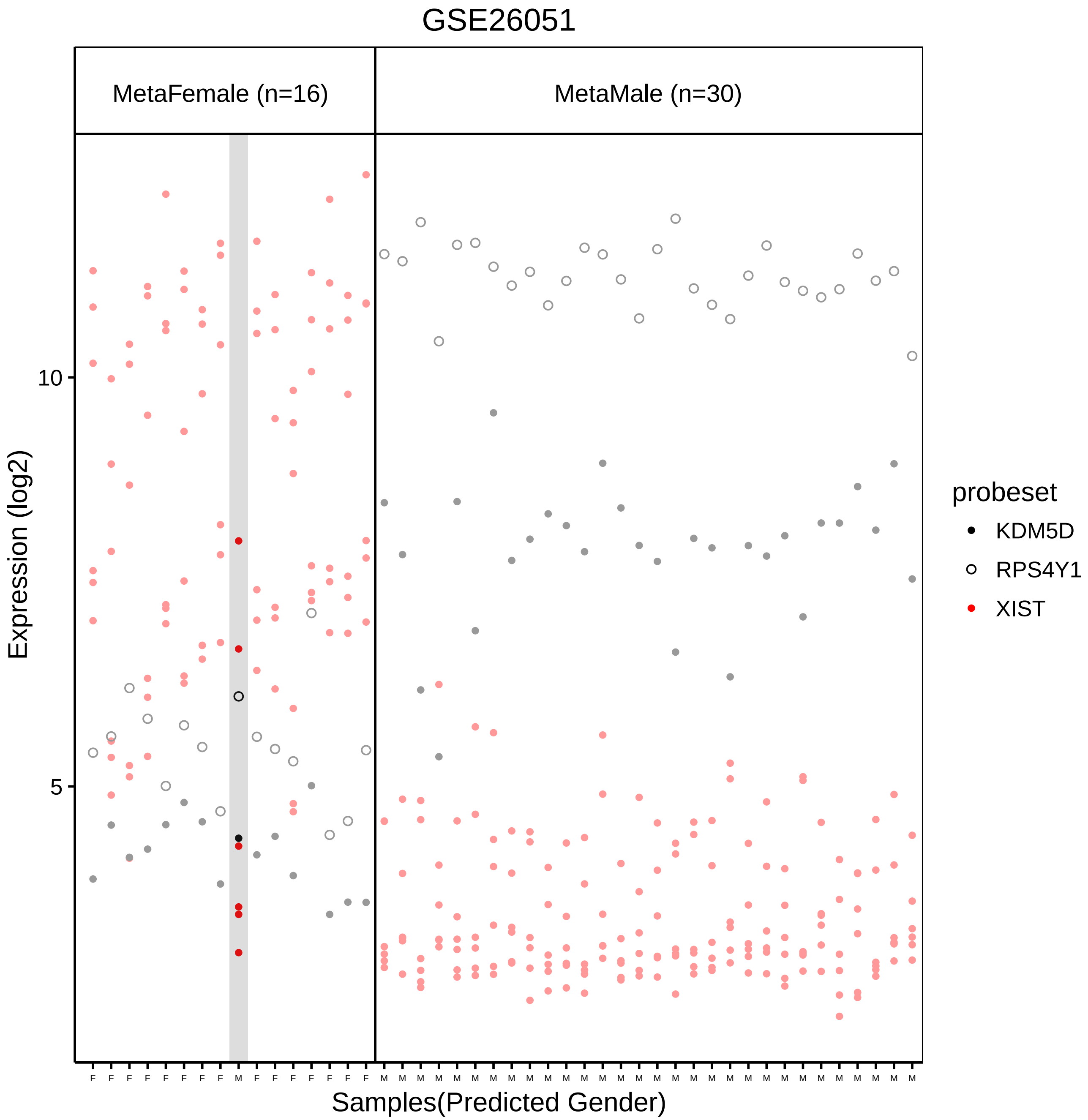
GSE26051
MetaFemale (n=16)
MetaMale (n=30)
10
5
Expression (log2)
Samples(Predicted Gender)
F F F F F F F F M F F F F F F F
M M M M M M M M M M M M M M M M M M M M M M M M M M M M M M
probeset
KDM5D
RPS4Y1
XIST

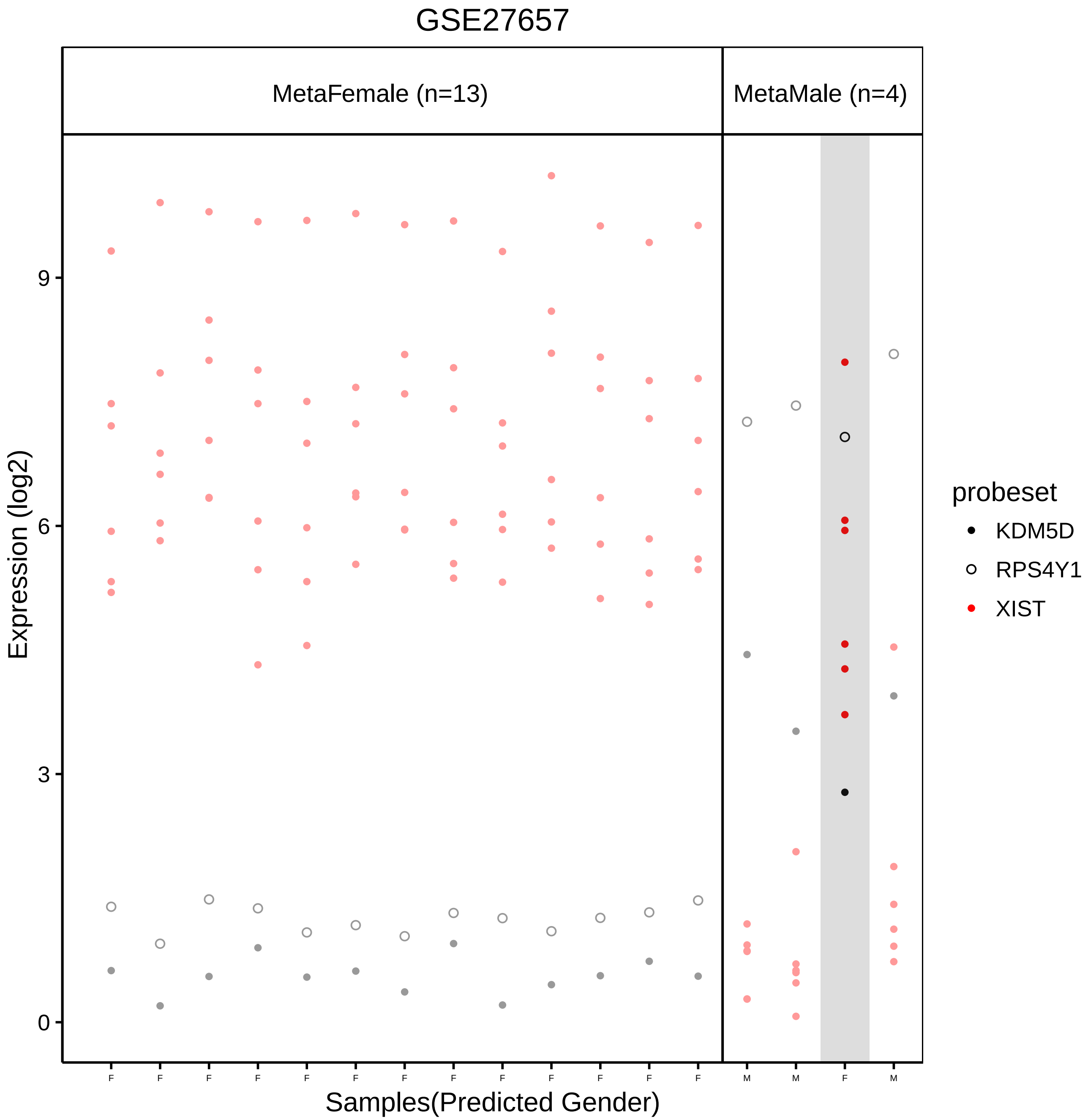

GSE27657
MetaFemale (n=13)
MetaMale (n=4)
Expression (log2)
9
6
3
0
F
F
F
F
F
F
F
F
F
F
F
F
F
M
M
F
M
Samples(Predicted Gender)
probeset
KDM5D
RPS4Y1
XIST

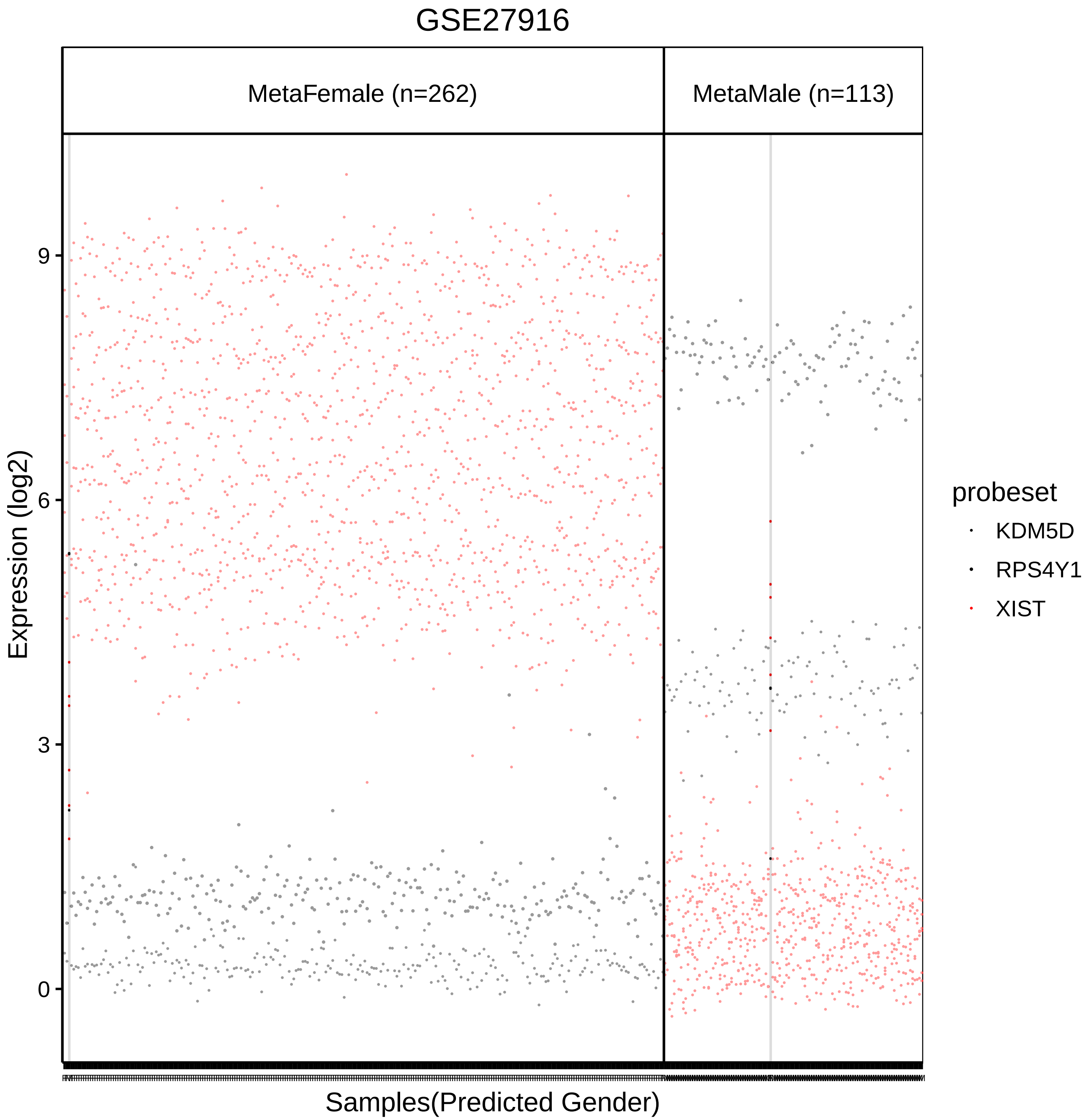
GSE27916
MetaFemale (n=262)
MetaMale (n=113)
Expression (log2)
9
6
3
0
Samples(Predicted Gender)
probeset
KDM5D
RPS4Y1
XIST

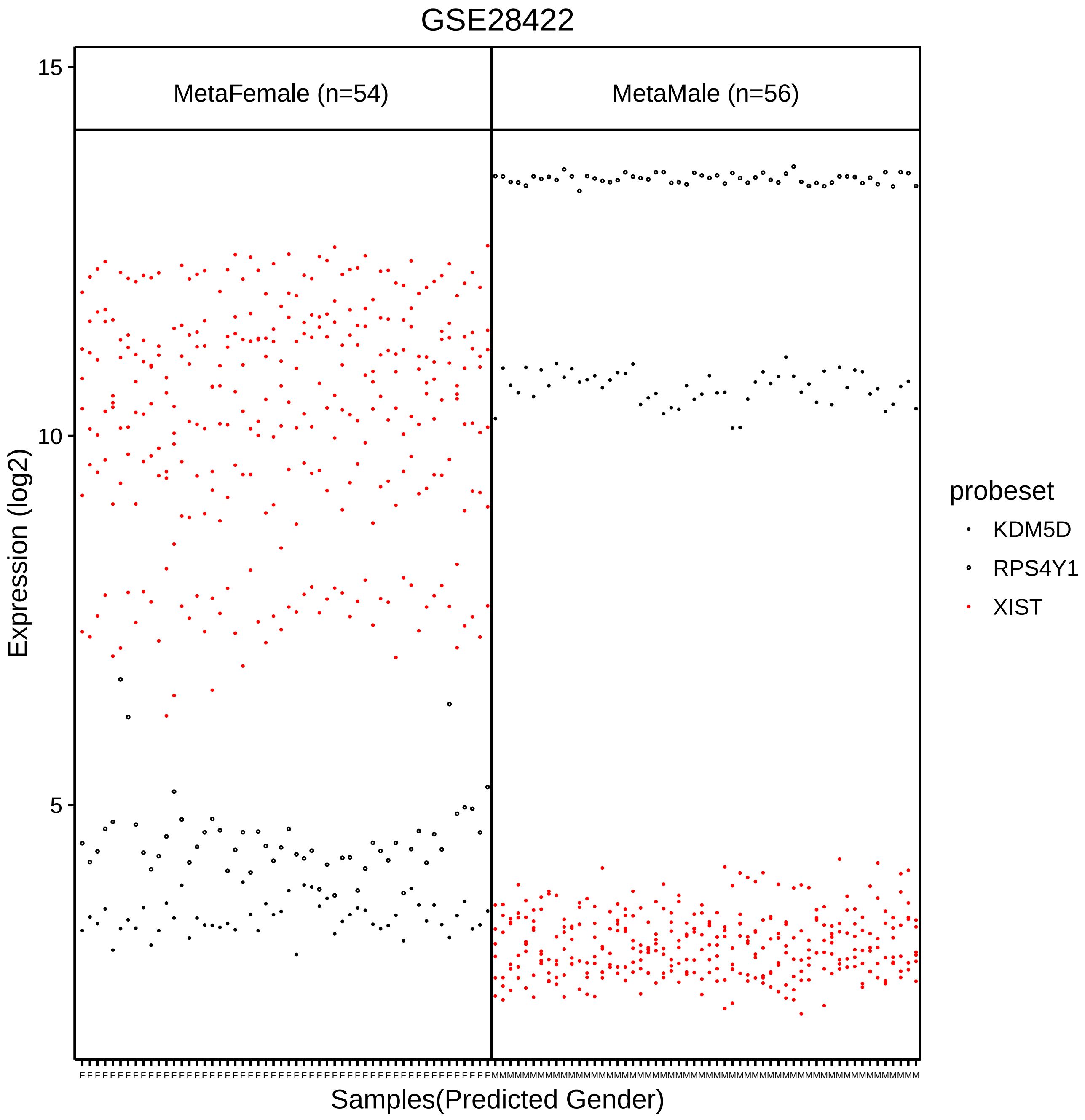

GSE28422
MetaFemale (n=54)
MetaMale (n=56)
Expression (log2)
15
10
5
Samples(Predicted Gender)
probeset
KDM5D
RPS4Y1
XIST

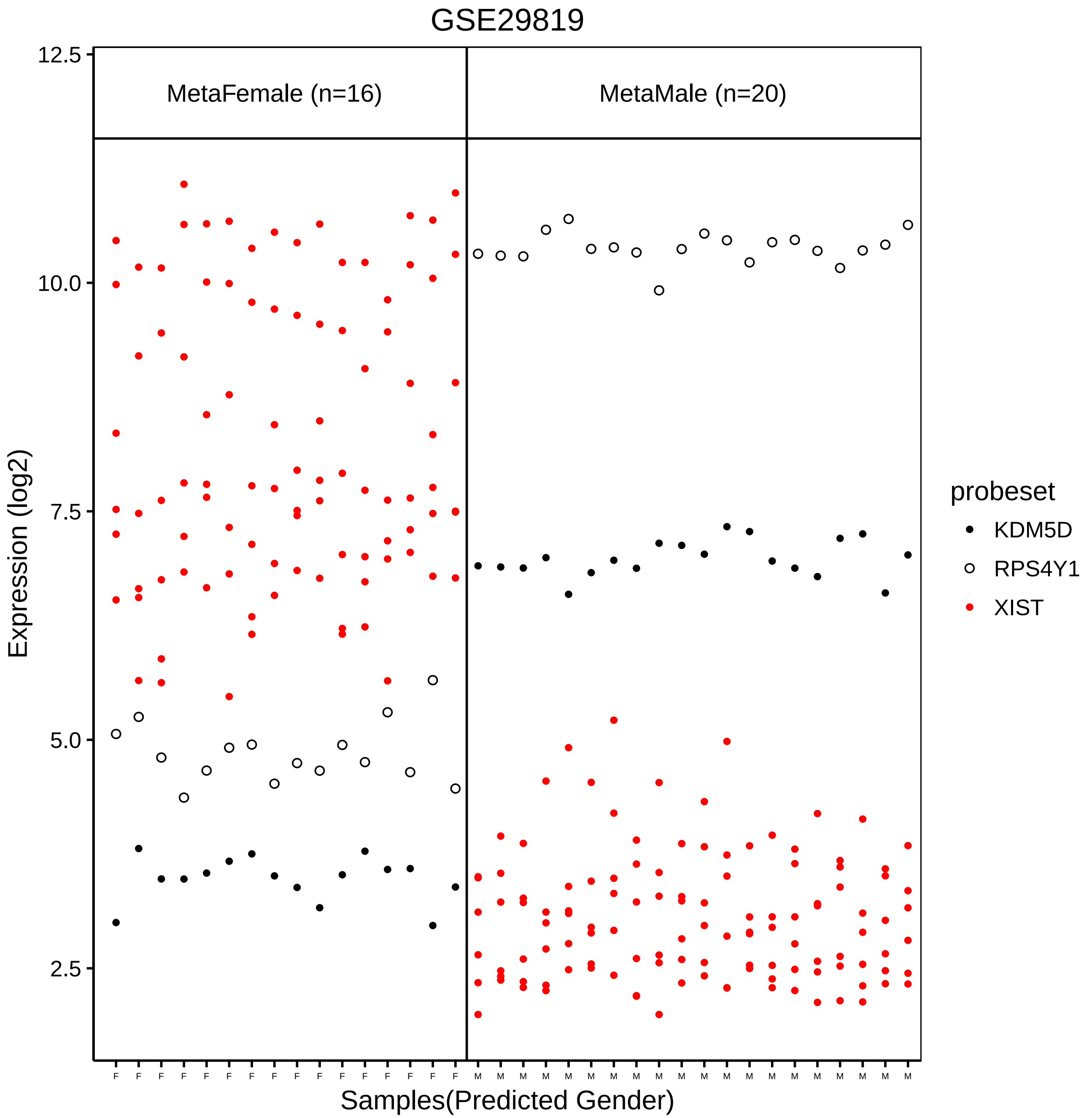
GSE29819
MetaFemale (n=16)
MetaMale (n=20)
Expression (log2)
12.5
10.0
7.5
5.0
2.5
F F F F F F F F F F F F F F F F
M M M M M M M M M M M M M M M M M M M M
Samples(Predicted Gender)
probeset
KDM5D
RPS4Y1
XIST

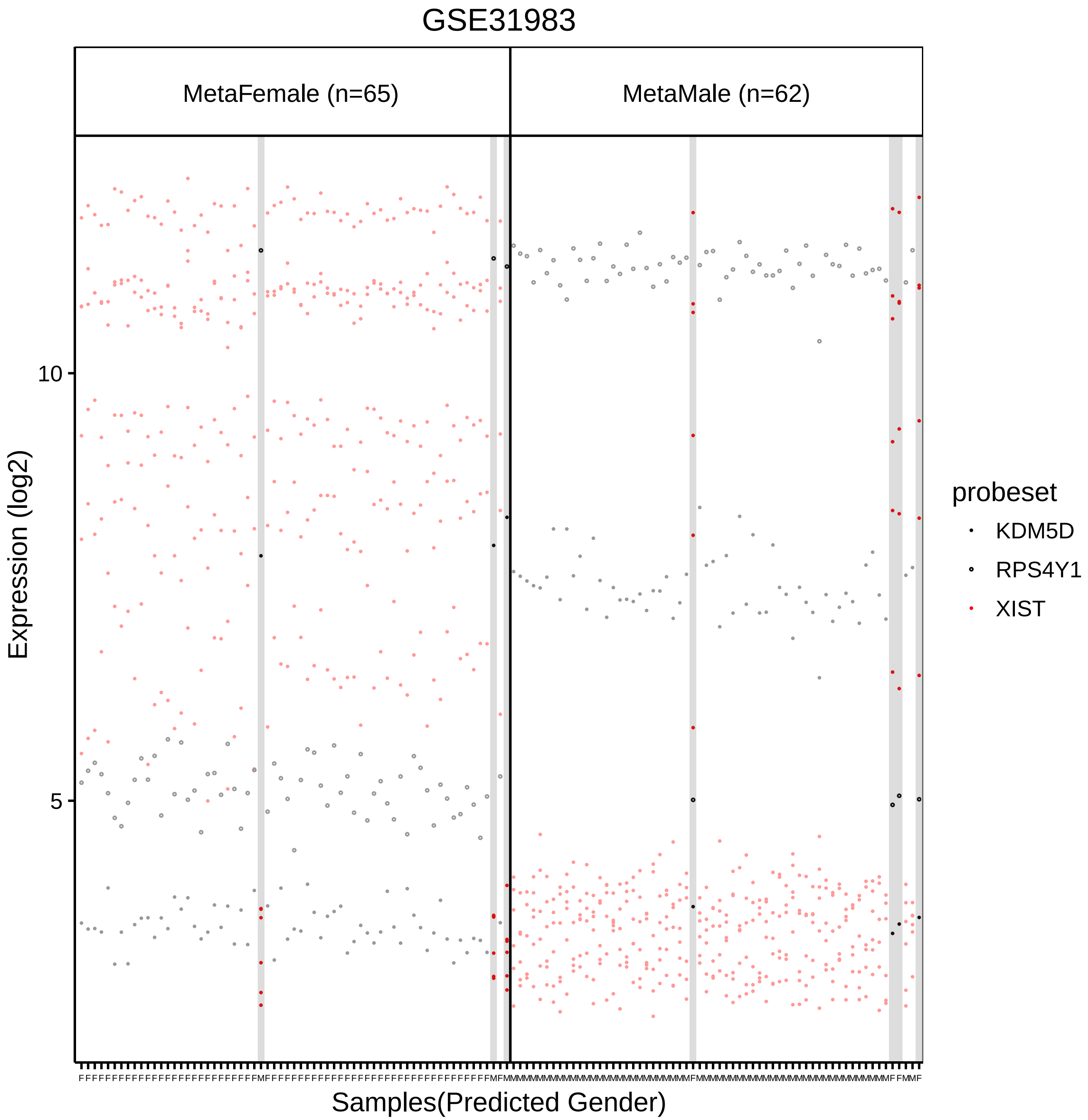
GSE31983
MetaFemale (n=65)
MetaMale (n=62)
Expression (log2)
10
5
Samples(Predicted Gender)
probeset
KDM5D
RPS4Y1
XIST

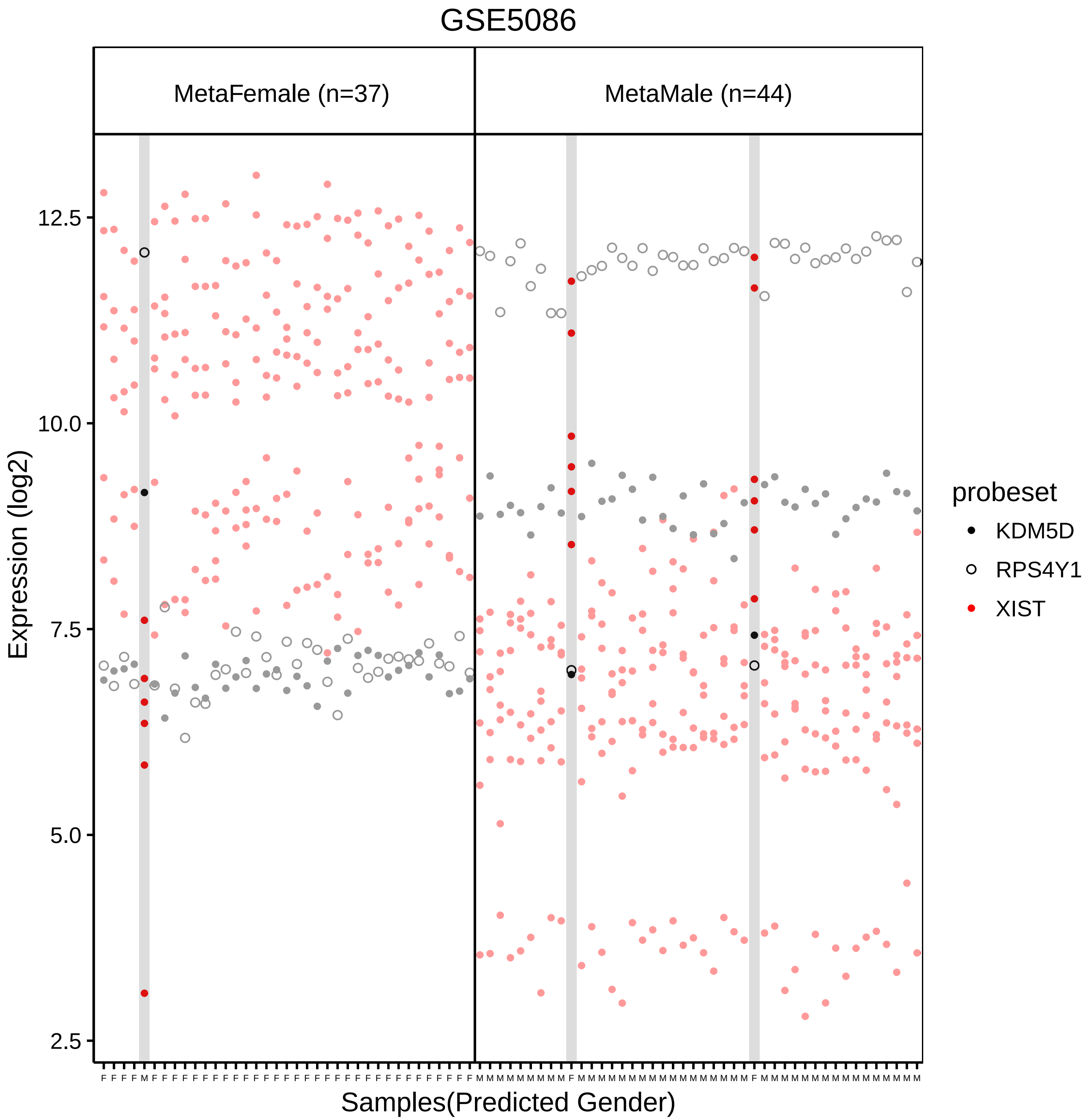

GSE5086
MetaFemale (n=37)
MetaMale (n=44)
Expression (log2)
12.5
10.0
7.5
5.0
2.5
Samples(Predicted Gender)
probeset
KDM5D
RPS4Y1
XIST

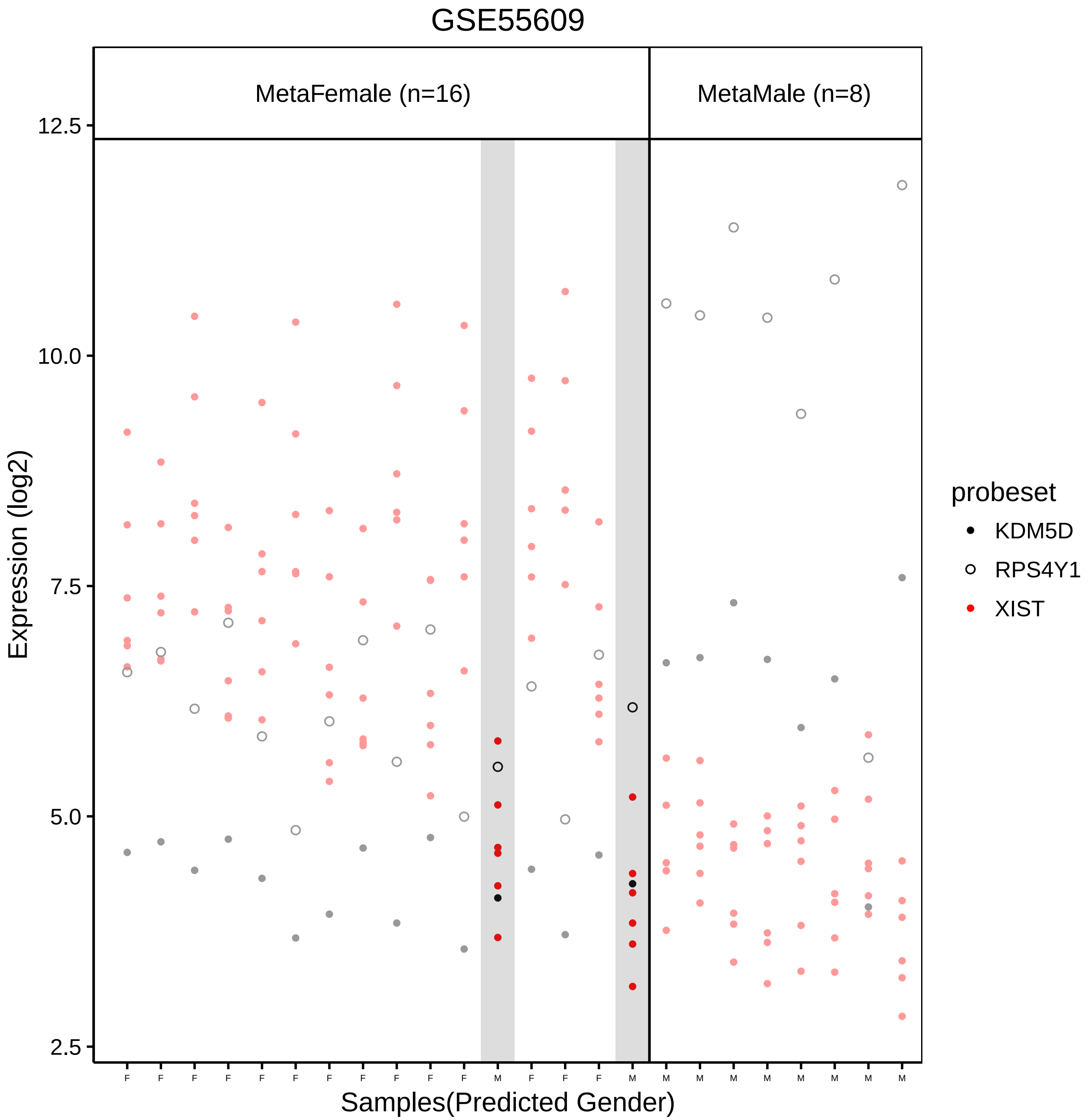
GSE55609
MetaFemale (n=16)
MetaMale (n=8)
Expression (log2)
12.5
10.0
7.5
5.0
2.5
F F F F F F F F F F F M F F F M M M M M M M M M
Samples(Predicted Gender)
probeset
KDM5D
RPS4Y1
XIST

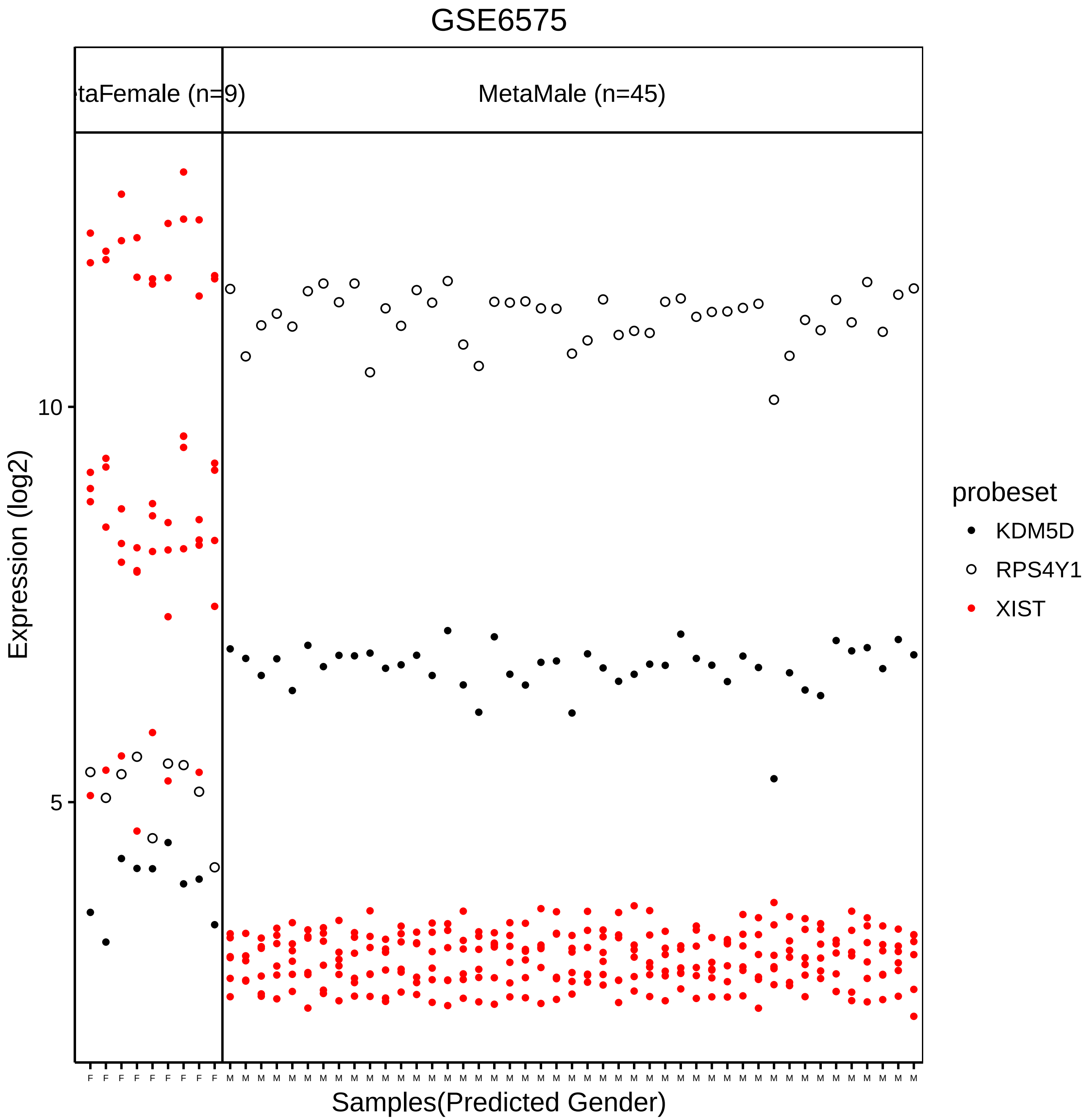
GSE6575
etaFemale (n=9)
MetaMale (n=45)
Expression (log2)
10
5
F F F F F F F F F
M M M M M M M M M M M M M M M M M M M M M M M M M M M M M M M M M M M M M M M M M M M M M
Samples(Predicted Gender)
probeset
KDM5D
RPS4Y1
XIST

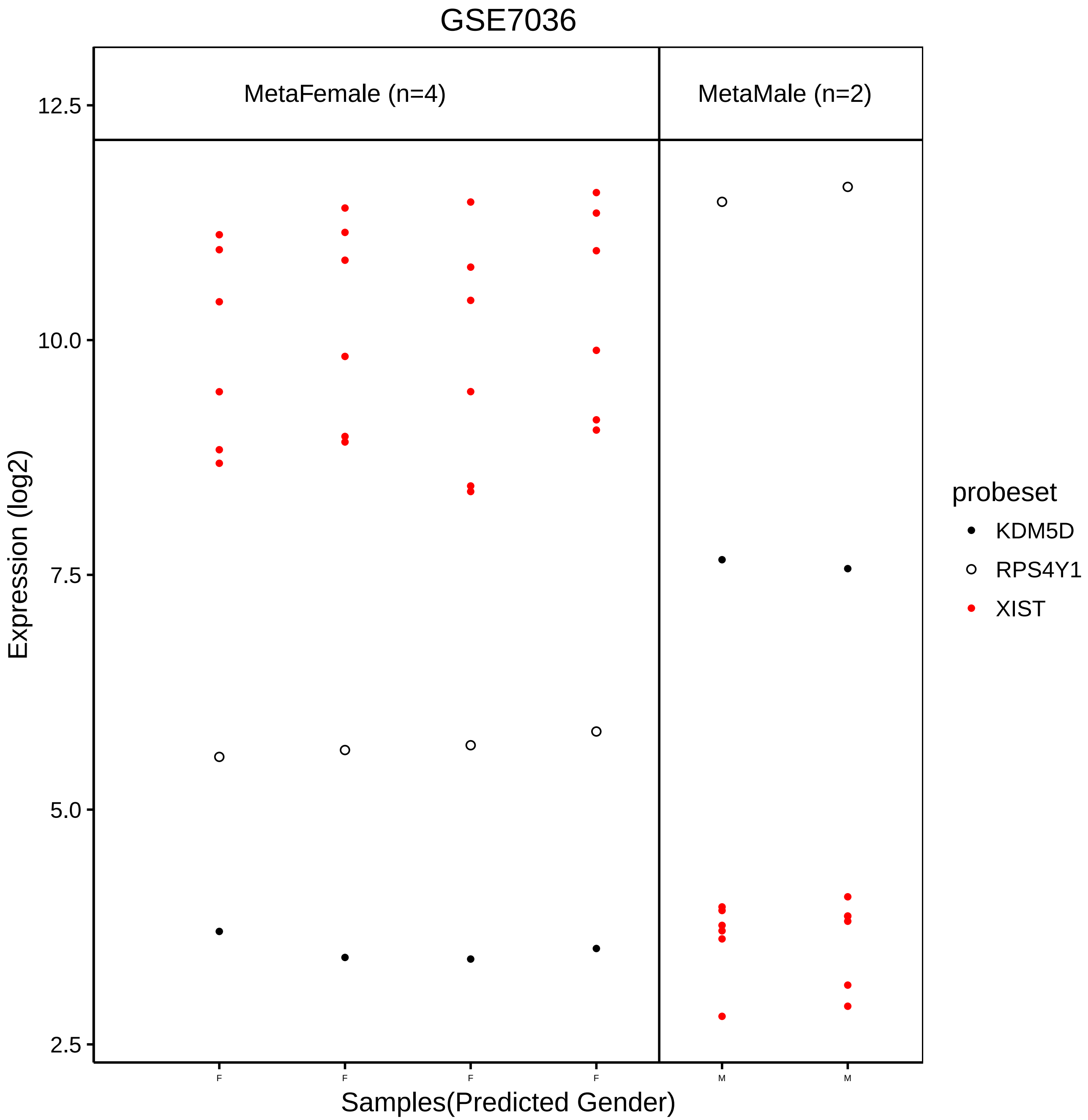
GSE7036
MetaFemale (n=4)
MetaMale (n=2)
12.5
10.0
7.5
5.0
2.5
Expression (log2)
F
F
F
F
M
M
Samples(Predicted Gender)
probeset
KDM5D
RPS4Y1
XIST

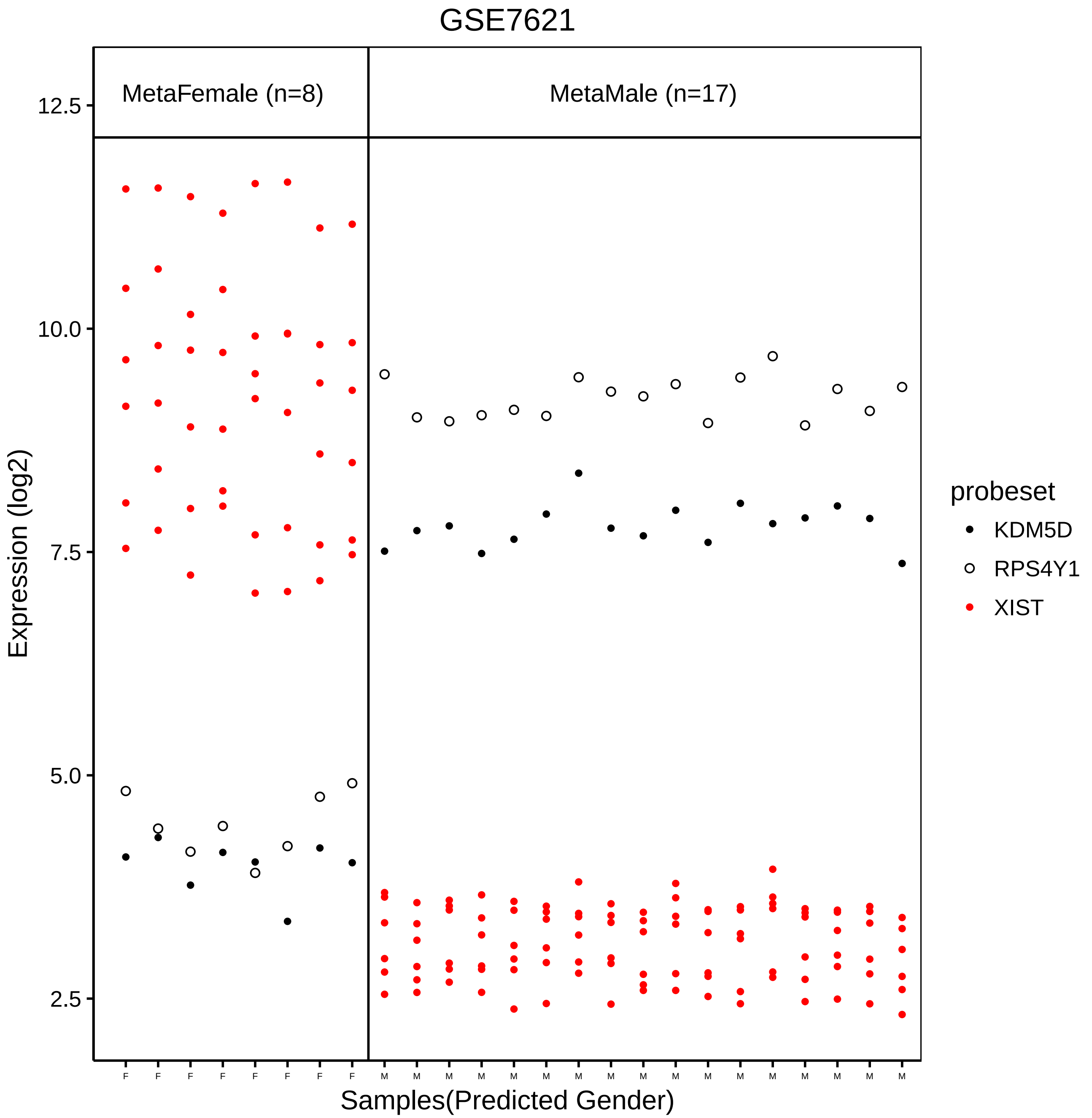

GSE7621
MetaFemale (n=8)
MetaMale (n=17)
Expression (log2)
12.5
10.0
7.5
5.0
2.5
F F F F F F F F
M M M M M M M M M M M M M M M M M
Samples(Predicted Gender)
probeset
KDM5D
RPS4Y1
XIST

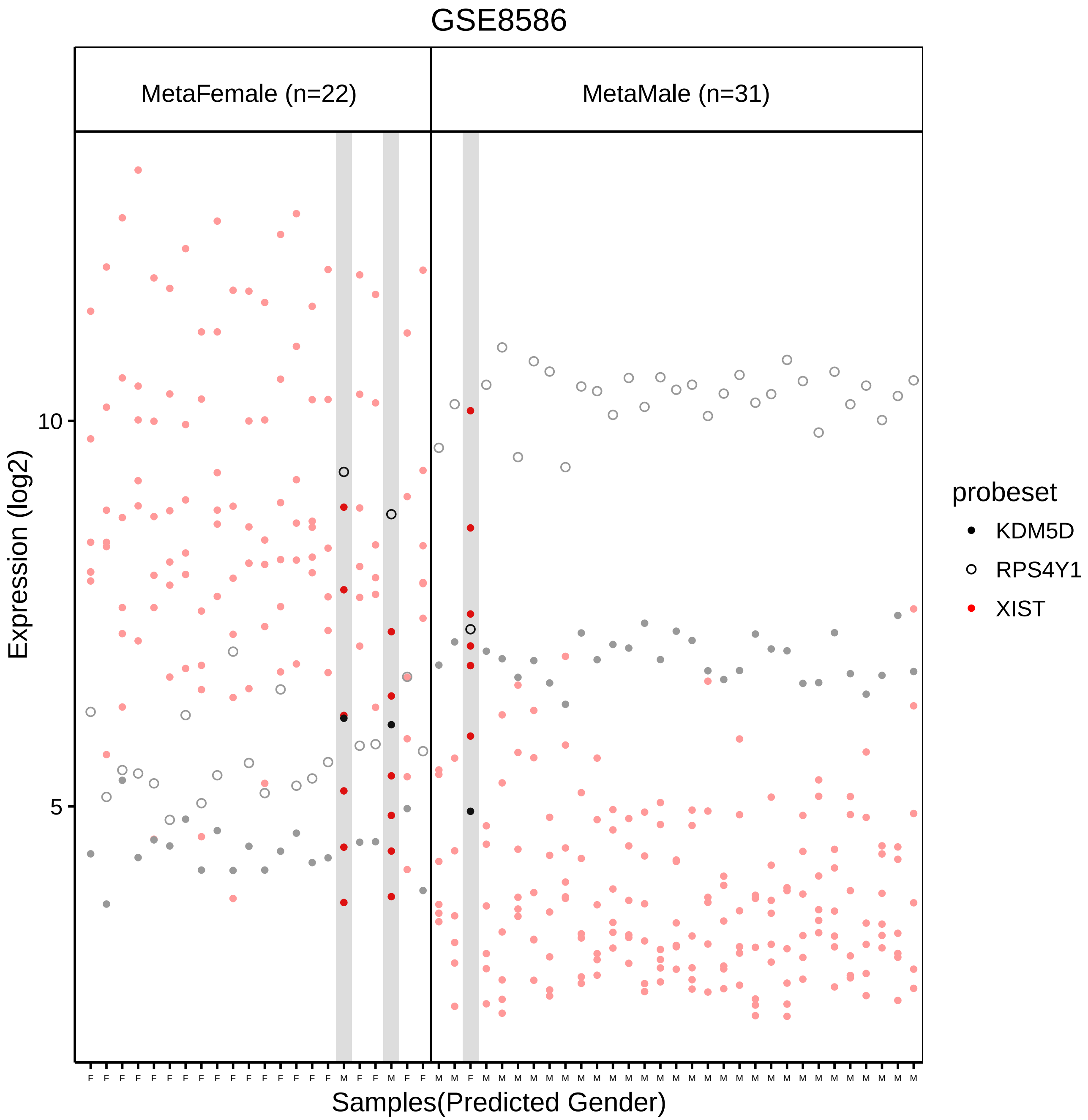

GSE8586
MetaFemale (n=22)
MetaMale (n=31)
Expression (log2)
10
5
Samples(Predicted Gender)
F F F F F F F F F F F F F F F F M F F M F F
M M F M M M M M M M M M M M M M M M M M M M M M M M M M M M M
probeset
KDM5D
RPS4Y1
XIST

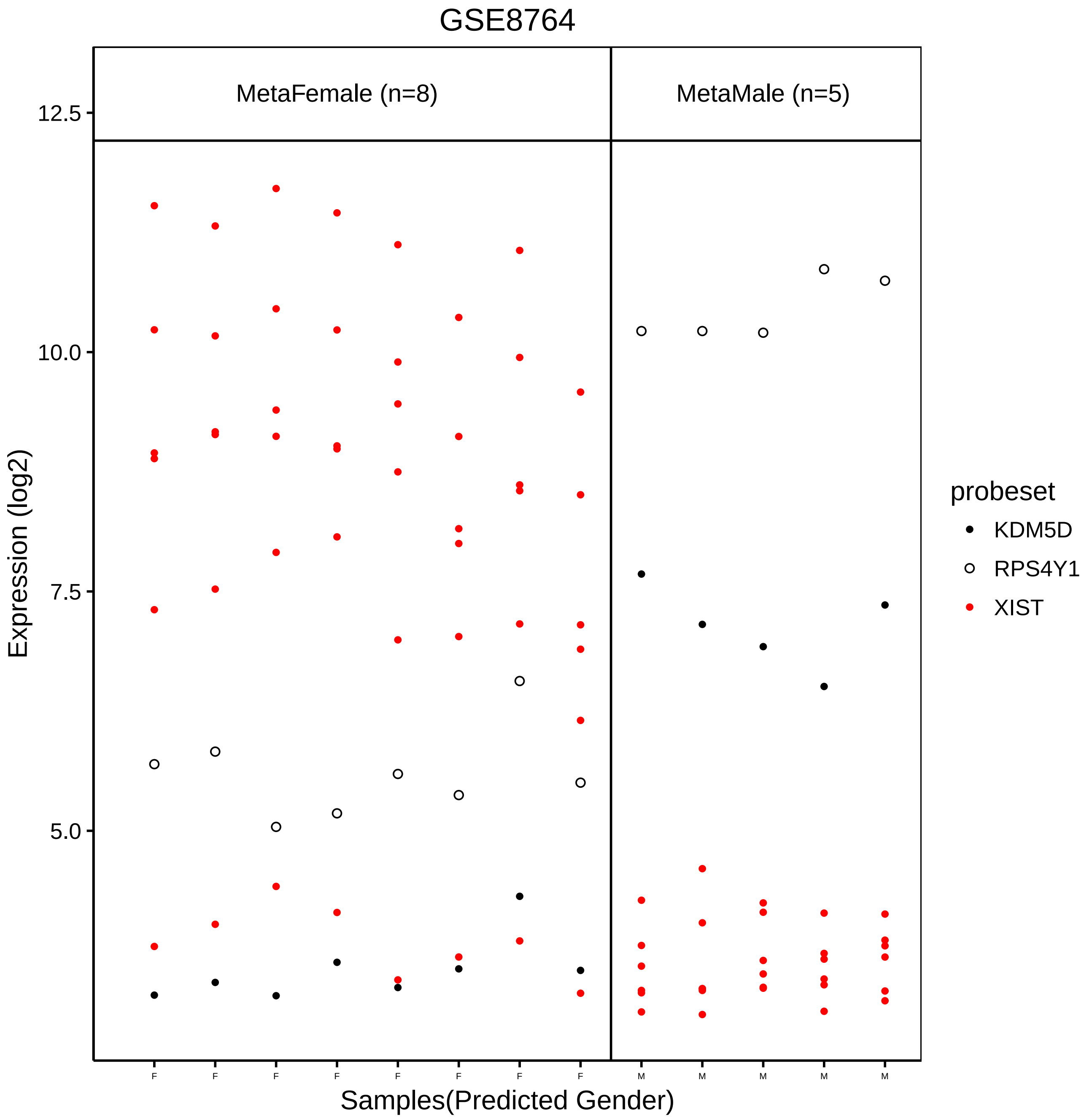
GSE8764
MetaFemale (n=8)
MetaMale (n=5)
Expression (log2)
12.5
10.0
7.5
5.0
F
M
Samples(Predicted Gender)
probeset
KDM5D
RPS4Y1
XIST

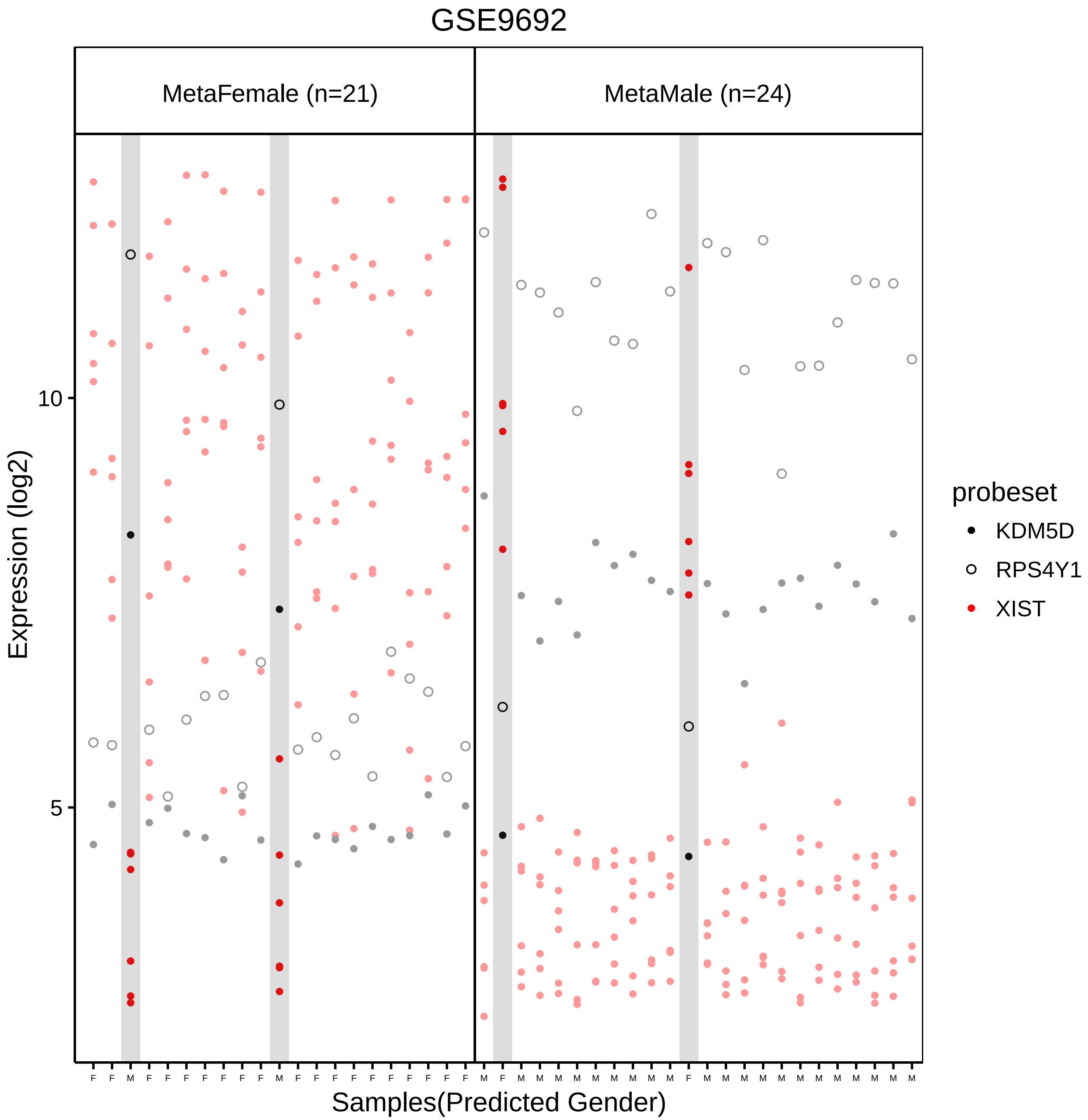
GSE9692
MetaFemale (n=21)
MetaMale (n=24)
Expression (log2)
10
5
Samples(Predicted Gender)
F F M F F F F F F F M F F F F F F F F F F M F M M M M M M M M M F M M M M M M M M M M M M
probeset
KDM5D
RPS4Y1
XIST

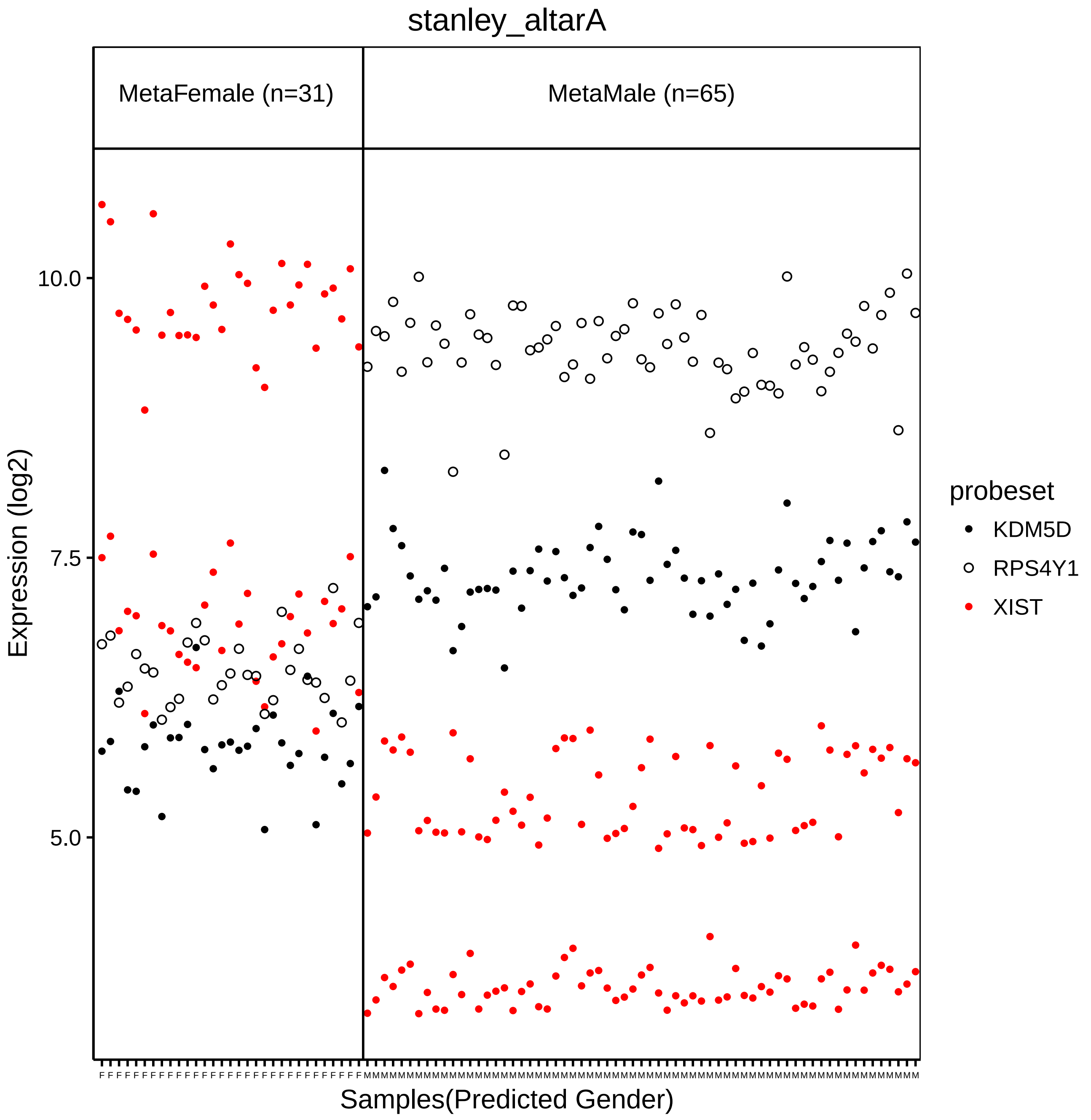
stanley_altarA
MetaFemale (n=31)
MetaMale (n=65)
Expression (log2)
10.0
7.5
5.0
Samples(Predicted Gender)
probeset
KDM5D
RPS4Y1
XIST

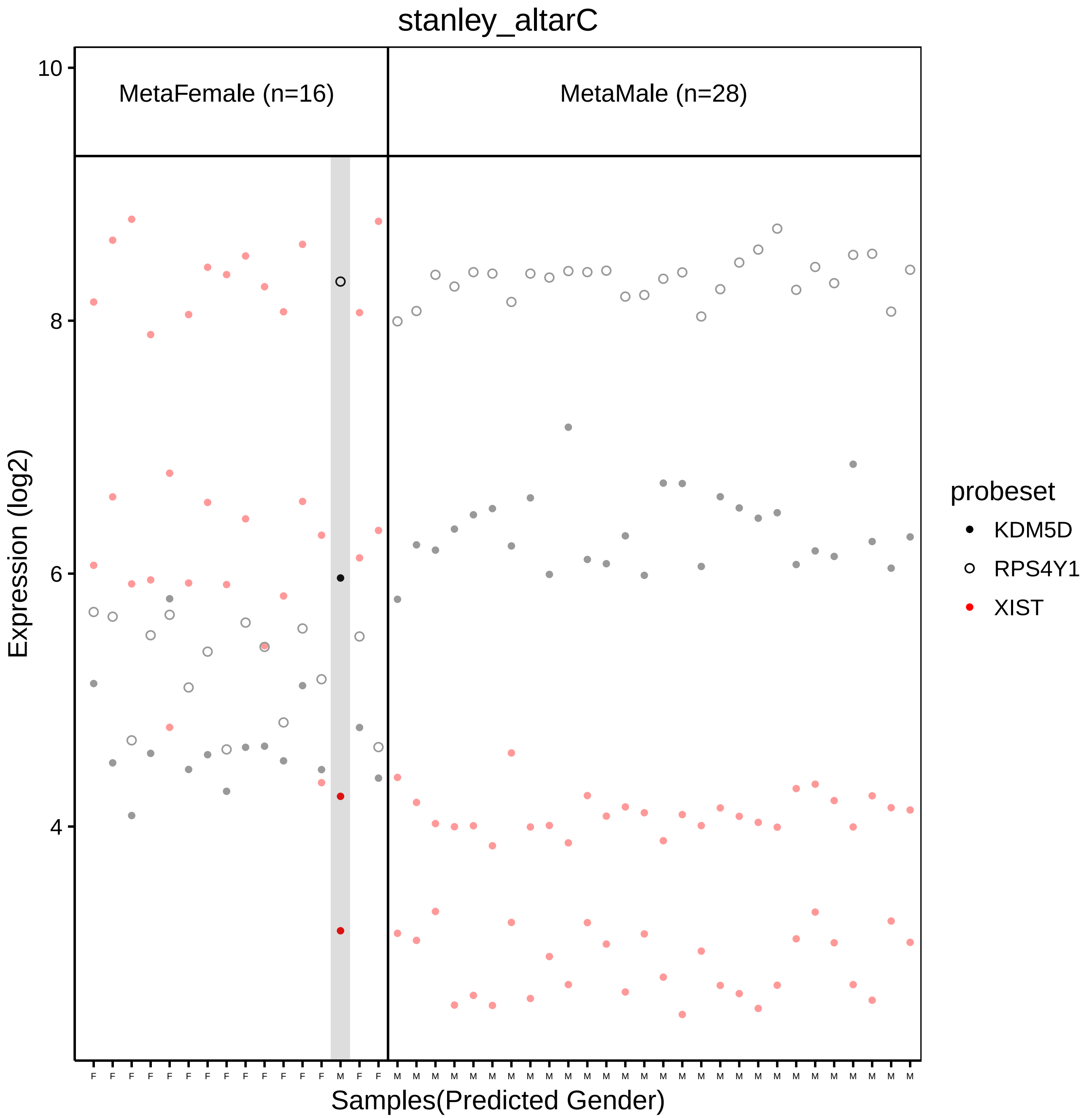
stanley_altarC
MetaFemale (n=16)
MetaMale (n=28)
Expression (log2)
10
8
6
4
Samples(Predicted Gender)
probeset
KDM5D
RPS4Y1
XIST
F F F F F F F F F F F F F M F F M M M M M M M M M M M M M M M M M M M M M M M M M M M M

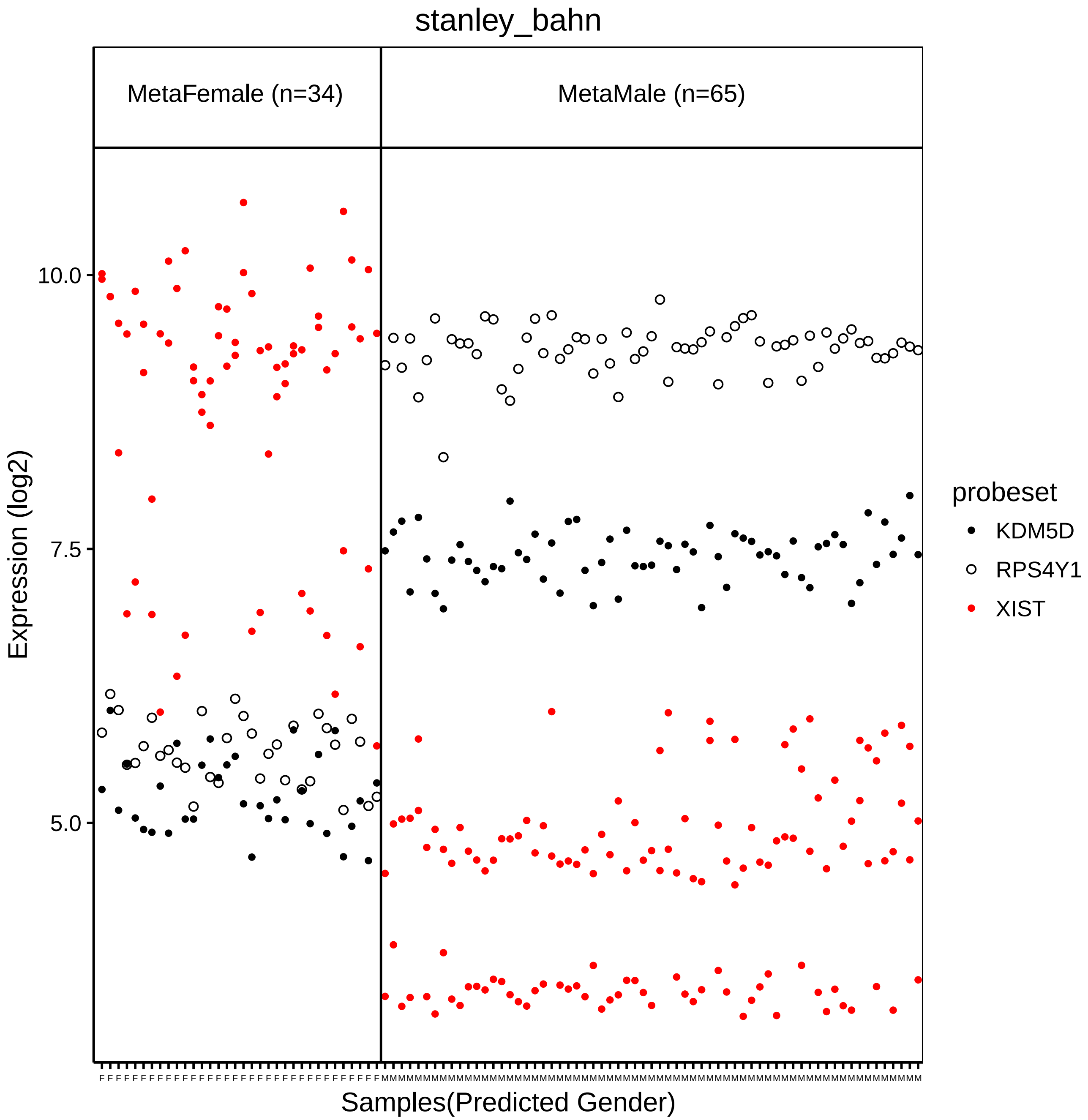

stanley_bahn
MetaFemale (n=34)
MetaMale (n=65)
Expression (log2)
10.0
7.5
5.0
Samples(Predicted Gender)
probeset
KDM5D
RPS4Y1
XIST

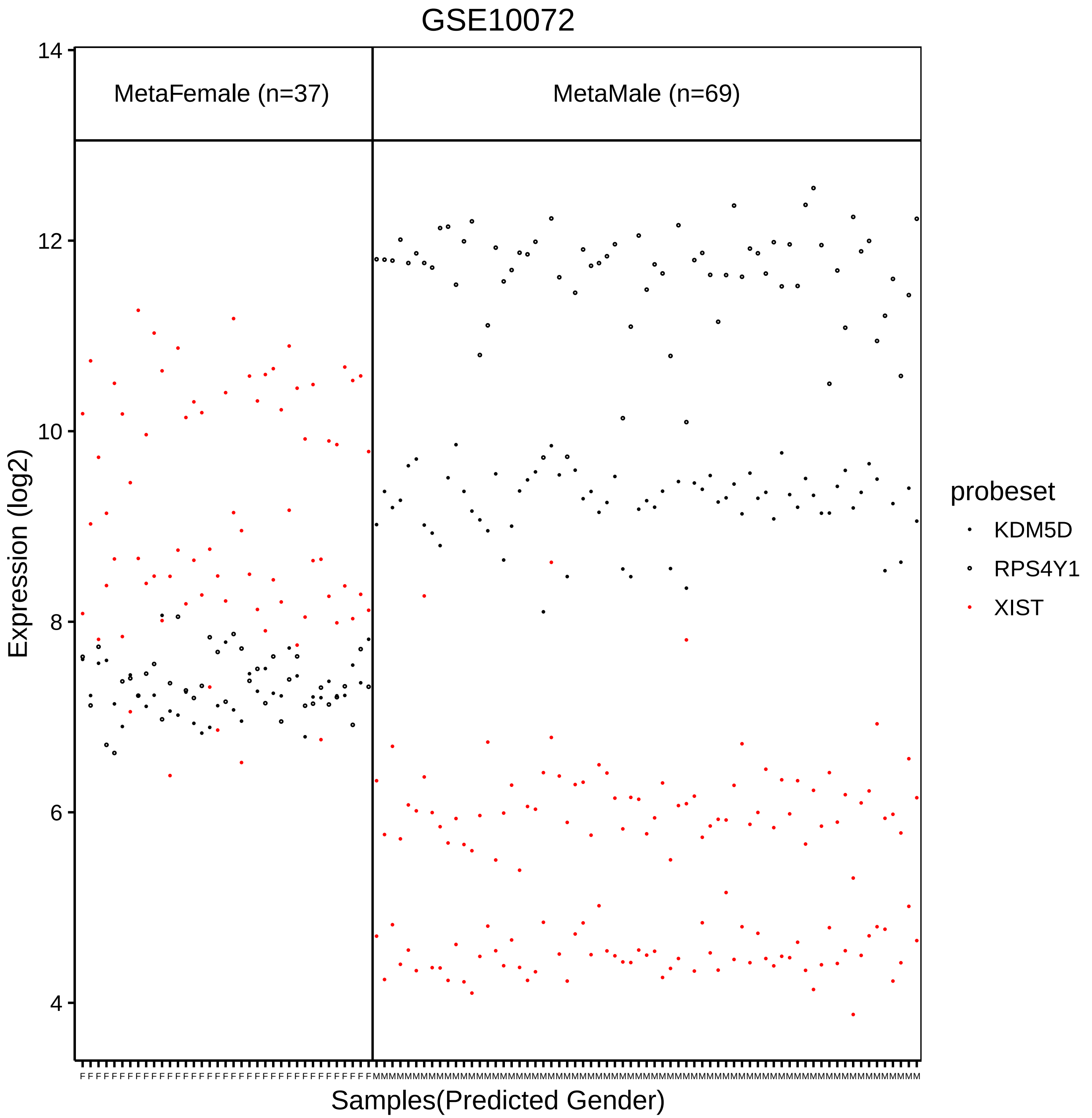

GSE10072
MetaFemale (n=37)
MetaMale (n=69)
Expression (log2)
14
12
10
8
6
4
Samples(Predicted Gender)
probeset
KDM5D
RPS4Y1
XIST

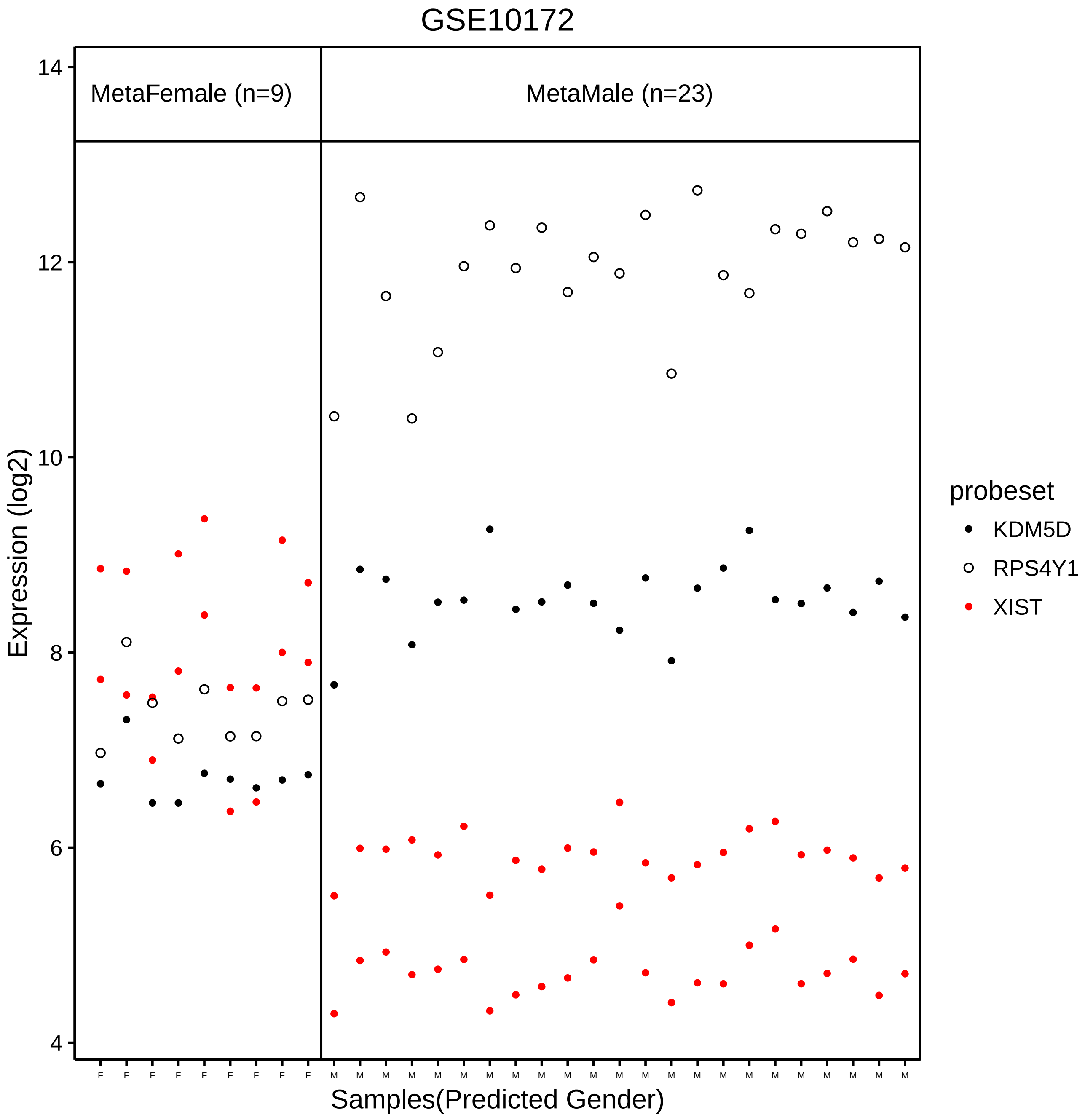

GSE10172
MetaFemale (n=9)
MetaMale (n=23)
Expression (log2)
14
12
10
8
6
4
Samples(Predicted Gender)
probeset
KDM5D
RPS4Y1
XIST

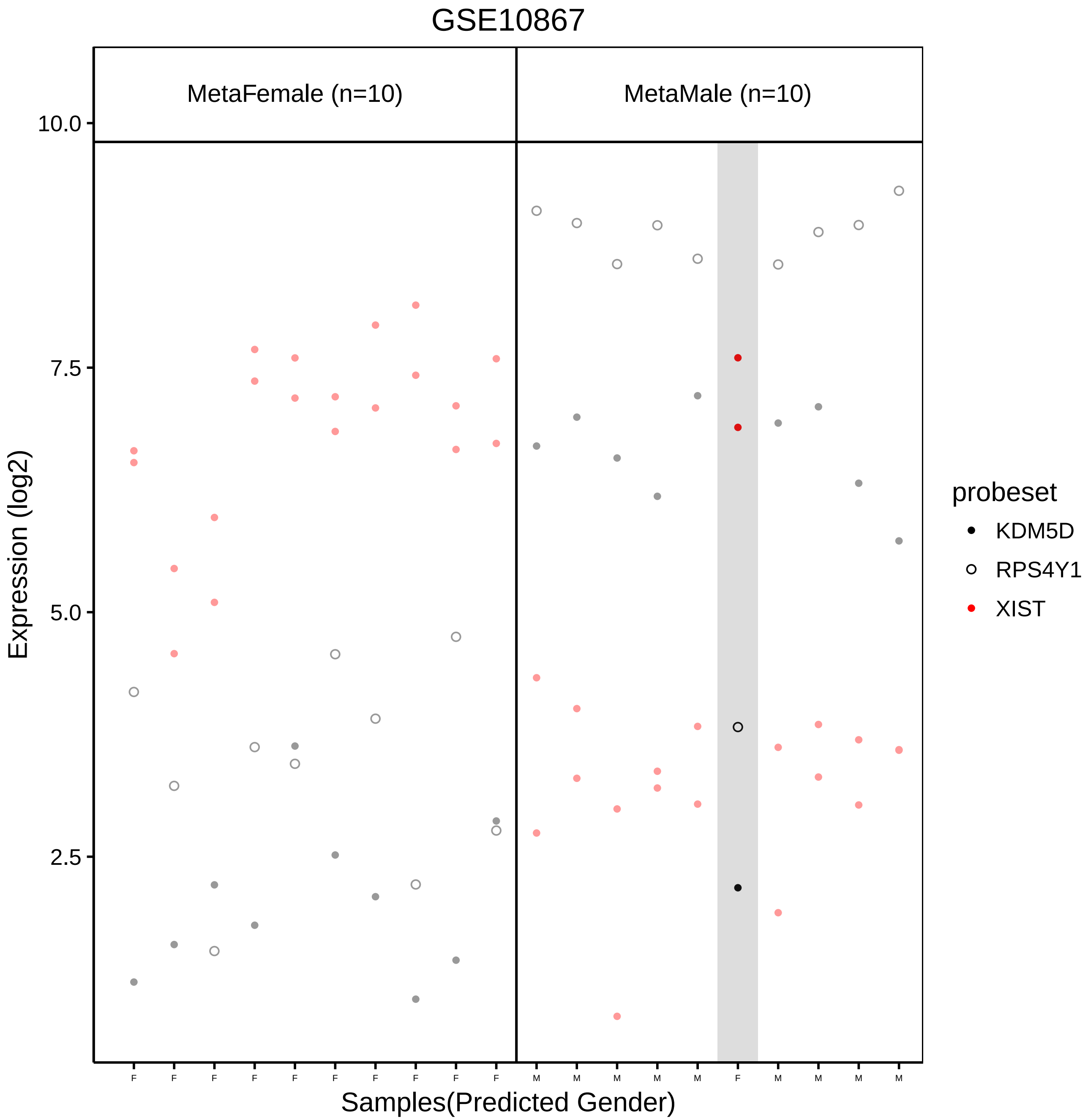
GSE10867
MetaFemale (n=10)
MetaMale (n=10)
Expression (log2)
10.0
7.5
5.0
2.5
F F F F F F F F F F M M M M M F M M M M
Samples(Predicted Gender)
probeset
KDM5D
RPS4Y1
XIST

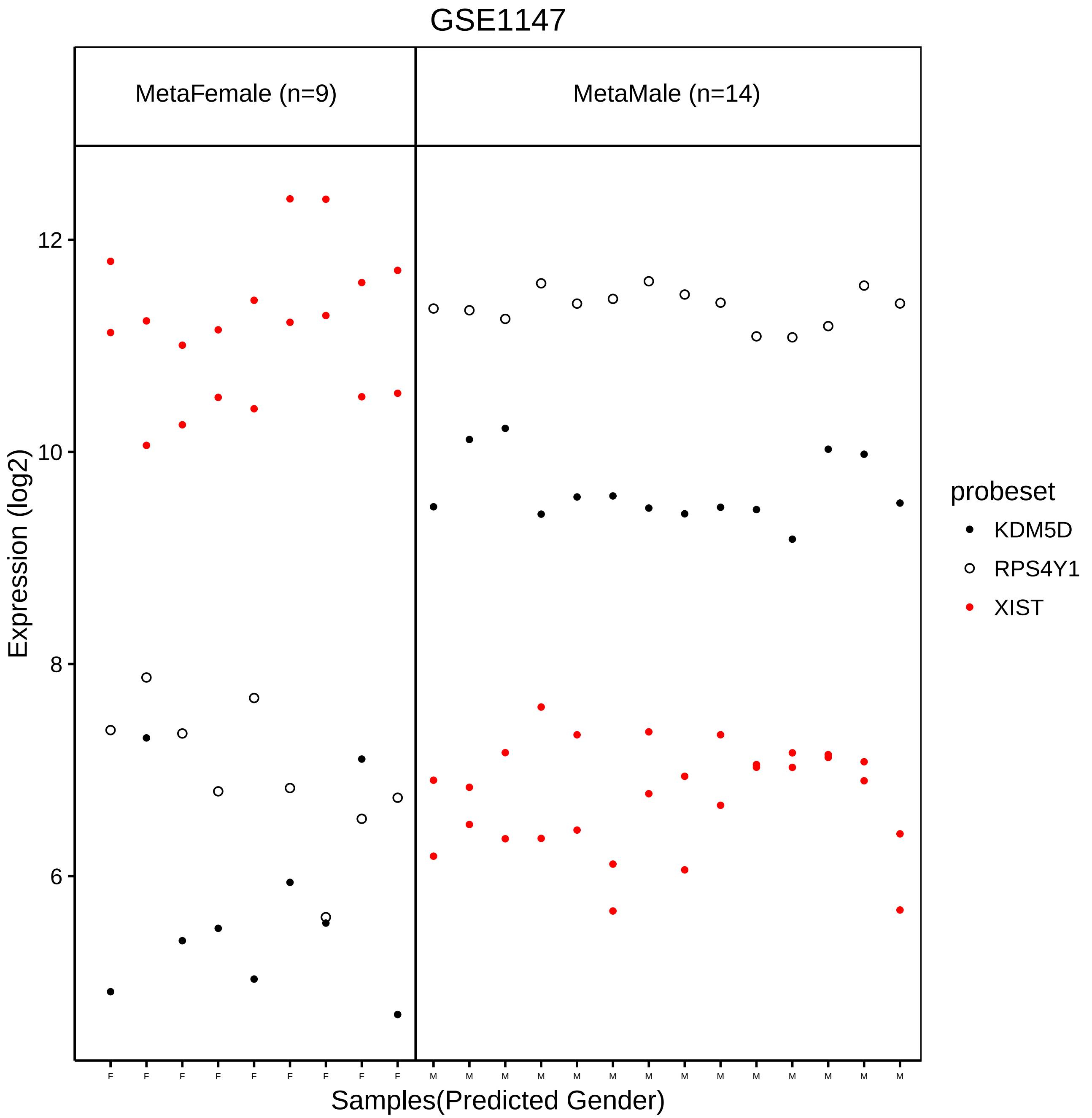
GSE1147
MetaFemale (n=9)
MetaMale (n=14)
Expression (log2)
12
10
8
6
F F F F F F F F F M M M M M M M M M M M M M M
Samples(Predicted Gender)
probeset
KDM5D
RPS4Y1
XIST

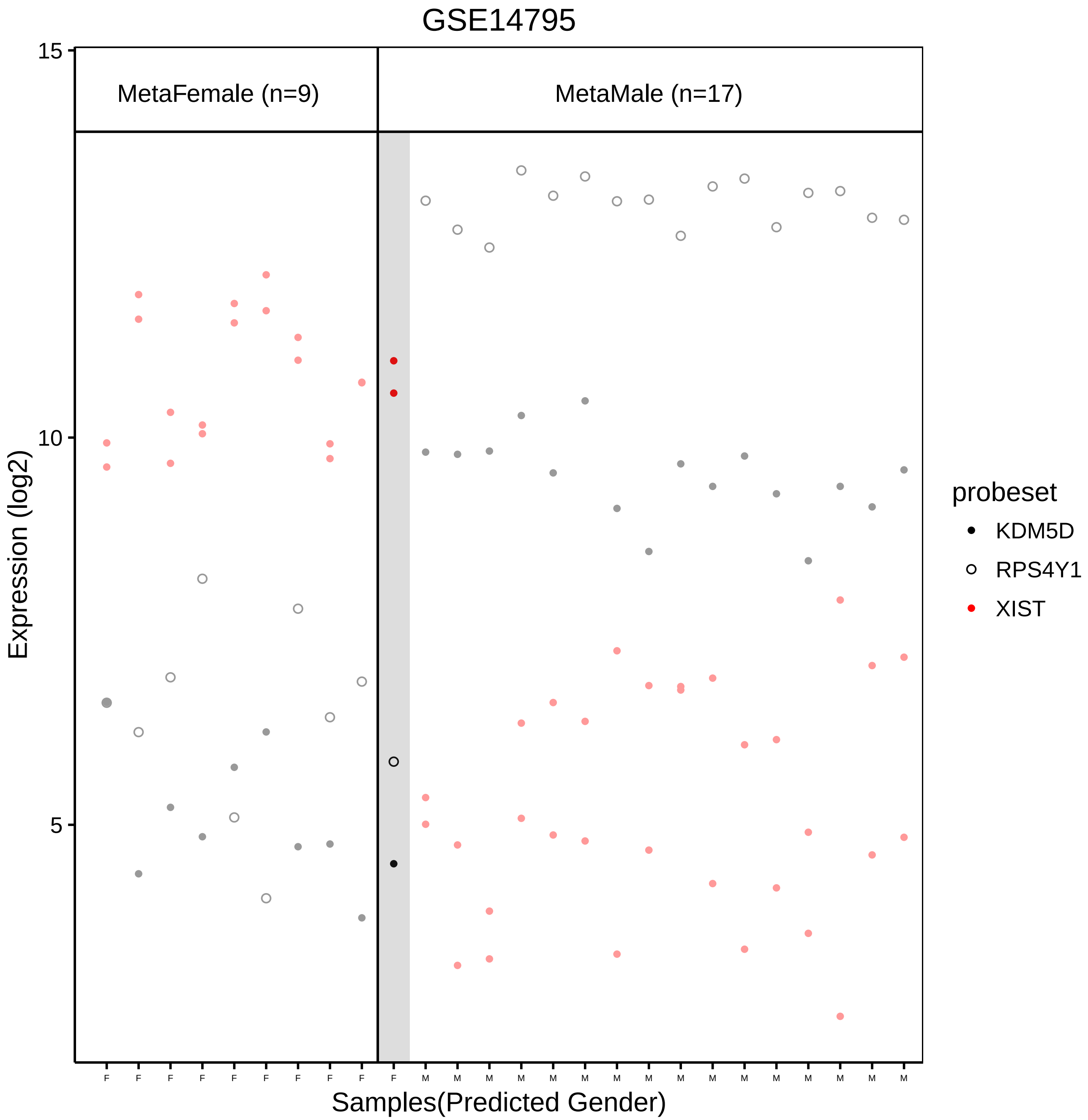

GSE14795
MetaFemale (n=9)
MetaMale (n=17)
Expression (log2)
15
10
5
Samples(Predicted Gender)
F F F F F F F F F F M M M M M M M M M M M M M M M M
probeset
KDM5D
RPS4Y1
XIST

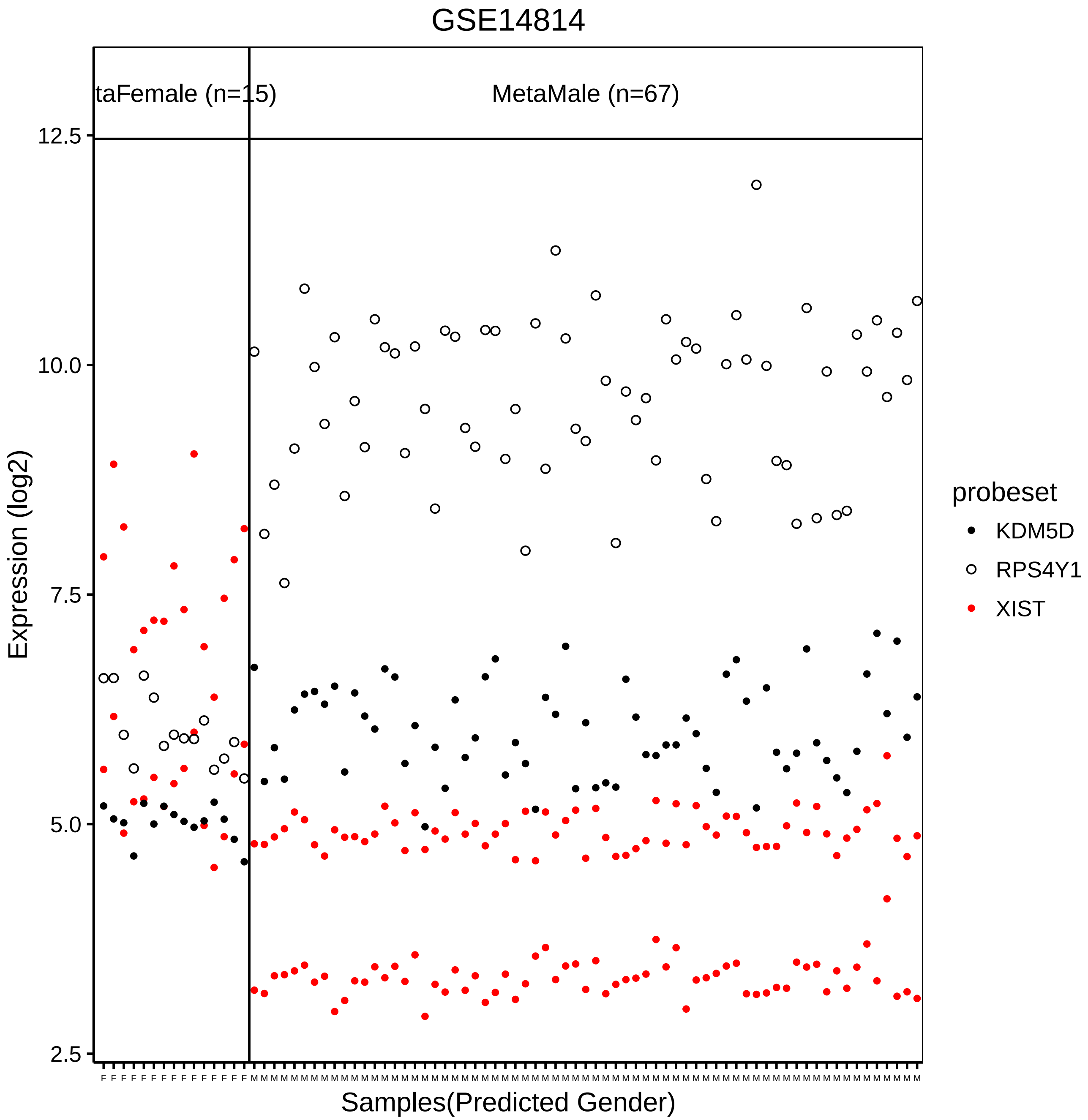
GSE14814
taFemale (n=15)
MetaMale (n=67)
12.5
10.0
7.5
5.0
2.5
Expression (log2)
Samples(Predicted Gender)
probeset
KDM5D
RPS4Y1
XIST

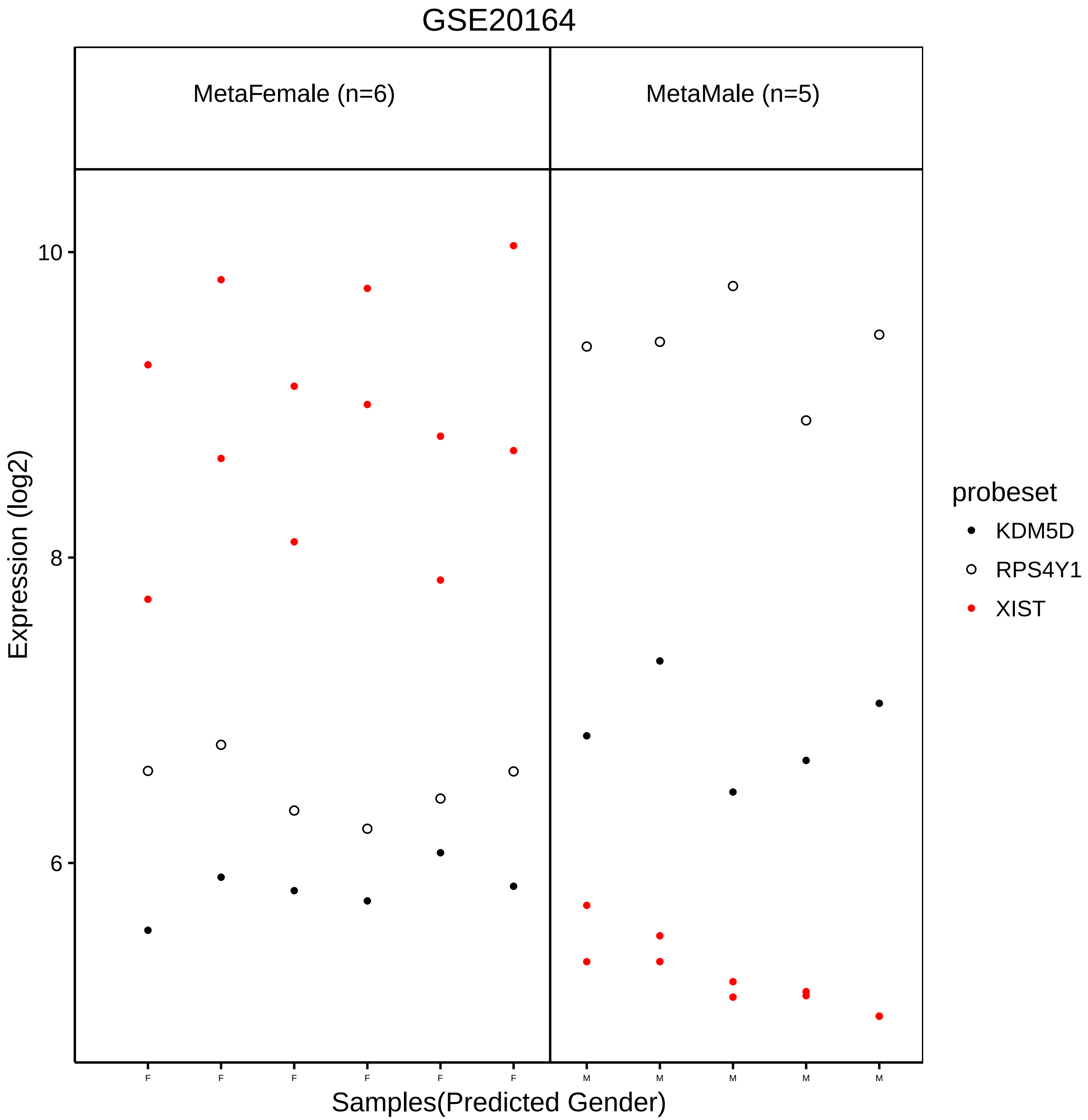
GSE20164
MetaFemale (n=6)
MetaMale (n=5)
Expression (log2)
10
8
6
F
F
F
F
F
F
M
M
M
M
M
Samples(Predicted Gender)
probeset
KDM5D
RPS4Y1
XIST

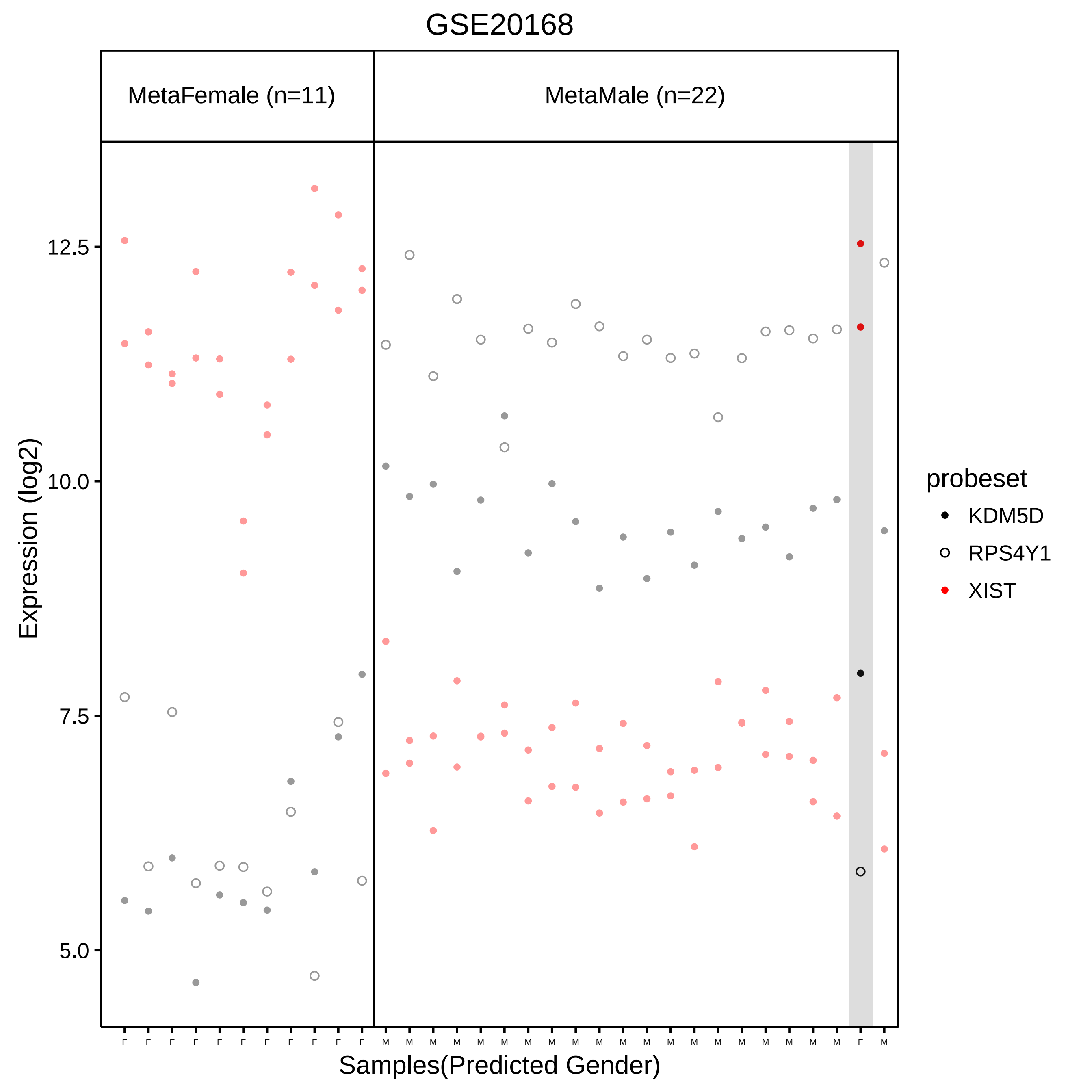
GSE20168
MetaFemale (n=11)
MetaMale (n=22)
Expression (log2)
12.5
10.0
7.5
5.0
Samples(Predicted Gender)
F F F F F F F F F F F M M M M M M M M M M M M M M M M M M M M F M
probeset
KDM5D
RPS4Y1
XIST

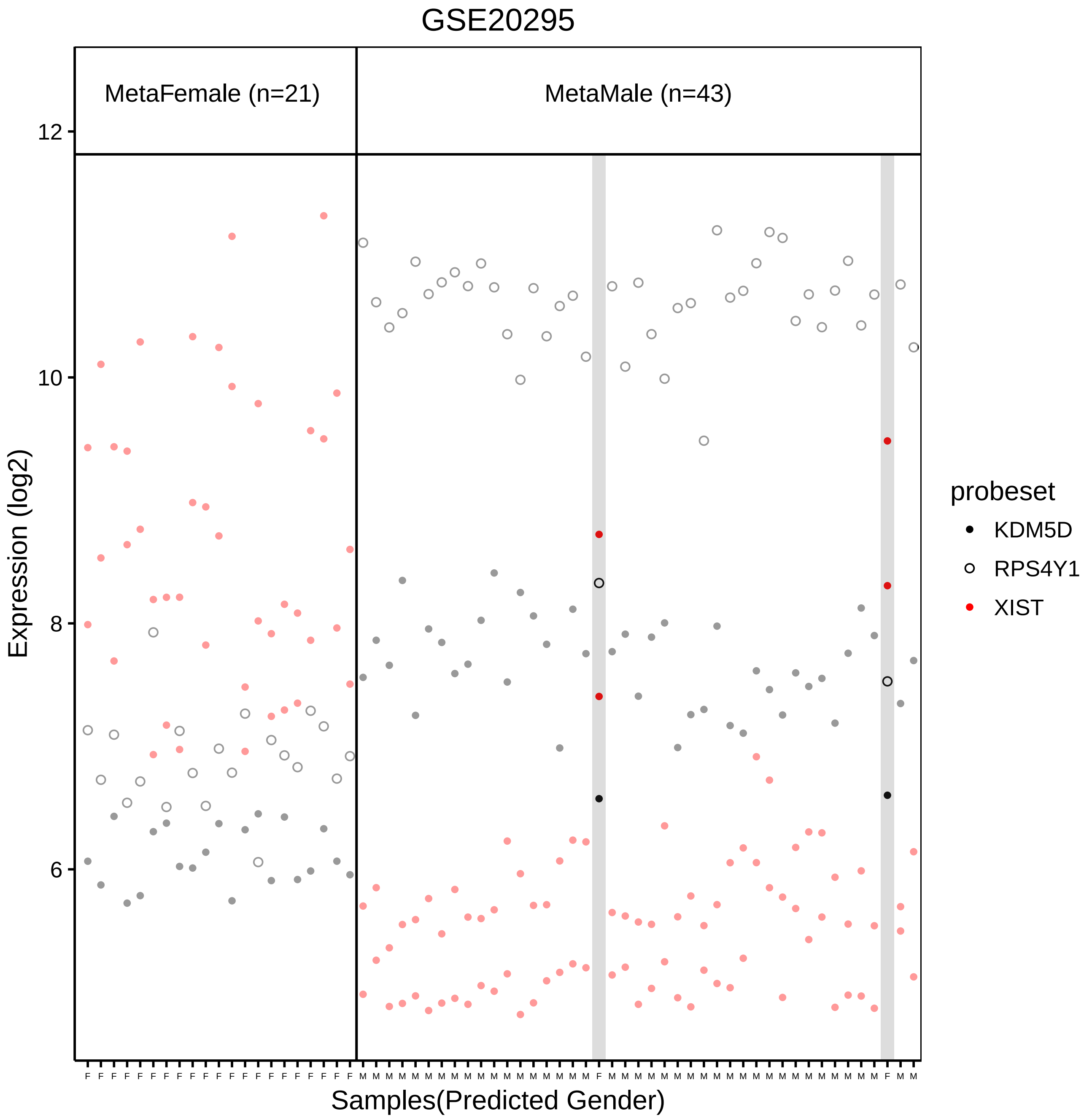

GSE20295
MetaFemale (n=21)
MetaMale (n=43)
Expression (log2)
12
10
8
6
Samples(Predicted Gender)
probeset
KDM5D
RPS4Y1
XIST

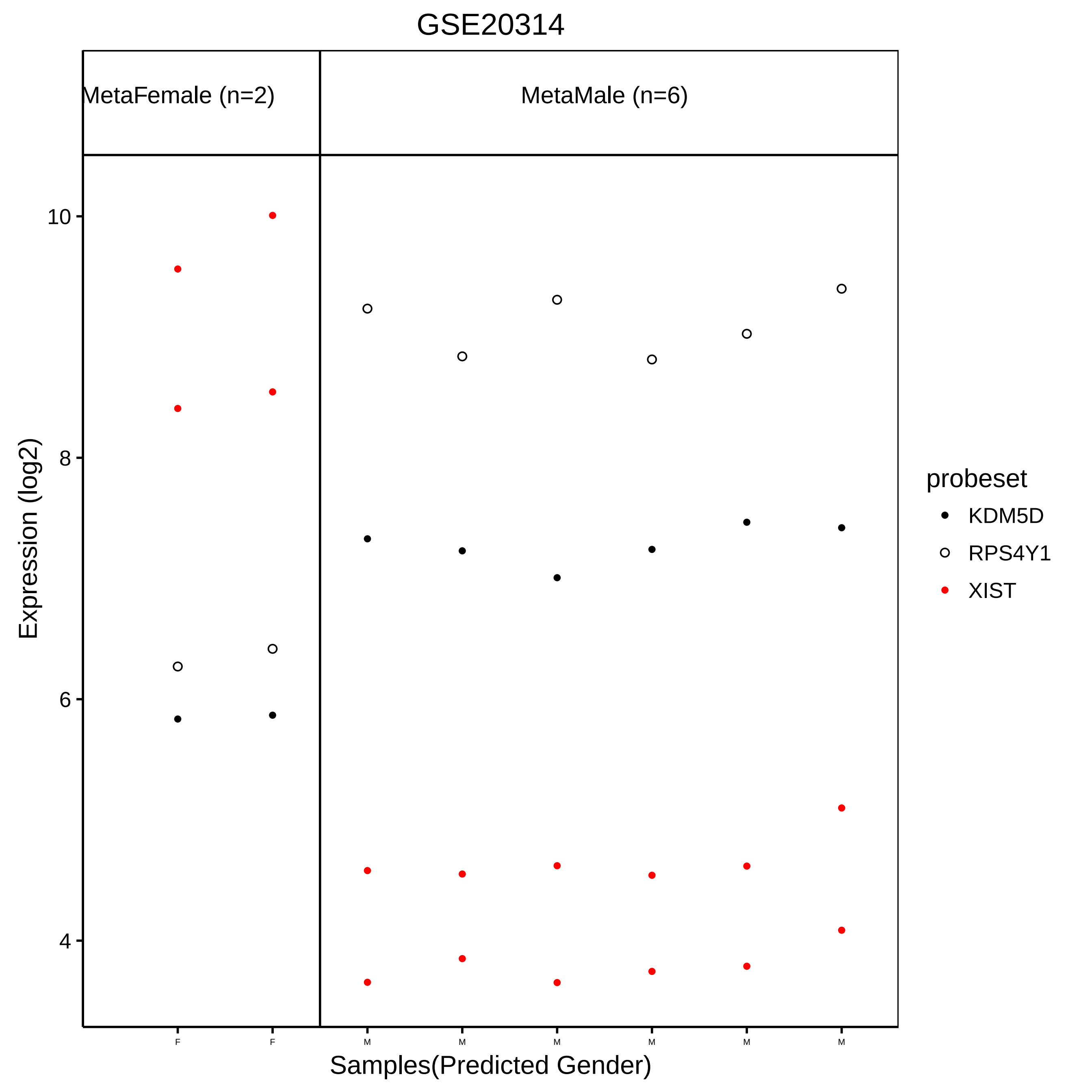
GSE20314
MetaFemale (n=2)
MetaMale (n=6)
Expression (log2)
10
8
6
4
F
F
M
M
M
M
M
M
Samples(Predicted Gender)
probeset
KDM5D
RPS4Y1
XIST

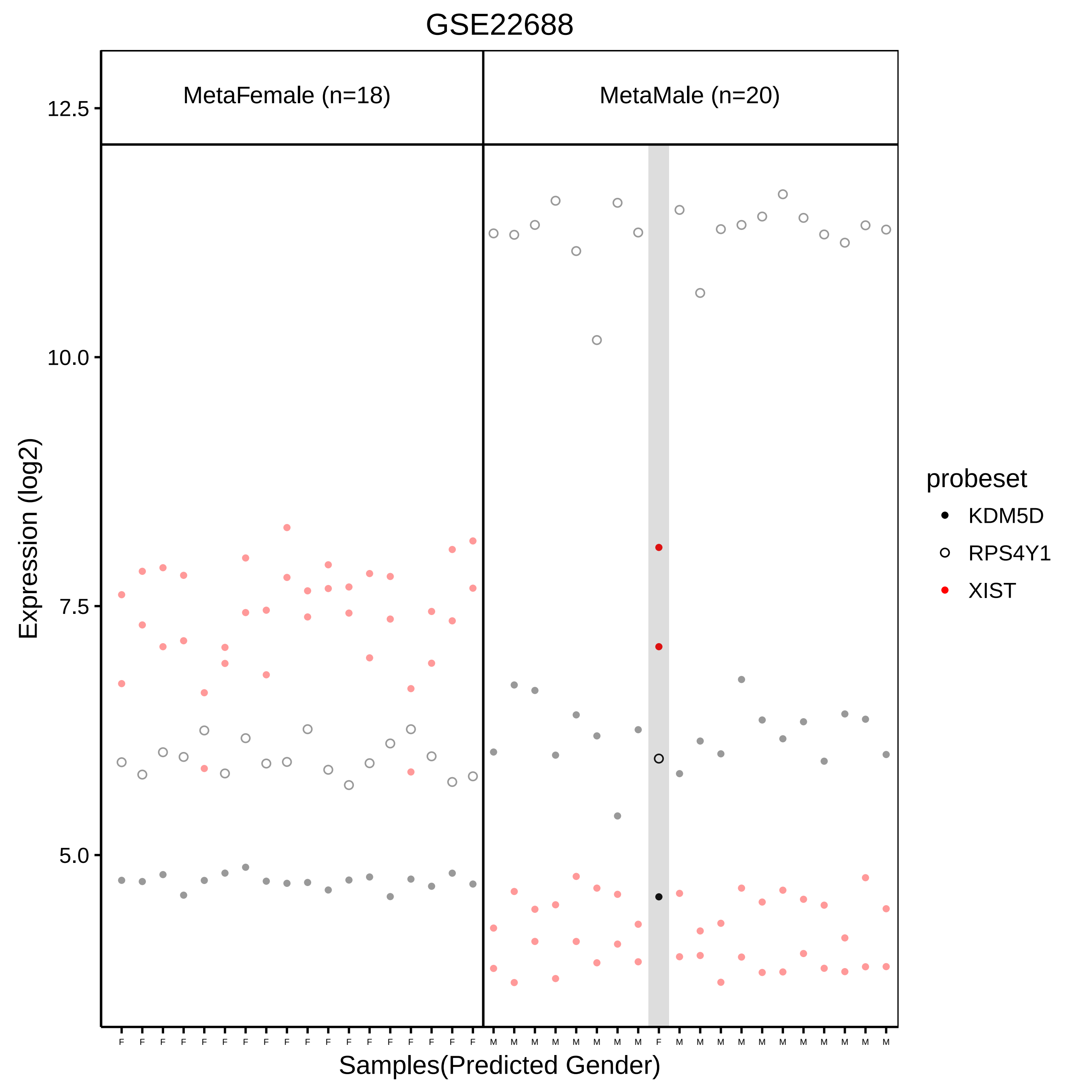

GSE22688
MetaFemale (n=18)
MetaMale (n=20)
12.5
10.0
7.5
5.0
Expression (log2)
Samples(Predicted Gender)
F F F F F F F F F F F F F F F F F F M M M M M M M M F M M M M M M M M M M M
probeset
KDM5D
RPS4Y1
XIST

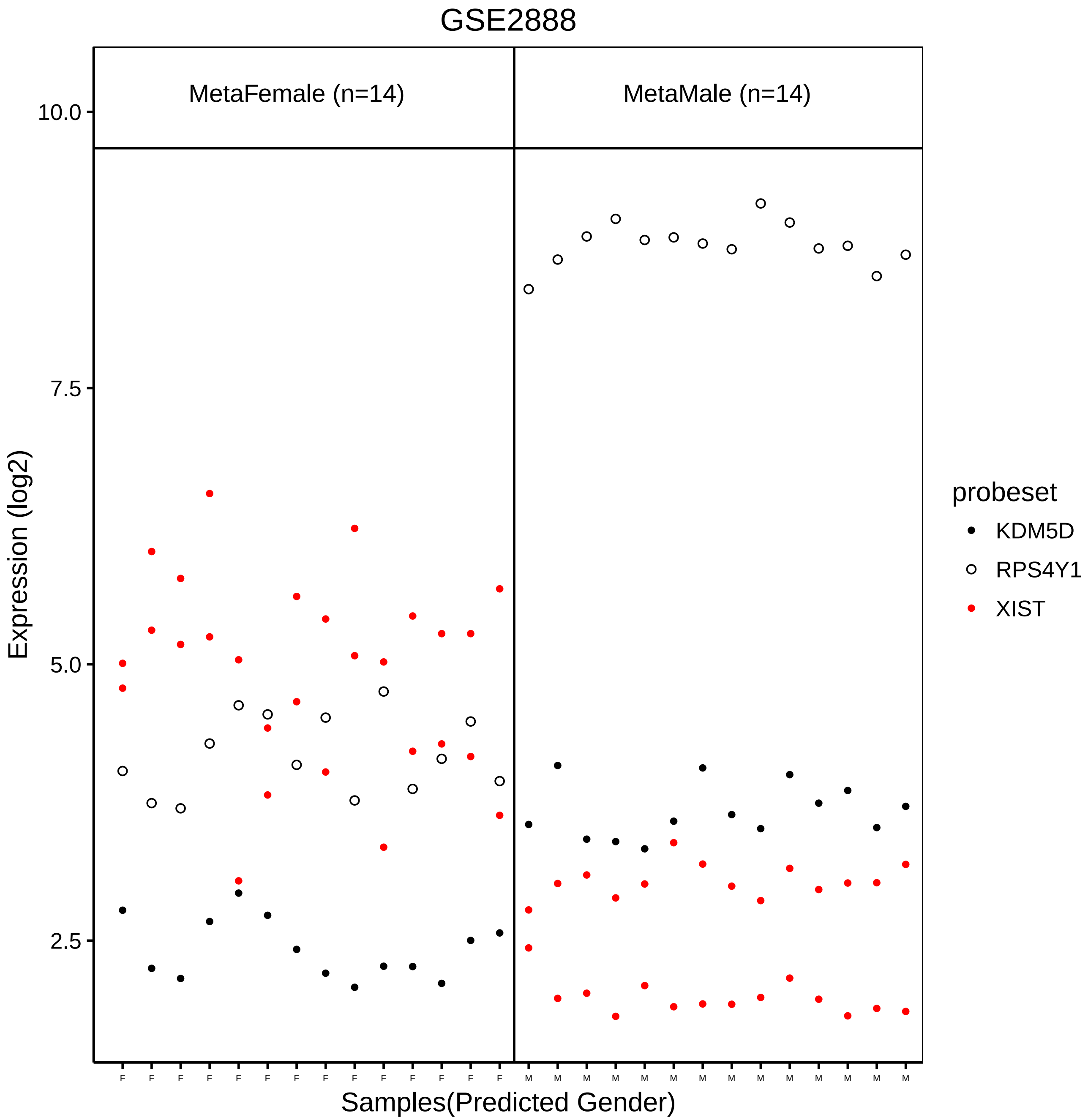

GSE2888
MetaFemale (n=14)
MetaMale (n=14)
10.0
7.5
5.0
2.5
Expression (log2)
Samples(Predicted Gender)
F F F F F F F F F F F F F F
M M M M M M M M M M M M M M
probeset
KDM5D
RPS4Y1
XIST

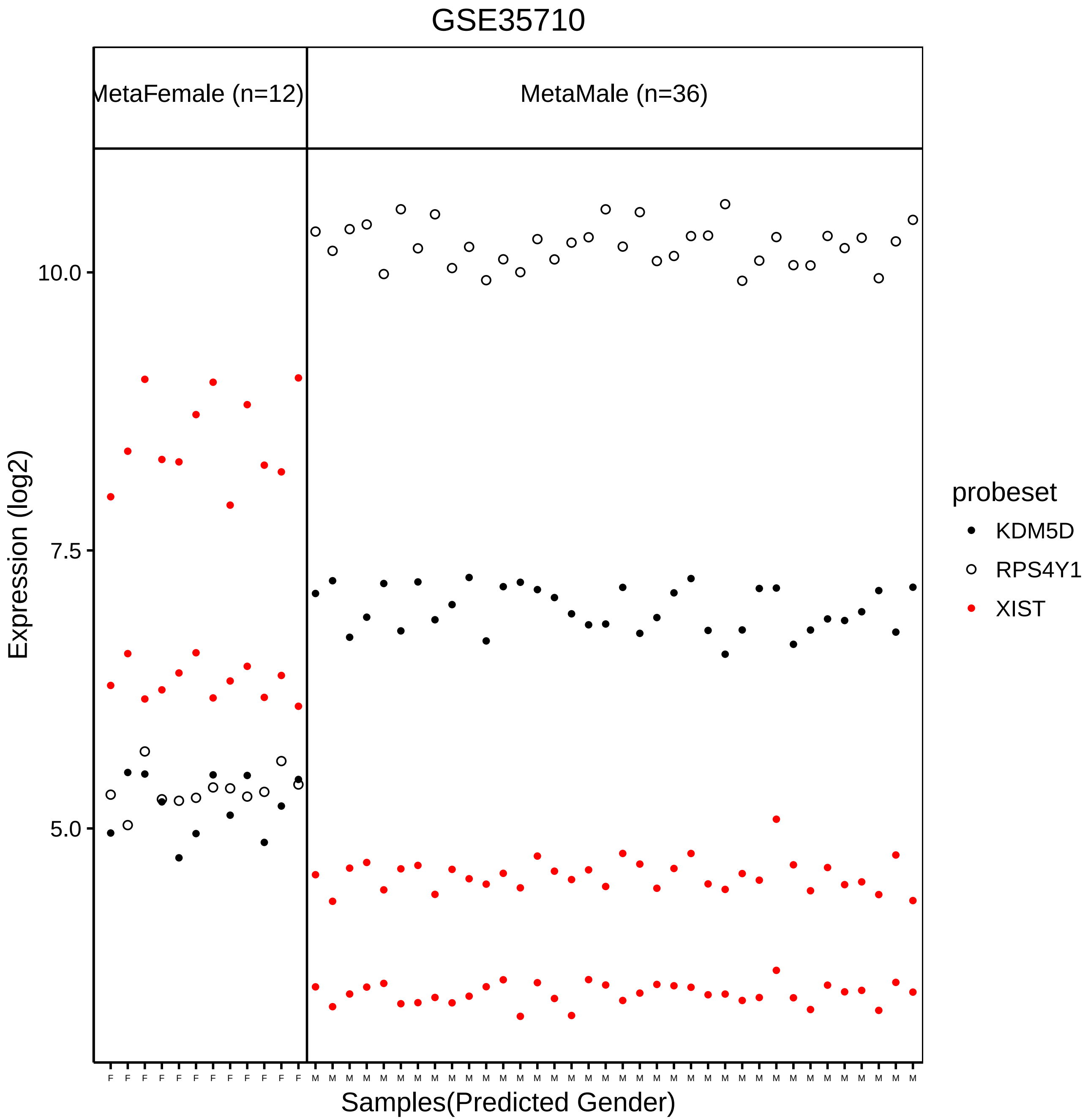

GSE35710
MetaFemale (n=12)
MetaMale (n=36)
Expression (log2)
10.0
7.5
5.0
Samples(Predicted Gender)
probeset
KDM5D
RPS4Y1
XIST

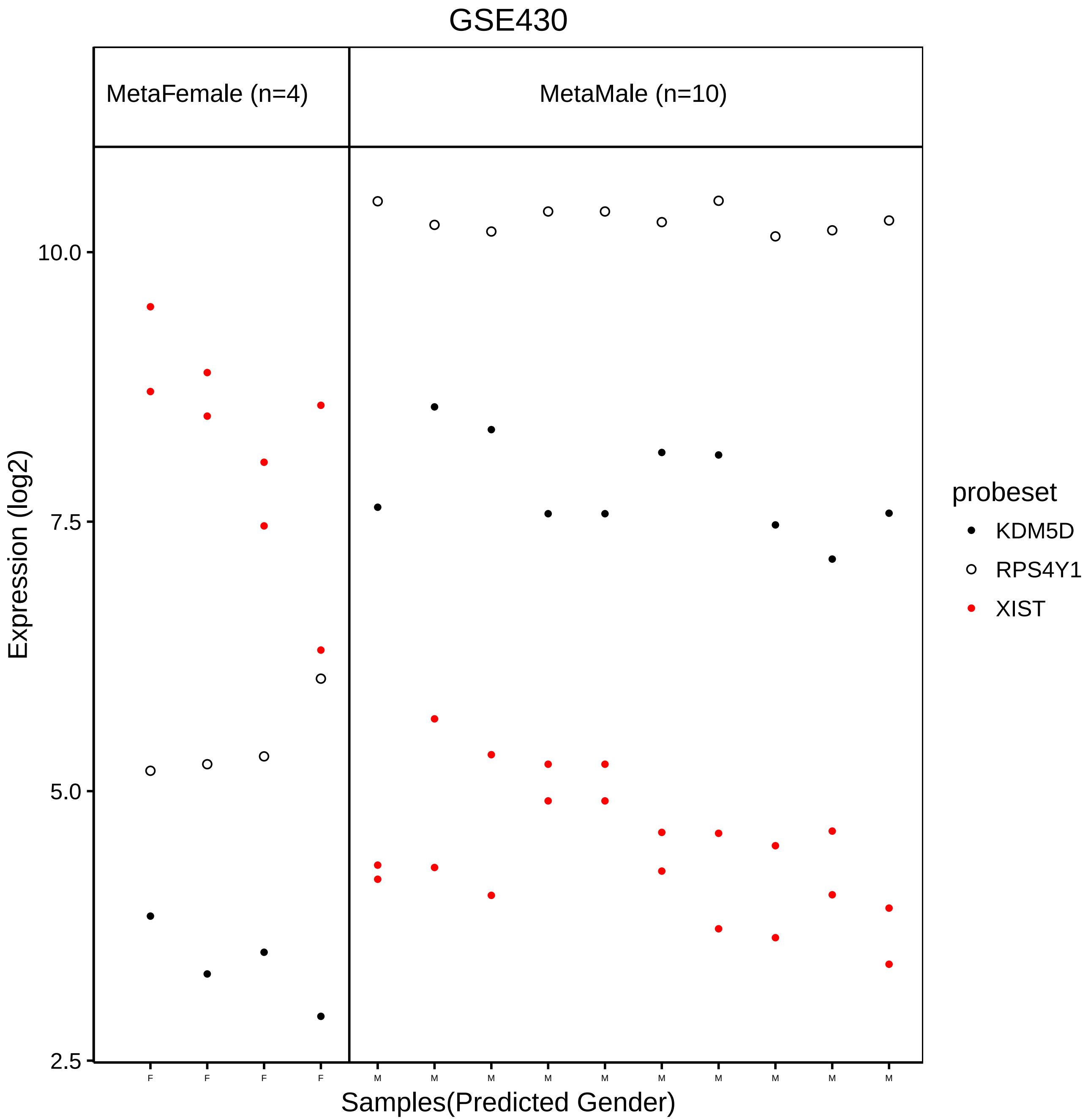
GSE430
MetaFemale (n=4)
MetaMale (n=10)
Expression (log2)
10.0
7.5
5.0
2.5
F
F
F
F
M
M
M
M
M
M
M
M
M
M
Samples(Predicted Gender)
probeset
KDM5D
RPS4Y1
XIST

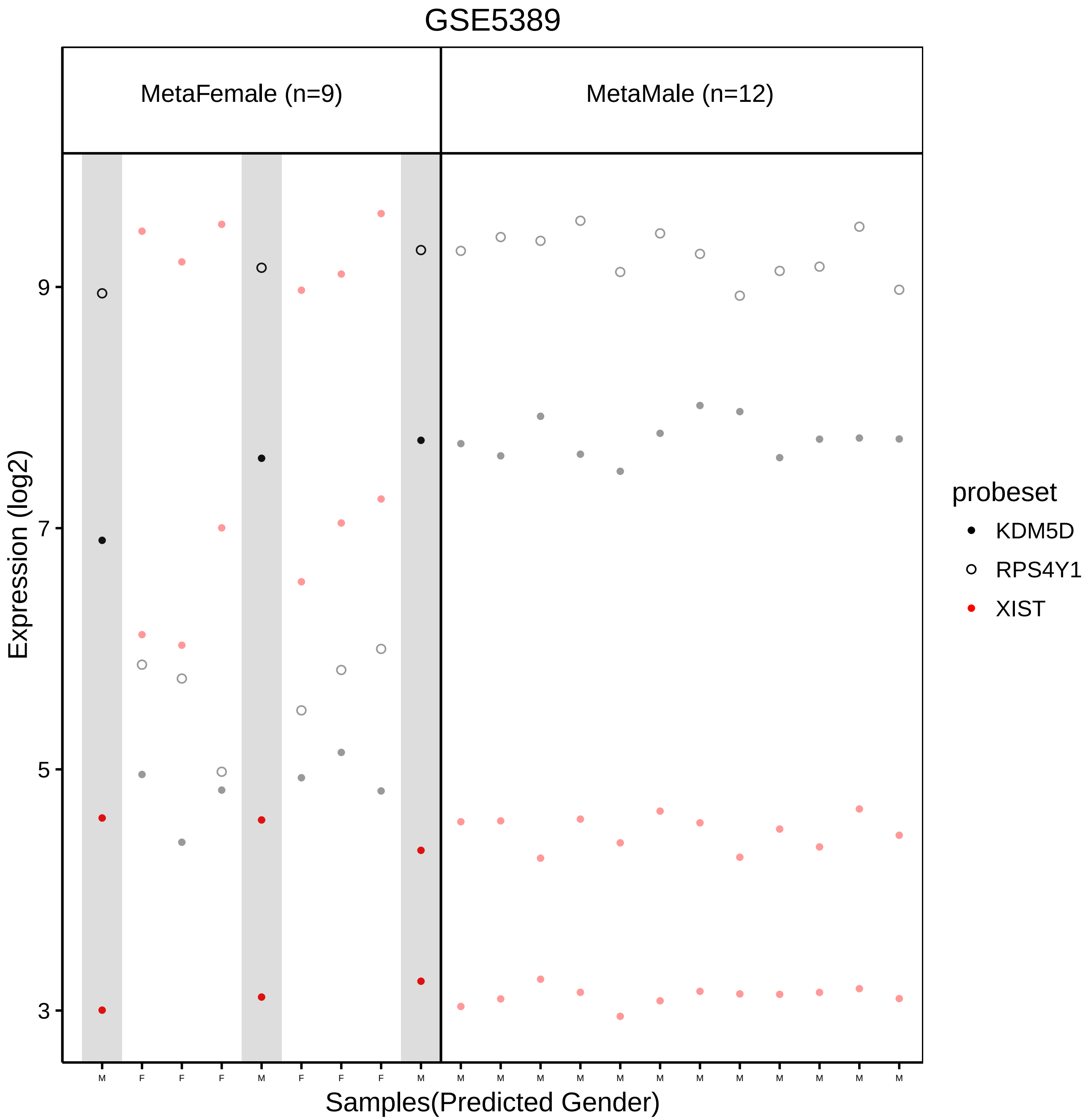

GSE5389
MetaFemale (n=9)
MetaMale (n=12)
Expression (log2)
9
7
5
3
M F F F M F F F M M M M M M M M M M M M
Samples(Predicted Gender)
probeset
KDM5D
RPS4Y1
XIST

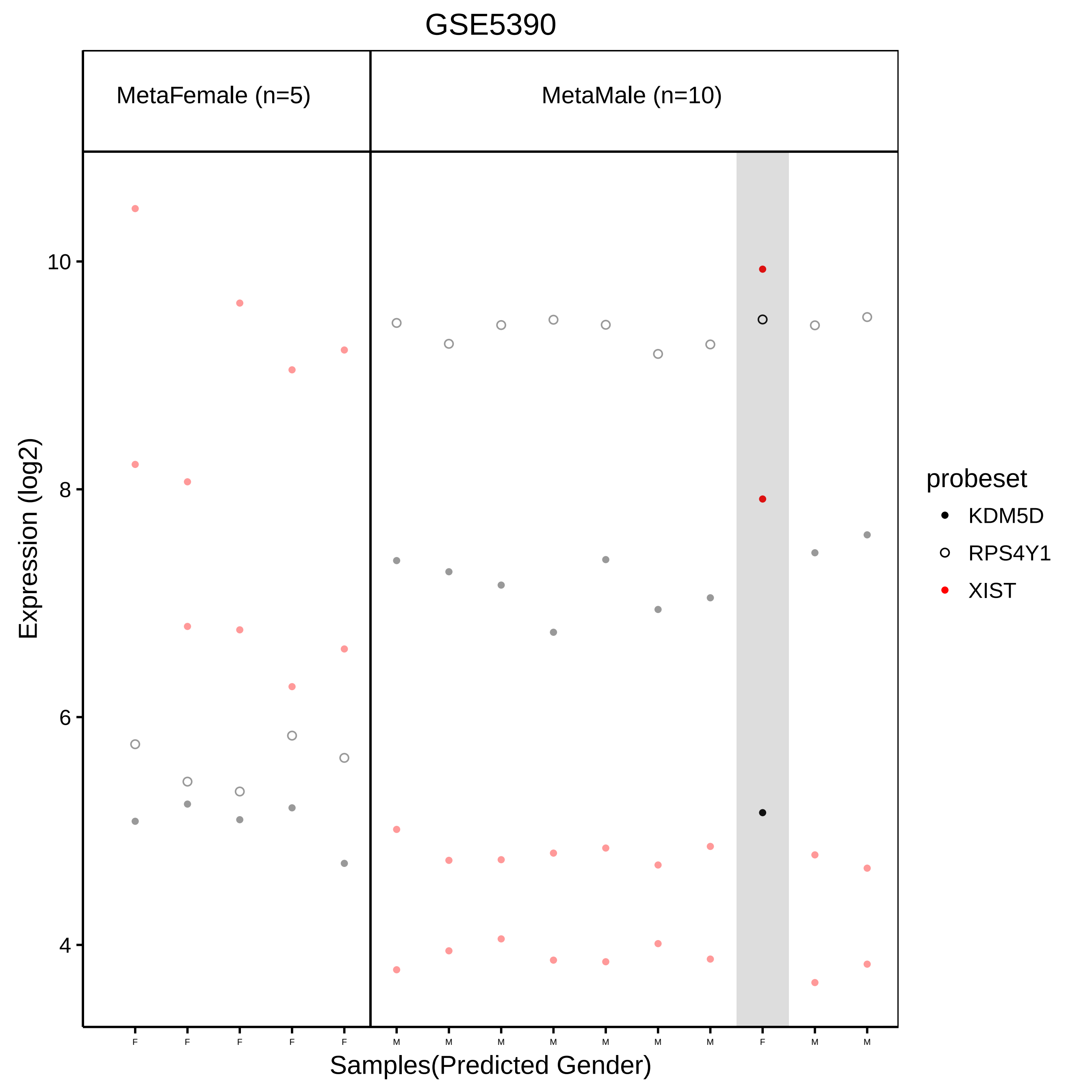

GSE5390
MetaFemale (n=5)
MetaMale (n=10)
Expression (log2)
10
8
6
4
F F F F F M M M M M M M F M M
Samples(Predicted Gender)
probeset
KDM5D
RPS4Y1
XIST

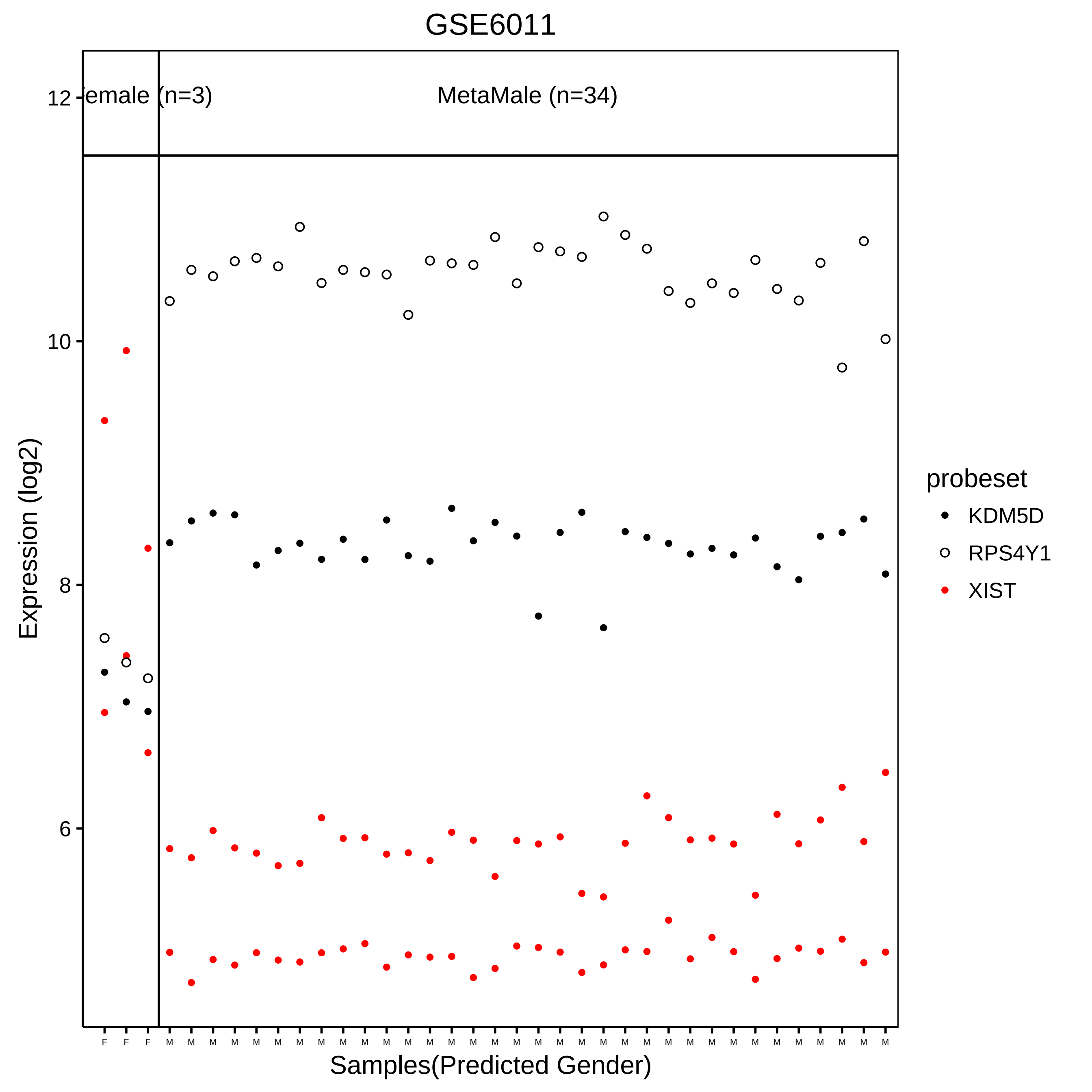

GSE6011
Female (n=3)
MetaMale (n=34)
Expression (log2)
12
10
8
6
Samples(Predicted Gender)
F
F
F
M
probeset
KDM5D
RPS4Y1
XIST

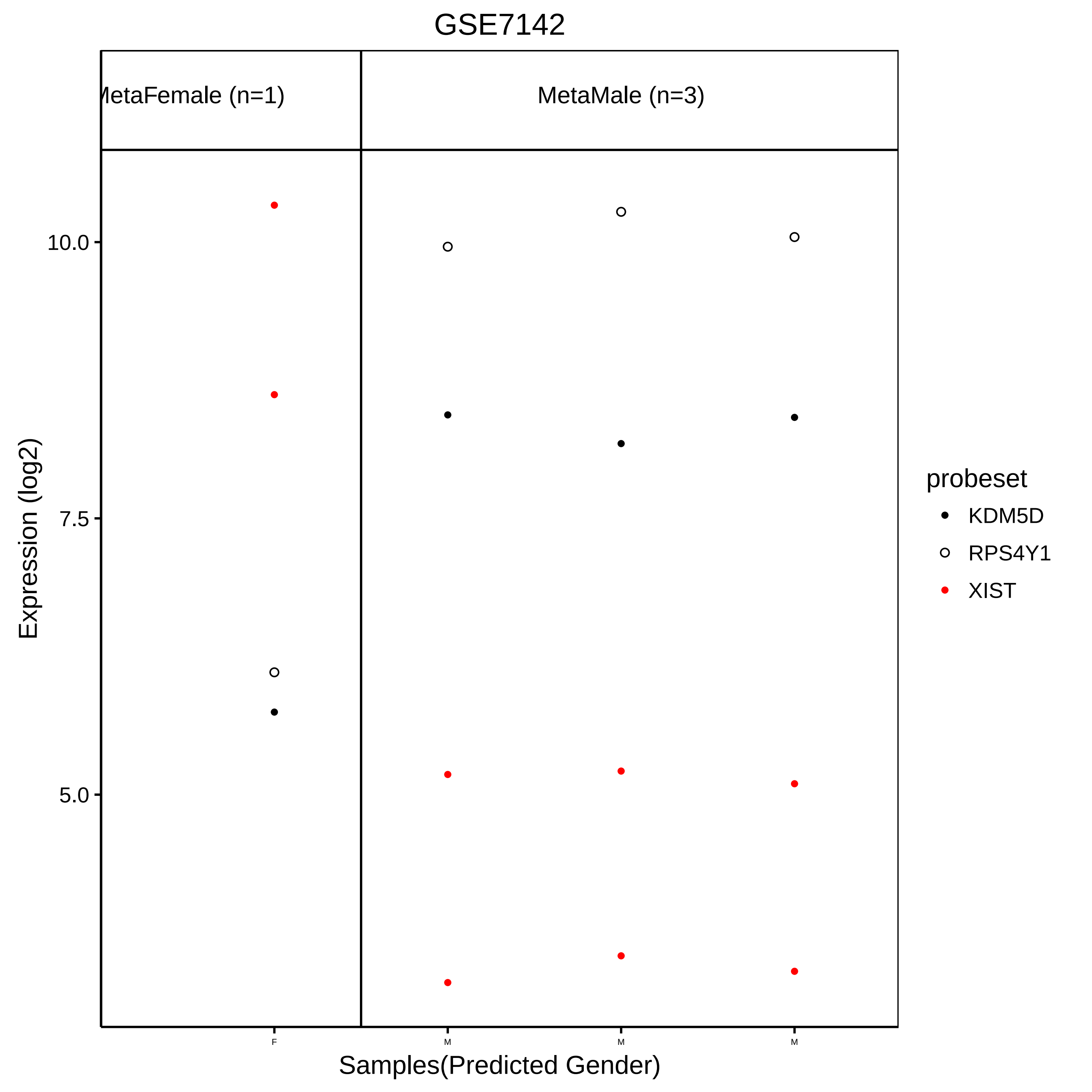

GSE7142
MetaFemale (n=1)
MetaMale (n=3)
10.0
7.5
5.0
Expression (log2)
F
M
M
M
Samples(Predicted Gender)
probeset
KDM5D
RPS4Y1
XIST

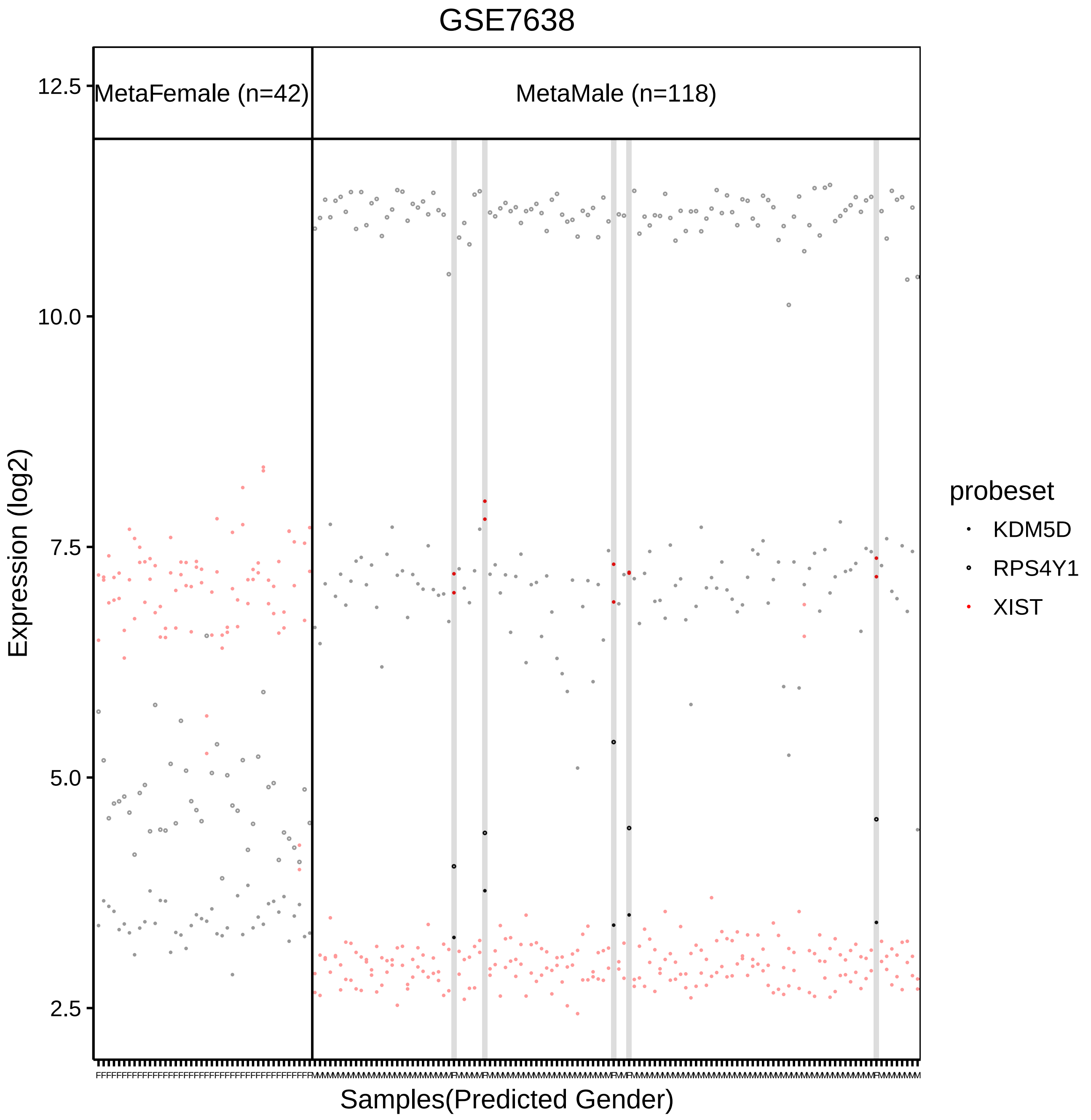
GSE7638
MetaFemale (n=42)
MetaMale (n=118)
12.5
10.0
7.5
5.0
2.5
Expression (log2)
Samples(Predicted Gender)
probeset
KDM5D
RPS4Y1
XIST

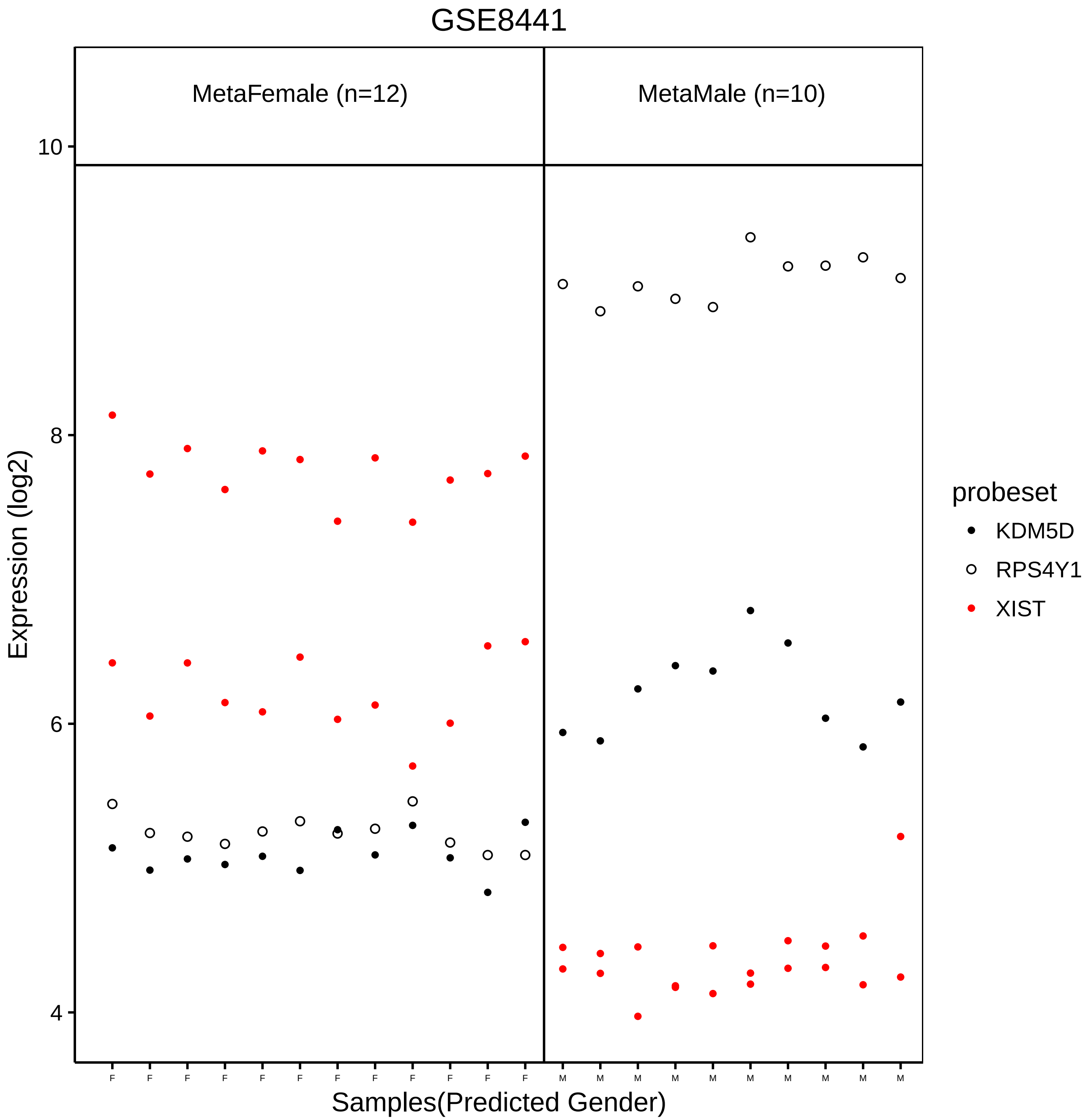

GSE8441
MetaFemale (n=12)
MetaMale (n=10)
Expression (log2)
10
8
6
4
F F F F F F F F F F F F
M M M M M M M M M M
Samples(Predicted Gender)
probeset
KDM5D
RPS4Y1
XIST

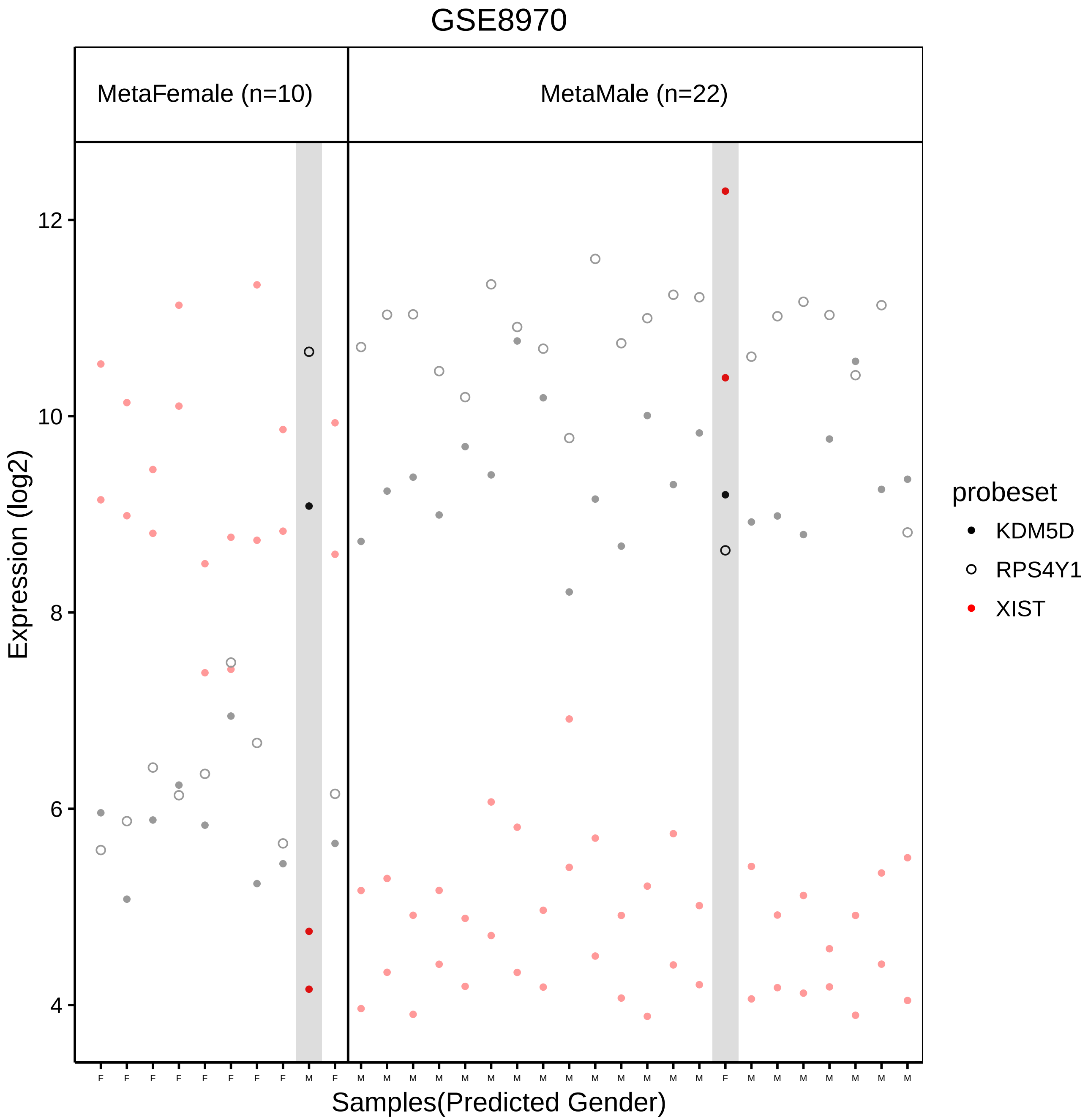
GSE8970
MetaFemale (n=10)
MetaMale (n=22)
Expression (log2)
12
10
8
6
4
F
F
F
F
F
F
F
F
M
F
M
M
M
M
M
M
M
M
M
M
M
M
M
M
F
M
M
M
M
M
M
M
Samples(Predicted Gender)
probeset
KDM5D
RPS4Y1
XIST

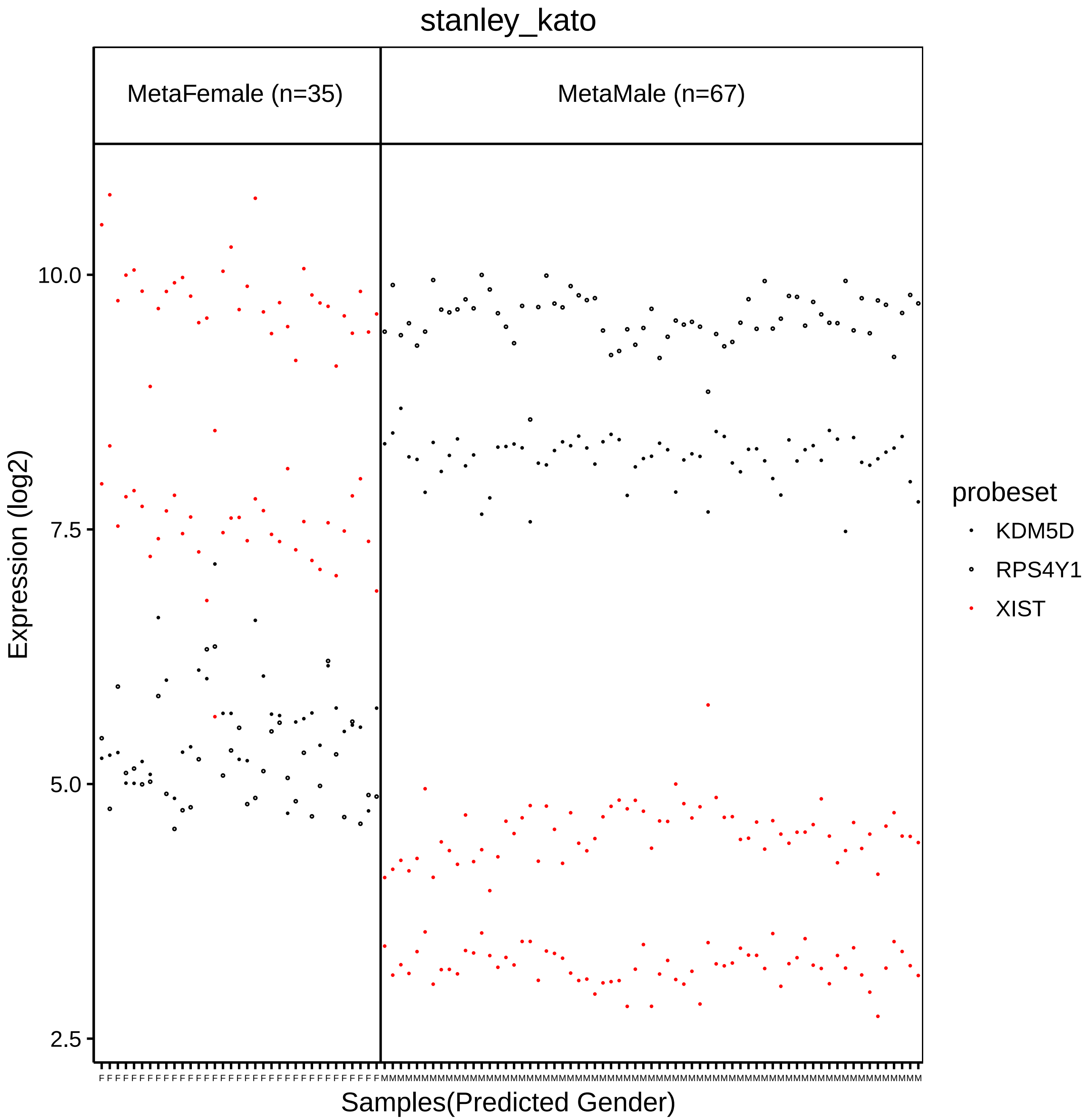

stanley_kato
MetaFemale (n=35)
MetaMale (n=67)
Expression (log2)
10.0
7.5
5.0
2.5
Samples(Predicted Gender)
probeset
KDM5D
RPS4Y1
XIST

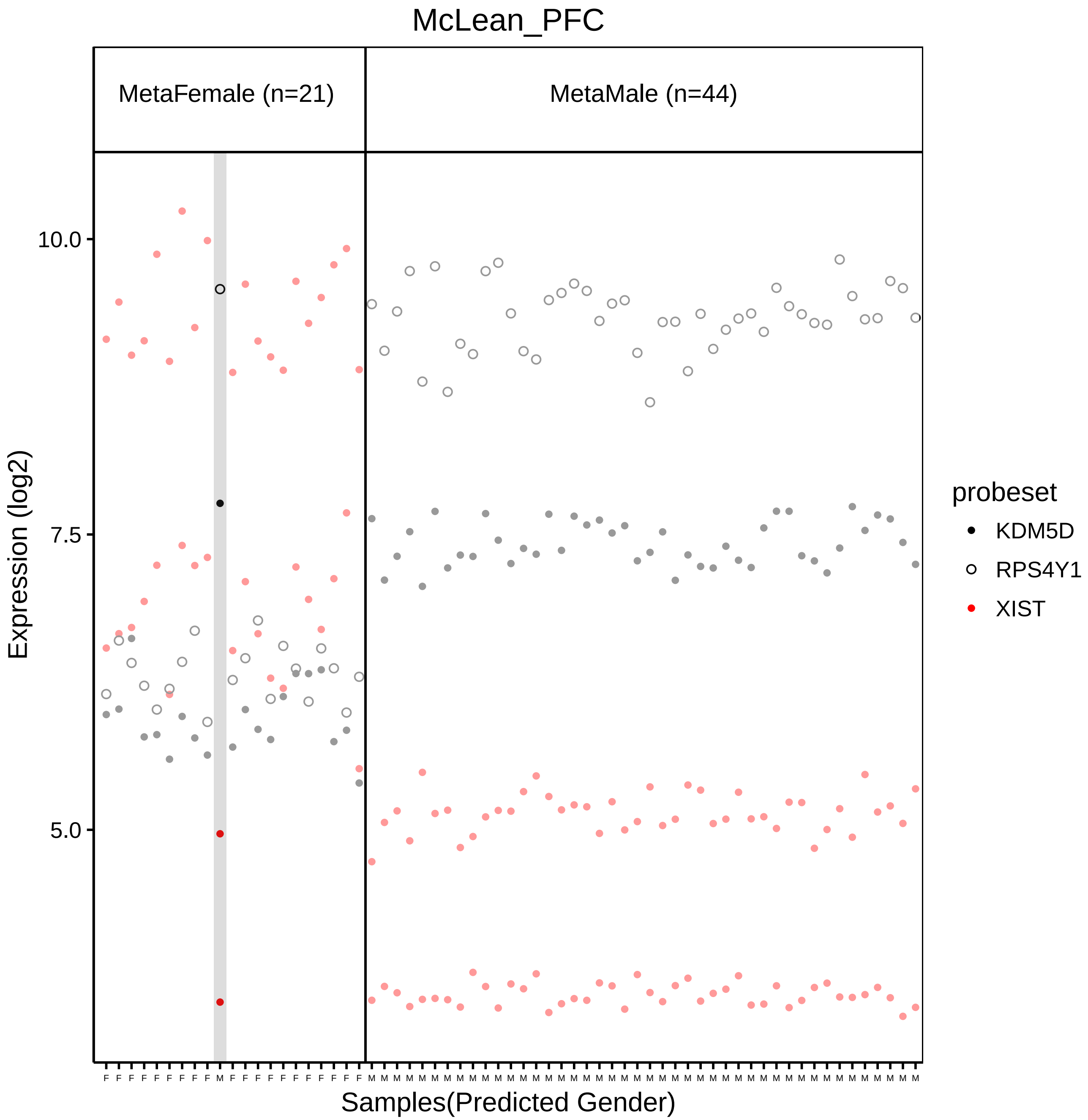

McLean_PFC
MetaFemale (n=21)
MetaMale (n=44)
Expression (log2)
10.0
7.5
5.0
Samples(Predicted Gender)
probeset
KDM5D
RPS4Y1
XIST

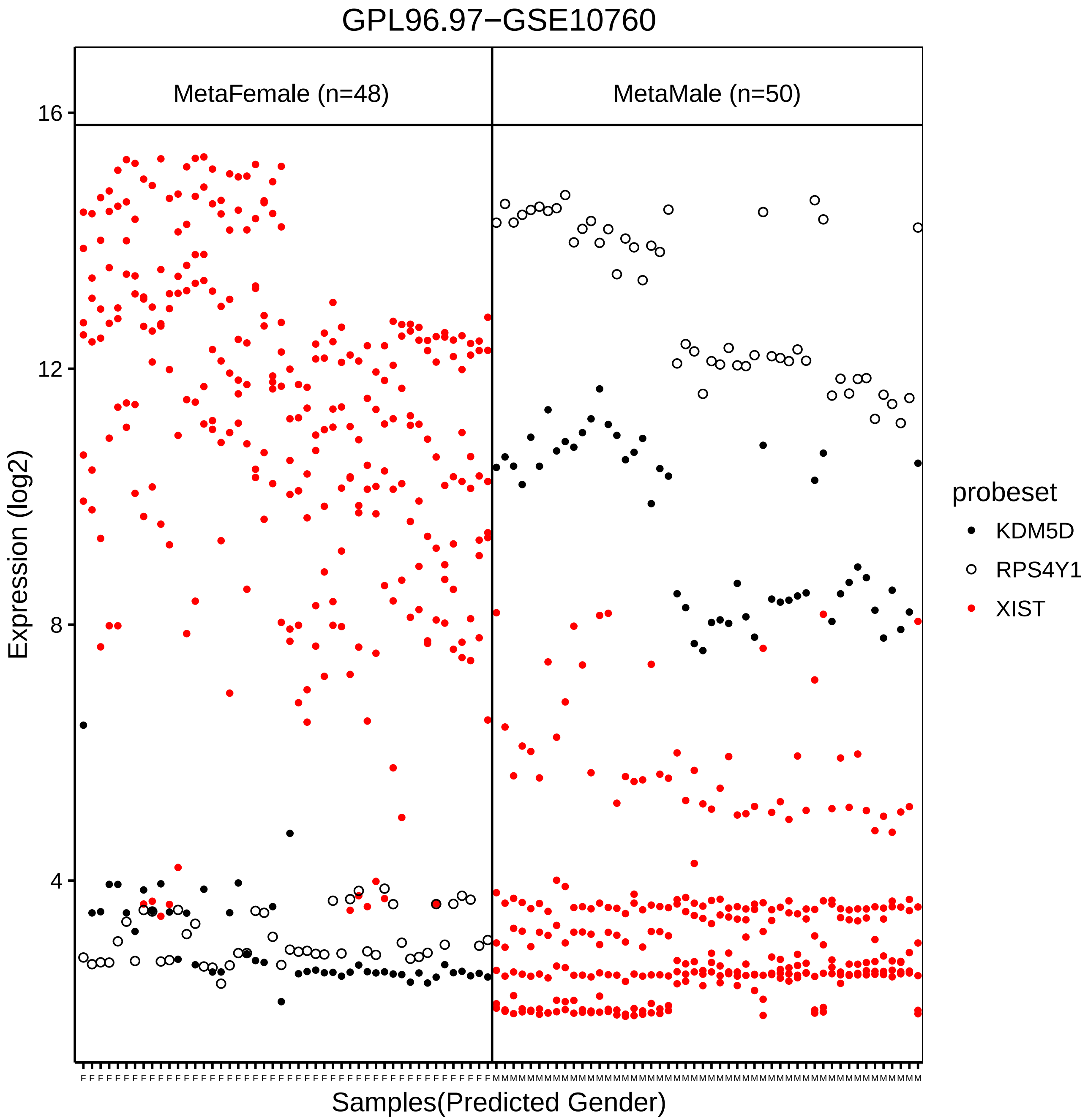
GPL96.97−GSE10760
MetaFemale (n=48)
MetaMale (n=50)
16
12
8
4
Expression (log2)
Samples(Predicted Gender)
probeset
KDM5D
RPS4Y1
XIST

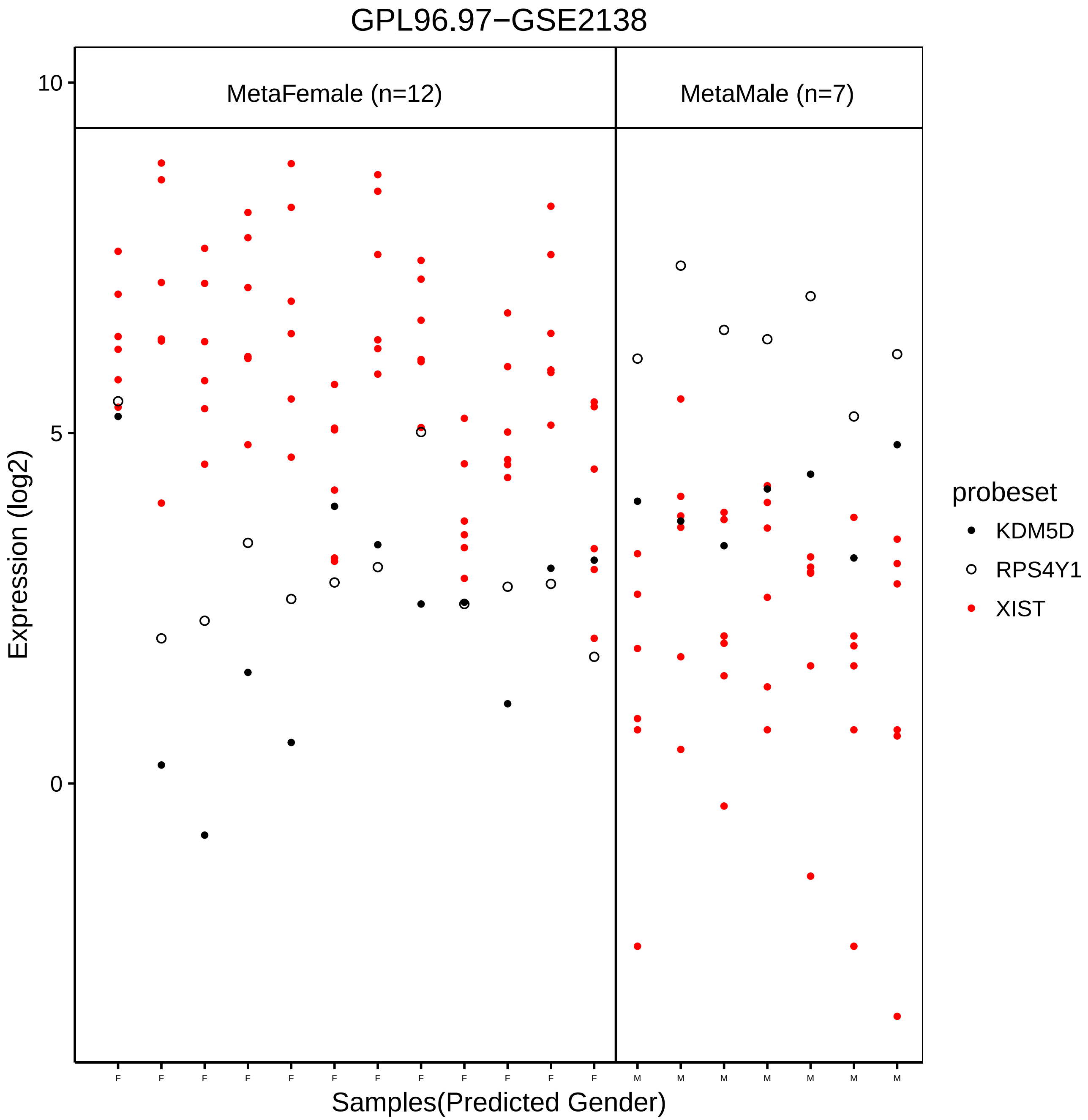

GPL96.97−GSE2138
MetaFemale (n=12)
MetaMale (n=7)
Expression (log2)
10
5
0
Samples(Predicted Gender)
F
M
probeset
KDM5D
RPS4Y1
XIST

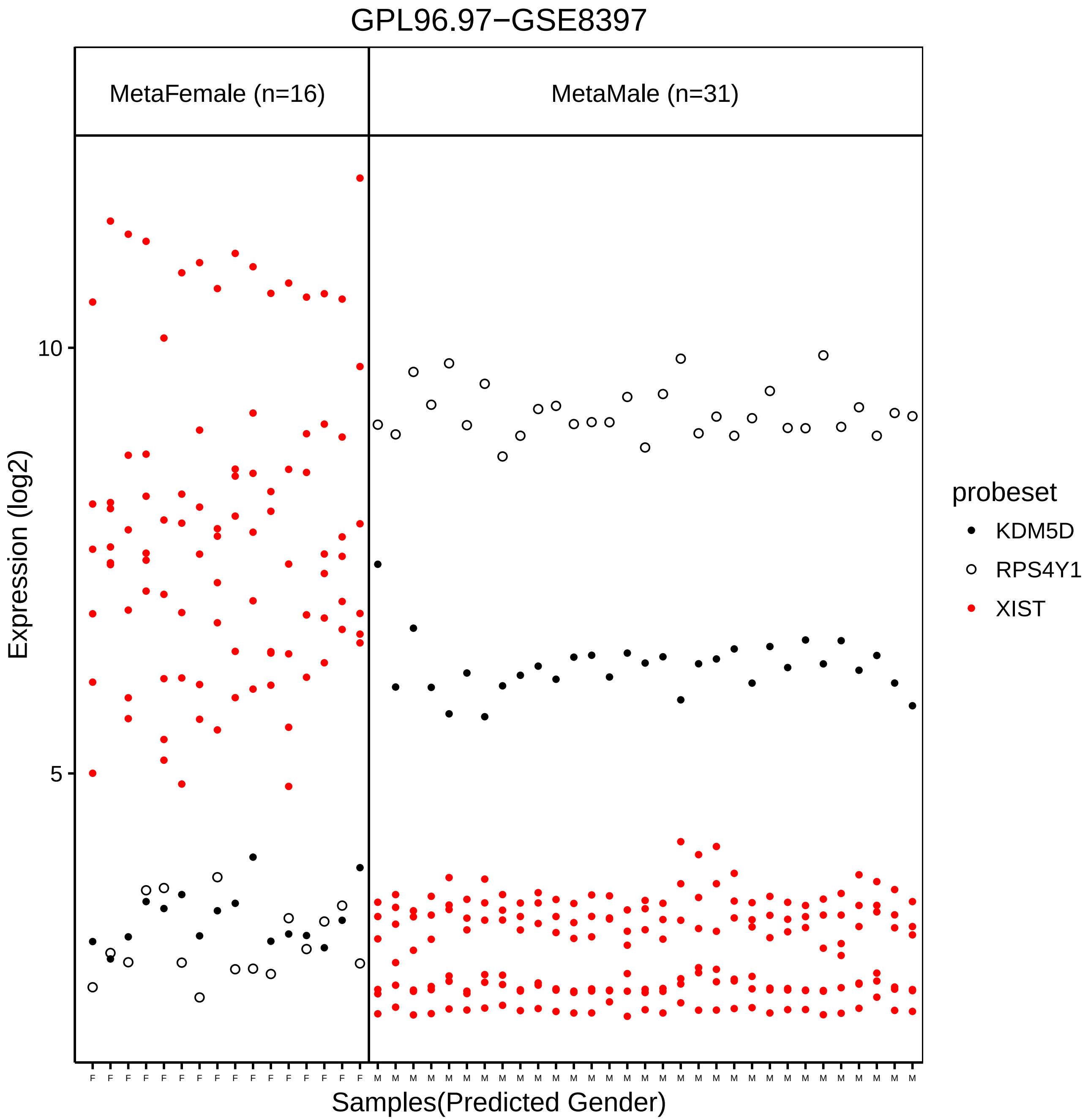

GPL96.97−GSE8397
MetaFemale (n=16)
MetaMale (n=31)
Expression (log2)
10
5
Samples(Predicted Gender)
probeset
KDM5D
RPS4Y1
XIST

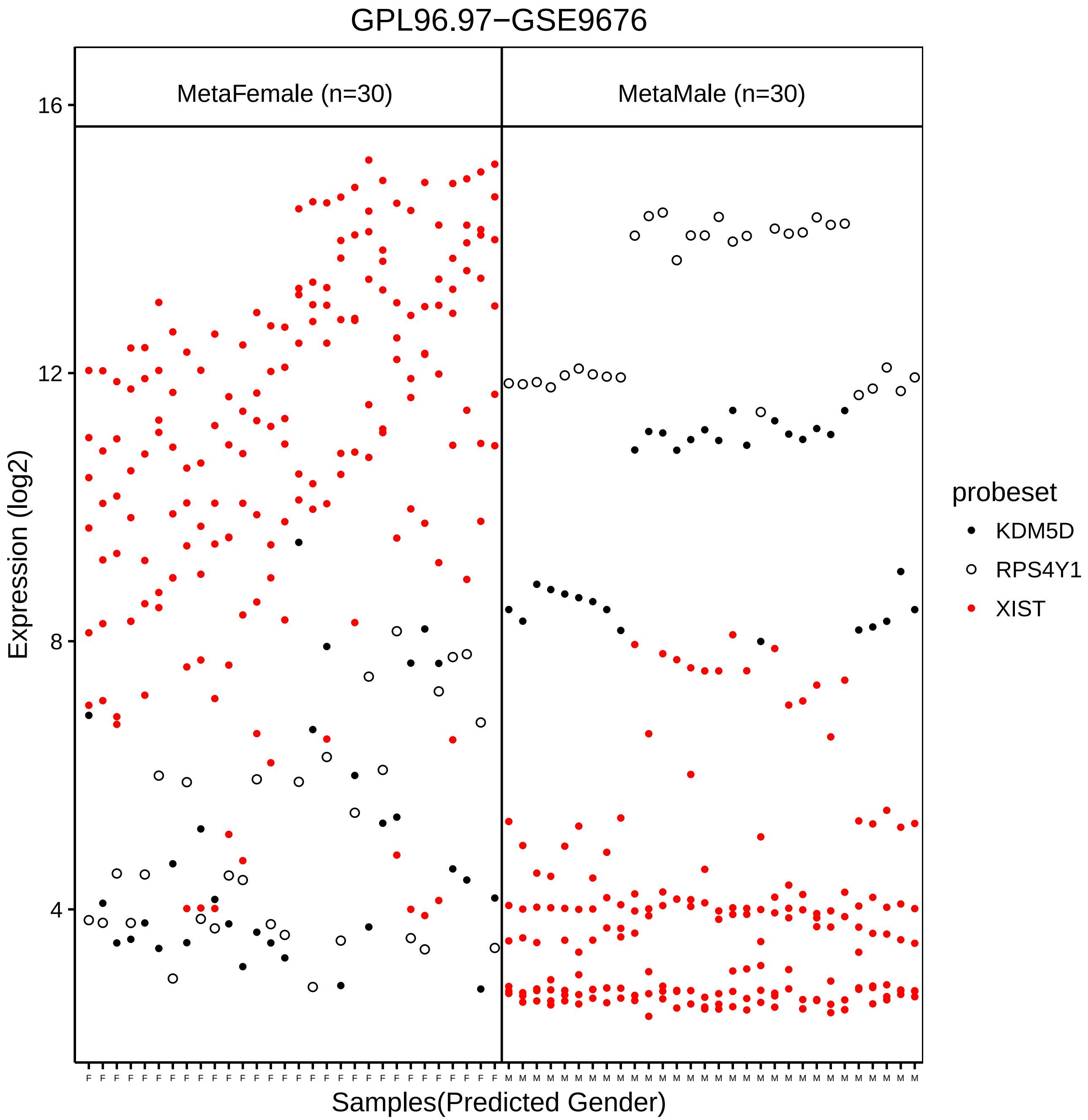

GPL96.97−GSE9676
MetaFemale (n=30)
MetaMale (n=30)
Expression (log2)
16
12
8
4
Samples(Predicted Gender)
probeset
KDM5D
RPS4Y1
XIST
