# Supplementary material for: Whose sample is it anyway? Widespread misannotation of samples in transcriptomics studies
Source: F1000Res. 2016 Sep 30;5:2103. Originally published 2016 Aug 30. [Version 2] doi: 10.12688/f1000research.9471.2 (PMC5034794; doi:10.12688/f1000research.9471.2)
Supplement: Peer review history

# Peer review: Referee response for version 2

**DOI:** 10.5256/f1000research.10437.r16396

**Published:** 2016-10-03

**Authors:** Levi Waldron

**Affiliations:** 1 School of Public Health, City University of New York, New York, NY, USA

The article is clear and to the point, and the abstract provides an adequate summary of the article, the methods are adequately described, conclusions are balanced and justified, and data are available in a usable format. I do have a few comments though:

1. I have a concern that the use of kmeans clustering assigns individuals to one of the two clusters without any estimate of posterior probability of the assignment. Although the examples shown in Figure 1 imply there is little uncertainty in these assignments, it is unclear whether that is always the case, or whether there are some more ambiguous cases. It would be much more reassuring to use a clustering algorithm that allows estimation of posterior probability of cluster assignment (such as K-nearest neighbors in the "classify" package). Then I would immediately ask, are the biological / annotation mismatches all called with high confidence (say, >99%), or are some ambiguous (e.g. 49%)?
2. The article could have greater impact if the authors provided a ready-to-use tool for others to do the same check. It doesn't have to be fancy; it could be provided even just by providing .RData objects for the fitted classifer object used for the clustering.
3. The "How to replicate my analysis" section on https://github.com/min110/mislabeled.samples.identification README.md is blank, and should be filled in.
4. PURELY OPTIONAL: a paper of mine also points out the frequency of sample duplication, including an instance of several dozen RNA aliquot mix-ups in TCGA [1], should you find it relevant to cite.

I have read this submission. I believe that I have an appropriate level of expertise to confirm that it is of an acceptable scientific standard, however I have significant reservations, as outlined above.

## References

[1] WaldronLRiesterMRamosMParmigianiGBirrerM: The Doppelgänger Effect: Hidden Duplicates in Databases of Transcriptome Profiles. *J Natl Cancer Inst*.2016;108(11) : 10.1093/jnci/djw146 10.1093/jnci/djw146 27381624PMC5241903

# Peer review: Referee response for version 1

**DOI:** 10.5256/f1000research.10200.r16144

**Published:** 2016-09-22

**Authors:** Hans van Bokhoven

**Affiliations:** 1 Department of Human Genetics & Department of Cognitive Neuroscinders Institute for Brain, Cognition and Behaviour ences, Radboud University Medical Center , Nijmegen, Netherlands

This article by Toker *et al.* reports a retrospective biostatistical analysis of the data reported in 70 RNA expression studies to identify the possible misannotation of samples used in these studies. Their analysis is based on the expression of gender-specific genes (XIST for females; KDM5D and RPS4Y1 for males). Their analysis revealed apparent mismatches between the expression data and the annotated gender for 83 of 4160 samples (2%), encompassing 32 of the 70 studies (46%). This percentage is consistent with a those of a previous analysis in cancer datasets (PUBMED: 26608184). While these figures are already alarming, the actual number of mismatches is likely to be higher, because the gender-analysis can only identify discrepancies based on a gender-mismatch and will not detect mislabelling of samples of the same gender and case-control samples.

 For most cases, the reason for the mismatches is not clear, but comparison of identical sample-data presented in different publications revealed that sample mix-ups are likely to be involved.

 The mislabelling of samples in transcriptomics studies have an immediate impact on the involved studies, which often only have a modest sample size. In addition, also follow-up studies based on the results reported in such studies can suffer from it. Therefore, the use of controls to check the identity of samples is warranted. The gender-test presented in this work is a simple test that should become routine in expression studies, another option is to use nucleic acid-based bar codes that can be added to the sample early in the processing.

 Some minor comments:

- On page 4, second-last line: here 29/31 of the affected studies is mentioned. Shouldn't that be 32? I hope I missed it, but certainly in a report as this one, the figures should be absolutely correct.
- Some typo's in the legend: Figure 2, Stady 5 is Study 5; Supplementary Fig S1: Based on the sex-genes expression, dataset GSE22491 contains at two 2 mislabelled samples.

I have read this submission. I believe that I have an appropriate level of expertise to confirm that it is of an acceptable scientific standard.

## References

[1] LohrMHellwigBEdlundKMattssonJSBotlingJSchmidtMHengstlerJGMickePRahnenführerJ: Identification of sample annotation errors in gene expression datasets. *Arch Toxicol*.2015;89(12) : 10.1007/s00204-015-1632-4 2265-72 10.1007/s00204-015-1632-4 26608184PMC4673097

# Peer review: Referee response for version 1

**DOI:** 10.5256/f1000research.10200.r15949

**Published:** 2016-09-13

**Authors:** Leonard P. Freedman

**Affiliations:** 1 Global Biological Standards Institute, Washington, DC, USA

This is an excellent paper highlighting the importance of sample annotation as a critical contributor to reproducible research. Using transcriptomics of sex-specific gene expression levels as an example, the authors do a careful analysis to illustrate the issue of mislabeling. My one concern, which they candidly acknowledge, is that nearly half of the samples appear to have only one error, which may lead many readers to conclude that this actually a quite respectable error rate and that thus this is not really a big problem. It was not entirely clear to me why, as the authors argue, that this is in fact 'the tip of the iceberg".

I have read this submission. I believe that I have an appropriate level of expertise to confirm that it is of an acceptable scientific standard.
